# DNA methylation age of human tissues and cell types

**DOI:** 10.1186/gb-2013-14-10-r115

**Published:** 2013-10-21

**Authors:** Steve Horvath

**Affiliations:** 1 Human Genetics, David Geffen School of Medicine, University of California Los Angeles, Los Angeles, CA 90095, USA; 2 Biostatistics, School of Public Health, University of California Los Angeles, Los Angeles, CA 90095, USA; 3 Human Genetics, Gonda Research Center, David Geffen School of Medicine, University of California Los Angeles, Los Angeles, CA 90095-7088, USA

## Abstract

**Background:**

It is not yet known whether DNA methylation levels can be used to accurately predict age across a broad spectrum of human tissues and cell types, nor whether the resulting age prediction is a biologically meaningful measure.

**Results:**

I developed a multi-tissue predictor of age that allows one to estimate the DNA methylation age of most tissues and cell types. The predictor, which is freely available, was developed using 8,000 samples from 82 Illumina DNA methylation array datasets, encompassing 51 healthy tissues and cell types. I found that DNA methylation age has the following properties: first, it is close to zero for embryonic and induced pluripotent stem cells; second, it correlates with cell passage number; third, it gives rise to a highly heritable measure of age acceleration; and, fourth, it is applicable to chimpanzee tissues. Analysis of 6,000 cancer samples from 32 datasets showed that all of the considered 20 cancer types exhibit significant age acceleration, with an average of 36 years. Low age-acceleration of cancer tissue is associated with a high number of somatic mutations and *TP53* mutations, while mutations in steroid receptors greatly accelerate DNA methylation age in breast cancer. Finally, I characterize the 353 CpG sites that together form an aging clock in terms of chromatin states and tissue variance.

**Conclusions:**

I propose that DNA methylation age measures the cumulative effect of an epigenetic maintenance system. This novel epigenetic clock can be used to address a host of questions in developmental biology, cancer and aging research.

## Background

An increasing body of evidence suggests that many manifestations of aging are epigenetic [1,2]. This article focuses on one particular type of epigenetic control: cytosine-5 methylation within CpG dinucleotides (also known as DNA methylation). Age-related DNA hypomethylation has long been observed in a variety of species, including salmon [3], rats [4], and mice [5]. More recent studies have shown that many CpGs are subject to age-related hypermethylation. A vast literature characterizes genes or genomic regions that either get hypermethylated or hypomethylated with age [6-14]. Previous studies have shown that age-related hypermethylation occurs preferentially at CpG islands [8], at bivalent chromatin domain promoters that are associated with key developmental genes [15], and at Polycomb-group protein targets [10]. The epigenomic landscape varies markedly across tissue types [16-18] and many age-related changes depend on tissue type [8,19]. But several recent studies have shown that age-dependent CpG signatures can be defined independently of sex, tissue type, disease state and array platform [10,13-15,20-22]. While several recent articles describe age predictors based on DNAm levels in specific tissues (for example, saliva or blood [23,24]), it is not yet known whether age can be predicted irrespective of tissue type using a single predictor. Here I use an unprecedented collection of publicly available DNA methylation data sets for defining and evaluating an age predictor. Its astonishing accuracy across most tissues and cell types justifies its designation as a multi-tissue age predictor. Its age prediction, referred to as DNAm age, can be used as a biomarker for addressing a host of questions arising in aging research and related fields. For example, I show that premature aging diseases (such as progeria) do not resemble healthy normal aging according to DNAm age and that interventions used for creating induced pluripotent stem (iPS) cells reset the epigenetic clock to zero. I also describe what can be learnt from applying DNAm age to cancer tissues and cancer cell lines.

## Results and discussion

### Description of the (non-cancer) DNA methylation data sets

I assembled a large DNA methylation data set by combining publicly available individual data sets measured on the Illumina 27K or Illumina 450K array platform. In total, I analyzed n = 7,844 non-cancer samples from 82 individual data sets (Additional file 1), which assess DNA methylation levels in 51 different tissues and cell types. Although many data sets were collected for studying certain diseases (Additional file 2), they largely involved healthy tissues. In particular, cancer tissues were excluded from this first large data set since it is well known that cancer has a profound effect on DNA methylation levels [6,7,24-26]. The Cancer Genome Atlas (TCGA) data sets mentioned in Additional file 1 involved normal adjacent tissue from cancer patients. Details on the individual data sets and data pre-processing steps are provided in Materials and methods and Additional file 2. As described in Additional file 1, the first 39 data sets were used to construct ('train’) the age predictor. Data sets 40 to 71 were used to test (validate) the age predictor. Data sets 72 to 82 served other purposes - for example, to estimate the DNAm age of embryonic stem and iPS cells. The criteria used for selecting the training sets are described in Additional file 2. Briefly, the training data were chosen i) to represent a wide spectrum of tissues/cell types, ii) to involve samples whose mean age (43 years) is similar to that in the test data, and iii) to involve a high proportion of samples (37%) measured on the Illumina 450K platform since many on-going studies use this recent Illumina platform. Here I only studied 21,369 CpGs (measured with the Infinium type II assay) that were present on both Illumina platforms (Infinium 450K and 27K) and had fewer than 10 missing values across the data sets. Several important limitations of this study are discussed in Additional file 2.

### The multi-tissue age predictor used for defining DNAm age

To ensure an unbiased validation in the test data, I only used the training data to define the age predictor. As detailed in Materials and methods and Additional file 2, a transformed version of chronological age was regressed on the CpGs using a penalized regression model (elastic net). The elastic net regression model automatically selected 353 CpGs (Additional file 3). I refer to the 353 CpGs as (epigenetic) clock CpGs since their weighted average (formed by the regression coefficients) amounts to an epigenetic clock. Before characterizing them, I will show that the resulting age predictor performs remarkably well across a wide spectrum of tissues and cell types.

### Predictive accuracy across different tissues

I considered several measures of predictive accuracy since each measure has distinct advantages. The first, referred to as 'age correlation’, is the Pearson correlation coefficient between DNAm age (predicted age) and chronological age. It has the following limitations: it cannot be used for studying whether DNAm is well calibrated, it cannot be calculated in data sets whose subjects have the same chronological age (for example, cord blood samples from newborns), and it strongly depends on the standard deviation of age (as described below). The second accuracy measure, referred to as (median) 'error’, is the median absolute difference between DNAm age and chronological age. Thus, a test set error of 3.6 years indicates that DNAm age differs by less than 3.6 years in 50% of subjects. The error is well suited for studying whether DNAm age is poorly calibrated. 'Average age acceleration’, defined by the average difference between DNAm age and chronological age, can be used to determine whether the DNAm age of a given tissue is consistently higher (or lower) than expected.

**Figure 1:**
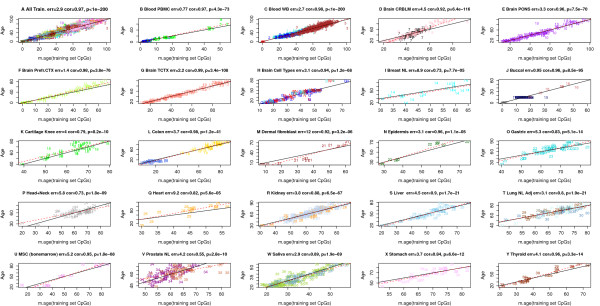
**Chronological age (y-axis) versus DNAm age (x-axis) in the training data.** Each point corresponds to a DNA methylation sample (human subject). Points are colored and labeled according to the underlying data set as described in Additional file 1. **(A)** Across all training data, the correlation between DNAm age (x-axis) and chronological age (y-axis) is 0.97 and the error (median absolute difference) is 2.9 years. Results for **(B)** peripheral blood mononuclear cells (cor = 0.97, error <1 year), **(C)** whole blood (cor = 0.98, error = 2.7 years), **(D)** cerebellum (cor = 0.92, error = 4.5), **(E)** pons (cor = 0.96, error = 3.3), **(F)** pre-frontal cortex (cor = 0.98, 1.4), **(G)** temporal cortex (cor = 0.99, error = 2.2), **(H)** brain samples, composed of 58 glial cell, 58 neuron cell, 20 bulk, and 9 mixed samples (cor = 0.94, error = 3.1), **(I)** normal breast tissue (cor = 0.73, error = 8.9), **(J)** buccal cells (cor = 0.95, error <1 year), **(K)** cartilage (cor = 0.79, error = 4), **(L)** colon (cor = 0.98, error = 3.7), **(M)** dermal fibroblasts (cor = 0.92, error = 12), **(N)** epidermis (cor = 0.96, error = 3.1), **(O)** gastric tissue (cor = 0.83, error = 5.3), **(P)** normal adjacent tissue from head/neck cancers (cor = 0.73, error = 5.8), **(Q)** heart (cor = 0.82, error = 9.2), **(R)** kidney (cor = 0.88, error = 3.8), **(S)** liver (cor = 0.90, error = 4.5), **(T)** lung (cor = 0.80, error = 3.1), **(U)** mesenchymal stromal cells (cor = 0.95, error = 5.2), **(V)** prostate (cor = 0.55, error = 4.2), **(W)** saliva (cor = 0.89, error = 2.9), **(X)** stomach (cor = 0.84, error = 3.7), **(Y)** thyroid (cor = 0.96, error = 4.1).

**Figure 2:**
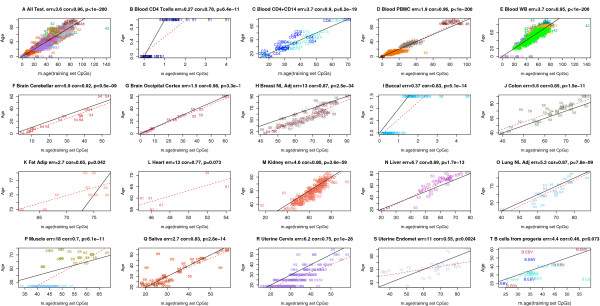
**Chronological age (y-axis) versus DNAm age (x-axis) in the test data. (A)** Across all test data, the age correlation is 0.96 and the error is 3.6 years. Results for **(B)** CD4 T cells measured at birth (age zero) and at age 1 (cor = 0.78, error = 0.27 years), **(C)** CD4 T cells and CD14 monocytes (cor = 0.90, error = 3.7), **(D)** peripheral blood mononuclear cells (cor = 0.96, error = 1.9), **(E)** whole blood (cor = 0.95, error = 3.7), **(F)** cerebellar samples (cor = 0.92, error = 5.9), **(G)** occipital cortex (cor = 0.98, error = 1.5), **(H)** normal adjacent breast tissue (cor = 0.87, error = 13), **(I)** buccal epithelium (cor = 0.83, error = 0.37), **(J)** colon (cor = 0.85, error = 5.6), **(K)** fat adipose (cor = 0.65, error = 2.7), **(L)** heart (cor = 0.77, error = 12), **(M)** kidney (cor = 0.86, error = 4.6), **(N)** liver (cor = 0.89, error = 6.7), **(O)** lung (cor = 0.87, error = 5.2), **(P)** muscle (cor = 0.70, error = 18), **(Q)** saliva (cor = 0.83, error = 2.7), **(R)** uterine cervix (cor = 0.75, error = 6.2), **(S)** uterine endometrium (cor = 0.55, 11), **(T)** various blood samples composed of 10 Epstein Barr Virus transformed B cell, three naive B cell, and three peripheral blood mononuclear cell samples (cor = 0.46, error = 4.4). Samples are colored by disease status: brown for Werner progeroid syndrome, blue for Hutchinson-Gilford progeria, and turquoise for healthy control subjects.

According to these three accuracy measures, the multi-tissue age predictor performs remarkably well in most tissues and cell types. Although its high accuracy in the training data (age correlation 0.97, error = 2.9 years; Figure 1) is probably overly optimistic, its performance assessment (age correlation = 0.96, error = 3.6 years; Figure 2) in the test data is unbiased. Note that the age predictor performs well in heterogeneous tissues (for example, whole blood, peripheral blood mononuclear cells, cerebellar samples, occipital cortex, buccal epithelium, colon, adipose, liver, lung, saliva, uterine cervix) as well as in individual cell types such as CD4 T cells and CD14 monocytes (Figure 2C) and immortalized B cells (Figure 2T).

The age predictor is particularly accurate in data sets composed of adolescents and children - for example, blood (Figures 1B and 2B; Additional file 4P,S), brain data (Figures 1F and 2F,G), and buccal epithelium (Figure 2I).

### The DNAm age of blood and brain cells

A detailed analysis of blood tissue can be found in Additional file 4. Human blood cells have different life spans: while CD14+ monocytes (myeloid lineage) only live several weeks, CD4+ T cells (lymphoid lineage) represent a variety of cell types that can live from months to years. An interesting question is whether blood cell types have different DNAm ages. DNAm age does not vary significantly across sorted blood cells from healthy male subjects (Additional file 4T). These results combined with the fact that the age predictor works well in individual cell types (Figure 2C; Additional file 4) strongly suggest that DNAm age does not reflect changes in cell type composition but rather intrinsic changes in the methylome. While I expect significant correlations between DNAm age and abundance measures of some blood cell types (that are known to change with age), these correlations do not reflect a direct causal effect of cell type abundance on DNAm age but rather a confounding effect due to chronological age. This conclusion is also corroborated by the finding that DNAm age is highly related to chronological age in other types of cells - for example, glial cells and neurons (Figure 1H) and various brain regions (Additional file 5).

### DNAm age and progeria

DNAm age can be used to study whether cells from patients with accelerated aging diseases such as progeria (including Werner progeroid syndrome, Hutchinson-Gilford progeria) truly look old at an epigenetic level. I find that progeria disease status is not related to DNAm-based age acceleration in Epstein-Barr virus-transformed B cells (Figure 2T).

### Tissues where DNAm age is poorly calibrated

DNAm age is poorly calibrated (that is, leads to a high error) in breast tissue (Figure 2H), uterine endometrium (Figure 2S), dermal fibroblasts (Figure 1M), skeletal muscle tissue (Figure 2P), and heart tissue (Figures 1Q and 2L). I can only speculate on the biological reasons that could explain the poor calibration. The high error in breast tissue (Additional file 6) may reflect hormonal effects or cancer field effects in this normal adjacent tissue from cancer samples. Note that the lowest error (8.9 years) in breast tissue is observed in normal breast tissue, that is, in samples from women without cancer (training data set 14; Additional file 6). The menstrual cycle and concomitant increases in cell proliferation may explain the high error in uterine endometrium. Myosatellite cells may effectively rejuvenate the DNAm age of skeletal muscle tissue. Similarly, the recruitment of stem cells into cardiomyocytes for new cardiac muscle formation could explain why human heart tissue tends to have a low DNAm age. Carefully designed studies will be needed to test these hypotheses.

### The age correlation in a data set is determined by the standard deviation of age

In the following, I describe non-biological reasons that affect the accuracy (age correlation) of the age predictor. To address how well the age predictor works in individual data sets, I used two different approaches. First, I applied the age predictor to individual data sets (see columns 'Cor(Age, DNAmAge)’ and 'Median Error(Age, DNAmAge)’ in Additional file 1). An obvious limitation of this approach is that it leads to biased results in the training data sets.

The second approach, referred to as leave-one-data-set-out cross-validation (LOOCV) analysis, leads to unbiased estimates of the predictive accuracy for each data set. As suggested by its name, this approach estimates the DNAm age for each data set (considered as test data set) separately by fitting a separate multi-tissue age predictor to the remaining (left out) data sets. The resulting unbiased estimates of predictive accuracy can be found in Additional file 1: columns 'Cor LOOCV’ and 'Error LOOCV’, respectively.

**Figure 3:**
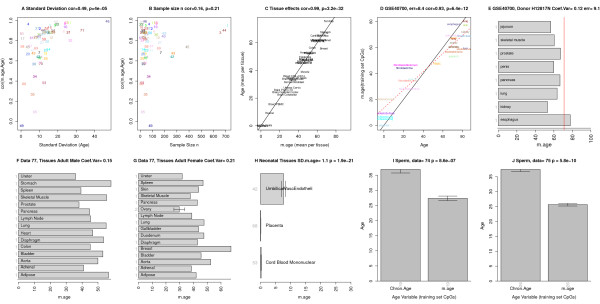
**Factors affecting the relation between age and DNAm age. (A-C)** Factors influencing prediction accuracy in the training and test sets. **(A)** The standard deviation of age (x-axis) has a strong relationship (cor = 0.49, *P* = 4E-5) with age correlation (y-axis). To arrive at an unbiased measure of prediction accuracy, I estimated the age correlation using a leave-one-data-set-out cross validation (LOOCV) analysis. Each point is labeled and colored according to the underlying data set (Additional file 1). **(B)** Sample size (x-axis) is not significantly correlated with the age correlation (y-axis). **(C)**  Mean DNAm age per tissue (x-axis) versus mean chronological age (y-axis). Points correspond to the human tissue data mentioned in Additional file 1. Breast tissue shows signs of accelerated aging. **(D,E)** The effect of tissue type on the age prediction in test data set 71 even for tissues that were not part of the training data (for example, esophagus, jejunum, penis). **(E)** The horizontal bars report the DNAm age (x-axis) of a single tissue from a single donor (H12817). Only one sample per tissue (grey axis numbers) was available. DNAm age has a low coefficient of variation (0.12). The red vertical line corresponds to the true chronological age. **(F-H)** DNAm age for various tissues from data set 77 but chronological age was not available. **(F,G)** A multi-tissue analysis of somatic adult tissue data from an adult male and an adult female, respectively. **(H)** Neonatal tissues tend to have low DNAm age. **(I,J)** The DNAm age of sperm is significantly lower than the chronological age of the respective sperm donors in data sets 74 and 75, respectively. Error bars represent one standard error.

Data sets differ greatly with respect to the median chronological age and the standard deviation, which is defined as the square root of the variance of age. Some data sets only involve samples with the same age (standard deviation = 0) while others involve both young and old subjects (Additional file 1). As expected, the standard deviation is significantly correlated (r = 0.49, *P* = 4E-5; Figure 3A) with the corresponding LOOCV estimate of the age correlation. In contrast, the sample size of the data set has no significant relationship with the age correlation (Figure 3B).

A host of technical artifacts could explain differences in predictive accuracy: for example, variations in sample processing, DNA extraction, DNA storage effects, batch effects, and chip effects.

### DNAm age of multiple tissues from the same subject

In the following, I will address whether solid tissues can be found whose DNAm age differs substantially from chronological age. As a first step, I compared the mean DNAm age per tissue with the corresponding mean chronological age. As expected, mean DNAm age per tissue is highly correlated (cor = 0.99; Figure 3C) with mean chronological age. But breast tissue shows evidence of significant age acceleration. The results of Figure 3C should be interpreted with caution because the analysis included training data sets and involved tissue samples from different subjects.

A more interesting analysis is to compare the DNAm ages of tissues collected from the same subjects. DNAm age does not change significantly across different brain regions (temporal cortex, pons, frontal cortex, cerebellum) from the same subjects (Additional file 5K,L). I could only find three human subjects from whom many tissues had been profiled (Figure 3E-G). Although the limited sample sizes per tissue (mostly one sample per tissue per subject) did not allow for rigorous testing, these data can be used to estimate the coefficient of variation of DNAm age (that is, the standard deviation divided by the mean). Note that the coefficient of variations for the first and second adult male are relatively low (0.12 and 0.15 in Figure 3E,F) even though the analysis involved several tissues that were not part of the training data - for example, jejunum, penis, pancreas, esophagus, spleen, pancreas, lymph node, diaphragm. The coefficient of variation in the adult female (Figure 3G) is relatively high (0.21), which reflects the fact that her breast tissue shows signs of substantial age acceleration (congruent with the previous results from Figure 3C).

It remains to be seen how well DNAm age performs in tissues and DNA sources that were not represented in the training data set. Figure 3D,E suggest that it also performs well in several other human tissues. As expected, I did not find a significant age correlation in sperm. The DNAm age of sperm is significantly lower than the chronological age of the donor (Figure 3I, J).

### DNAm age is applicable to chimpanzees

**Figure 4:**
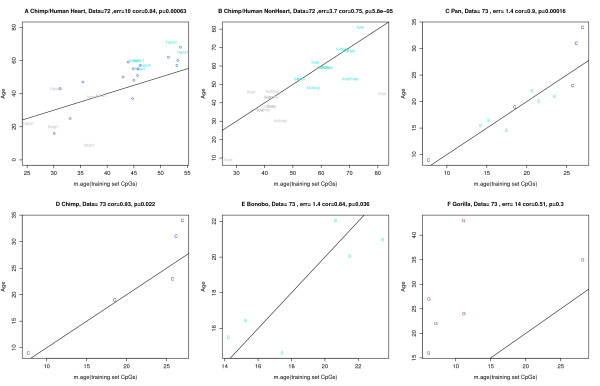
**Studying the conservation of DNAm age in tissues from great apes.** Analysis of two independent data sets involving tissues from great apes. **(A,B)** Results for data set 72 [27]. A high age correlation (cor = 0.84, error = 10 years) can be observed when studying both chimpanzee heart (colored grey) and human heart tissue (colored turquoise) samples. To facilitate a comparison, I also added the heart tissue data from data set 25 (blue circles). **(B)** DNAm age is closely related to chronological age (cor = 0.75, error = 3.7) across kidney and liver samples from humans (turquoise) and chimpanzees (grey). **(C-F)** Results for ape blood samples from data set 73. **(C)** Highly accurate results (cor = 0.9, error = 1.4) can be observed for blood samples from common chimpanzees (*Pan troglodytes*; labeled C, colored blue) and bonobos (*Pan paniscus*; labeled B, colored turquoise). **(D)** Results for common chimpanzees only. **(E)** Results for bonobos only. **(F)** Results for gorillas.

It is important to study whether there are inter-primate differences when it comes to DNAm age. These studies may not only help in identifying model organisms for rejuvenating interventions but might explain differences in primate longevity. While future studies could account for sequence differences, it is straightforward to apply the DNAm age estimation algorithm to Illumina DNA methylation data sets 72 [27] and 73 [28]. Strikingly, the DNAm age of heart, liver, and kidney tissue from chimpanzees (*Pan troglodytes*) is aligned with that of the corresponding human tissues (Figure 4A,B). Further, the DNAm age of blood samples from two extant hominid species of the genus *Pan* (commonly referred to as chimpanzee) is highly correlated with chronological age (Figure 4C). While DNAm age is applicable to chimpanzees, its performance appears to be diminished in gorillas (Figure 4F), which may reflect the larger evolutionary distance.

### DNAm age of induced pluripotent stem cells and stem cells

**Figure 5:**
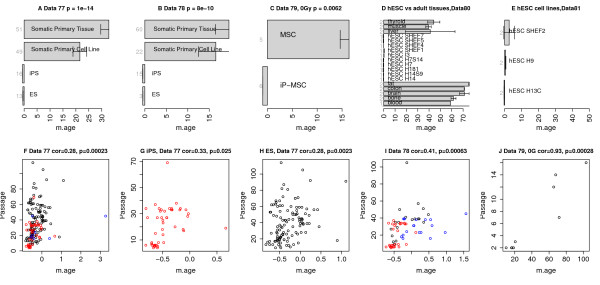
**Induced pluripotent stem cells, embryonic stem cells and cell passaging. (A-C)** Induced pluripotent stem (iPS) cells have a lower DNAm age than corresponding primary cells in **(A)** data set 77 (Kruskal Wallis *P*-value 1E-14), **(B)** data set 78 (*P* = 8E-10), and **(C)** data set 79 (*P* = 0.0062). **(A,B)** There is no significant difference in DNAm age between ES cells and iPS cells (both restricted to cell passage numbers less than 15) in data sets 77 and 78, respectively. **(D,E)** DNAm age of human ES cell lines and adult tissues in data sets 80 and 81, respectively. **(F**-**J)** Cell passage number (y-axis) is significantly correlated with DNAm age (x-axis). **(F)** Cell passage number (y-axis) versus DNAm age in data set 77. Points are colored by cell type (black for ES cells, red for iPS cells, blue for somatic cells). **(G,H)** Analogous results for iPS cells (cor = 0.33, *P* = 0.025) and embryonic stem cells (cor = 0.28, *P* = 0.0023) from data set 77. **(I,J)** Validation of these findings in two independent data sets, 78 and 79, respectively. Panel **(J)** involves only stem cells. Panels **(A-C)** involve cells that had undergone fewer than 15 cell passages. Panels **(C,J)** are restricted to cells that were not irradiated. The bar plots show the mean value ±1 standard error.

The billions of cells within an individual can be organized by genealogy into a single somatic cell tree that starts from the zygote and ends with differentiated cells. Cells at the root of this tree should be young. This is indeed the case: embryonic stem cells have a DNAm age close to zero in five different data sets (Figure 5). iPS cells are a type of pluripotent stem cell artificially derived from a non-pluripotent cell (typically an adult somatic cell) by inducing a set of specific genes. Since iPS cells are similar to embryonic stem (ES) cells, I hypothesized that the DNAm age of iPS cells should be significantly younger than that of corresponding primary cells. I confirm this hypothesis in three independent data sets (Figures 5A-C). No significant difference in DNAm age could be detected between ES cells and iPS cells (Figure 5A,B).

### Effect of cell passaging on DNAm age

Most cells lose their proliferation and differentiation potential after a limited number of cell divisions (Hayflick limit). I hypothesized that cell passaging (also known as splitting cells) increases DNAm age. I confirmed this hypothesis in three independent data sets (data sets 77, 78, and 79) as shown in Figure 5F-J. A significant correlation between cell passage number and DNAm age can also be observed when restricting the analysis to iPS cells (Figure 5G) or when restricting the analysis to ES cells (Figure 5H,J).

### Comparing the multi-tissue predictor with other age predictors

As shown in Additional file 2, the proposed multi-tissue predictor of age outperforms predictors described in other articles [21,23]. While further gains in accuracy can perhaps be achieved by focusing on a single tissue and considering more CpGs, the major strength of the proposed multi-tissue age predictor lies in its wide applicability: for most tissues it will not require any adjustments or offsets. I briefly mention that a 'shrunken’ version of the multi-tissue predictor (Additional files 2 and 3), based on 110 CpGs (selected from the 353 clock CpGs) is highly accurate in the training data (cor = 0.95, error = 4 years) and test data (cor = 0.95, error = 4.2 years).

### What is known about the 353 clock CpGs?

An Ingenuity Pathway analysis of the genes that co-locate with the 353 clock CpGs shows significant enrichment for cell death/survival, cellular growth/proliferation, organismal/tissue development, and cancer (Additional file 7).

The 353 clock CpGs can be divided into two sets according to their correlation with age. The 193 positively and 160 negatively correlated CpGs get hypermethylated and hypomethylated with age, respectively. Using DNA methylation data measured across many different adult and fetal tissues, I study the relationship between tissue variance and age effects (Additional file 8). While the DNA methylation levels of the 193 positively related CpGs vary less across different tissues, those of the 160 negatively related CpGs vary more across tissues than the remaining CpGs on the Illumina 27K array. To estimate 'pure’ age effects, I used a meta analysis method that implicitly conditions on data set, that is, it removes the confounding effects due to data set and tissue type. The clock CpGs include those with the most significant meta analysis *P*-value for age irrespective of whether the meta analysis *P*-value was calculated using only training data sets or all data sets (Additional file 8E). While positively related markers do not show a significant relationship with CpG island status (Additional file 9F), negatively related markers tend to be over-represented in CpG shores (*P* = 9.3E-6; Additional file 9K).

Significant differences between positive and negative markers exist when it comes to Polycomb-group protein binding: positively related CpGs are over-represented near Polycomb-group target genes (reflecting results from [10,14]) while negative CpGs show no significant relationship (Additional file 9H-J,M-O).

### Chromatin state analysis

Chromatin state profiling has emerged as a powerful means of genome annotation and detection of regulatory activity. It provides a systematic means of detecting *cis*-regulatory elements (given the central role of chromatin in mediating regulatory signals and controlling DNA access) and can be used for characterizing non-coding portions of the genome, which contribute to cellular phenotypes [29]. While individual histone modifications are associated with regulator binding, transcriptional initiation, enhancer activity, combinations of chromatin modifications can provide even more precise insight into chromatin state [29]. Ernst *et al*. [29] distinguish six broad classes of chromatin states, referred to as promoter, enhancer, insulator, transcribed, repressed, and inactive states. Within them, active, weak and poised promoters (states 1 to 3) differ in expression levels, while strong and weak enhancers (states 4 to 7) differ in expression of proximal genes. The 193 positively related CpGs are more likely to be in poised promoters (chromatin state 3 regions; Additional file 9B) while the 160 negatively related CpGs are more likely to be in either weak promoters (chromatin state 2; Additional file 9D) or strong enhancers (chromatin state 4; Additional file 9E).

### Age acceleration is highly heritable

Several authors have found that DNA methylation levels are under genetic control [24,26,30-32]. Since many age-related diseases are heritable, it is interesting to study whether age acceleration (here defined as difference between DNAm age and chronological age) is heritable as well. I estimated the broad sense heritability of age acceleration using Falconer’s formula, H^2^ = 2(cor(MZ)-cor(DZ)), in two twin data sets that included both monozygotic (MZ) and dizygotic (DZ) twins.

As detailed in Additional file 10, the broad sense heritability of age acceleration is 100% in newborns (data set 50) and 39% in older subjects (data set 41), which suggests that non-genetic factors become more relevant later in life.

### Aging effects on gene expression (messenger RNA) levels

Since DNA methylation is an important epigenetic mechanism for regulating gene expression levels (messenger RNA abundance), it is natural to wonder how age-related DNAm changes relate to those observed in gene expression levels. As described in Additional file 11, I find very little overlap. Further, I do not find that age effects on DNAm levels affect genes known to be differentially expressed between naive CD8 T cells and CD8 memory cells (Additional file 11). These non-significant results reflect the fact that the relationship between DNAm levels and expression levels is complex [33,34].

### Age effects on individual CpGs

**Figure 6:**
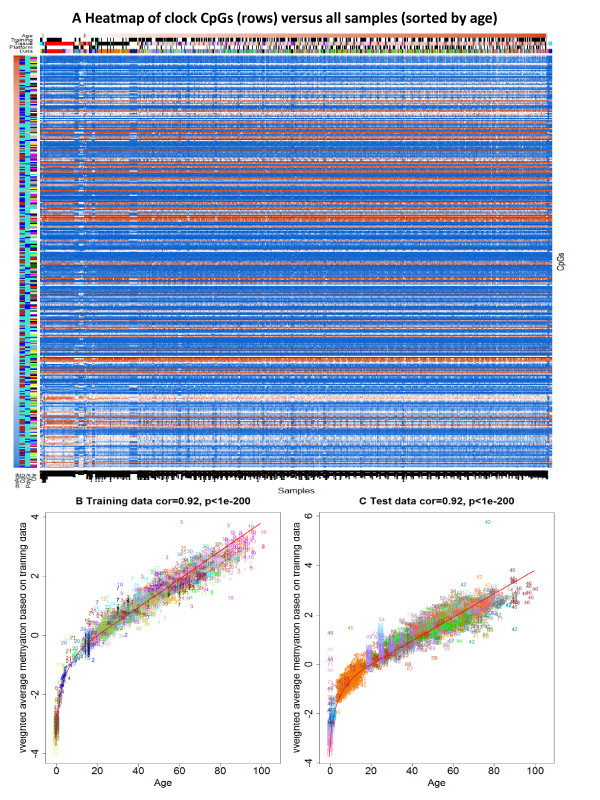
**Heat map of DNA methylation levels of the 353 CpGs across all samples. (A)** The heat map color-codes DNAm levels: blue and red for beta values close to zero and one, respectively. Note that DNA methylation levels only change very gradually with age. The 353 clock CpGs (rows) are sorted according to their age correlation. The first row color band, denoted 'corAge’, color-codes whether a CpG has a negative (blue) or positive (red) correlation with age. 'CpG’ indicates whether a CpG is located in a CpG island (turquoise), shore (brown), or outside of CpG islands. 'PolyGr’: blue for CpGs near a Polycomb group target gene. 'Chr’ color-codes chromosomes. The DNA methylation samples (columns) for which chronological age was available are sorted according to age, tissue, and data set. The column color bands visualize properties of the samples. 'Age’: white for age zero and dark brown for the maximum observed age of 101 years. 'Training’: black for training set samples. 'Tissue’ color codes tissue type. 'Platform’: black for Illumina 450K. Note that few data sets have a pronounced effect on the clock CpGs. The largest vertical band corresponds to the buccal epithelium samples from 15 year old subjects (data set 14, color-coded midnight blue in the column band 'Data’). **(B)** The weighted average of the 353 clock CpGs versus chronological age in the training data sets. The rate of change of the red curve can be interpreted as tick rate. Points are colored and labeled by data set. **(C)** Analogous results for the test data sets.

For each CpG, I report the median DNAm level in subjects aged younger than 35 years and in subjects older than 55 years (Additional file 3). The age-related change in beta values is typically small (the average absolute difference across the 353 CpGs is only 0.032). The weak age effect on individual clock CpGs can also be observed in the heat map that visualizes how the DNAm levels change across subjects (Figure 6A). The few vertical bands in the heat map suggest that the clock CpGs are relatively robust against tissue and data set effects.

### The changing ticking rate of the epigenetic clock

The linear combination of the 353 clock CpGs (resulting from the regression coefficients) varies greatly across ages as can be seen from Figure 6B,C. The red calibration curve (formula in Additional file 2) reveals a logarithmic dependence until adulthood that slows to a linear dependence later in life (Figure 6B). I interpret the rate of change (of this red curve) as the ticking rate of the epigenetic clock. Using this terminology, I find that organismal growth (and concomitant cell division) leads to a high ticking rate that slows down to a constant ticking rate (linear dependence) after adulthood.

### DNAm age does not measure mitotic age or cellular senescence

Since epigenetic somatic errors in somatic replications appear to be readily detected as age-related changes in methylation [35,36], it is a plausible hypothesis that DNAm age measures the number of somatic cell replications. In other words, that it measures mitotic age (which assigns a cell copy number to every cell) [35,37]. While DNAm age is correlated with cell passage number (Figure 5) and the clock ticking rate is highest during organismal growth (Figure 6B,C), it is clearly different from mitotic age since it tracks chronological age in non-proliferative tissue (for example, brain tissue) and assigns similar ages to both short and long lived blood cells (Additional file 4T).

Another plausible hypothesis is that DNAm age is a marker of cellular senescence. This turns out to be wrong as can be seen from the fact that DNAm age is highly related to chronological age in immortal, non-senescent cells - for example, immortalized B cells (Figure 2T). Further, DNAm age and cell passage number are highly correlated in ES cells (Figure 5H,J), which are also immortal [38].

### Model: DNAm age measures the work done by an epigenetic maintenance system

I propose that DNAm age measures the cumulative work done by a particular kind of epigenetic maintenance system (EMS), which helps maintain epigenetic stability. While epigenetic stability is related to genomic stability, I find it useful to distinguish these two concepts. If the EMS model of DNAm age is correct, then this particular kind of EMS appears to be inactive in the perfectly young ES cells. Maintenance methyltransferases are likely to play an important role. In physics, 'work’ is defined by the integral of power over time. Using this terminology, I hypothesize that the power (defined as rate of change of the energy spent by this EMS) corresponds to the tick rate of the epigenetic clock. This model would explain the high tick rate during organismal development since a high power is required to maintain epigenetic stability during this stressful time. At the end of development, a constant amount of power is sufficient to maintain stability leading to a constant tick rate.

If this EMS model of DNAm age is correct, then DNAm age should be accelerated by many perturbations that affect epigenetic stability. Further, age acceleration should have some beneficial effects given the protective role of the EMS. In particular, the EMS model of DNAm age entails the following testable predictions. First, cancer tissue should show signs of accelerated age, reflecting the protective actions of the EMS. Second, many mitogens, genomic aberrations, and oncogenes, which trigger the response of the EMS, should be associated with accelerated DNAm age. Third, high age acceleration of cancer tissue should be associated with fewer somatic mutations given the protective role of the EMS. Fourth, mutations in TP53 should be associated with a lower age acceleration of cancer tissue if one further assumes that p53 signaling helps trigger the EMS.

All of these model predictions turn out to be true as will be shown in the following cancer applications.

### DNAm age of cancer tissue versus tumor morphology

I assembled a large collection of cancer data sets composed of n = 5,826 cancer samples from 32 individual cancer data sets (Additional file 12). Details on the cancer data sets can be found in Additional file 2. While some cancer tissues show relatively large correlations between DNAm age and patient age, the correlation between DNAm age and chronological age tends to be weak (cor = 0.15, *P* = 1.9E-29; Additional file 13A). Each cancer/affected tissue shows evidence of significant age acceleration with an average age acceleration of 36.2 years (Additional file 13B). Tumor morphology (grade and stage) has only a weak relationship with age acceleration in most cancers: only 4 out of 33 hypothesis tests led to a nominally (*P* < 0.05) significant result (Additional file 14). Only the negative correlation between stage and age acceleration in thyroid cancer remains significant (uncorrected *P* = 8.7E-9; Additional file 14Z) after applying a Bonferroni correction.

### Cancer tissues with high age acceleration exhibit fewer somatic mutations

**Figure 7:**
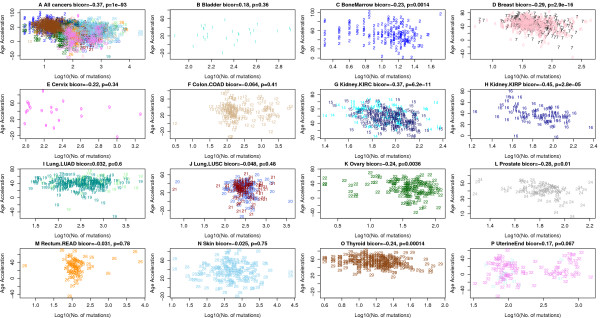
**Age acceleration versus number of somatic mutations in the TCGA data.** Mutation data from TCGA were used to count the number of mutations per cancer sample. **(A)** Age acceleration versus (log transformed) mutation count per sample across all cancers. Note that this analysis is confounded by cancer/tissue type. **(B**-**P)** A significant negative relationship between age acceleration and number of somatic mutations can be observed in the following seven affected tissues/cancers: **(C)** bone marrow (AML), **(D)** breast carcinoma (BRCA), **(G)** kidney (KIRC), **(H)** kidney (KIRP), **(K)** ovarian cancer (OVAR), **(L)** prostate (PRAD), and **(O)** thyroid (THCA). No significant relationship could be found in the following six cancer types: **(F)** colon carcinoma (COAD), **(I)** lung adenocarcinoma (LUAD), **(J)** lung squamous cell carcinoma (LUSC), **(P)** uterine endometrioid, **(M)** rectal cancer (READ), **(N)** skin. Due to the low sample size, the results are inconclusive for **(B)** bladder cancer and **(E)** cervical cancer. Each point corresponds to a DNA methylation sample (cancer sample from a human subject) analogous to Additional file 12. The x-axis reports the log transformed (base 10) number of mutations observed per sample. The figure titles report the biweight midcorrelation, which is a robust measure of correlation.

Strikingly, the number of mutations per cancer sample tends to be inversely correlated with age acceleration (Figure 7), which may reflect that DNAm age acceleration results from processes that promote genome stability. Specifically, a significant negative relationship between age acceleration and the number of somatic mutations can be observed in the following seven affected tissues/cancers: bone marrow (AML data from TCGA), breast carcinoma (BRCA data), kidney renal cell carcinoma (KIRC), kidney renal papillary cell carcinoma (KIRP), ovarian cancer (OVAR), prostate (PRAD), and thyroid (THCA). Similar results can also be observed in several breast cancer types (Additional file 15).

### TP53 mutations are associated with lower age acceleration

Additional file 16 presents the genes whose mutation has the strongest effect on age acceleration. Strikingly, *TP53* was among the top 2 most significant genes in 4 out of the 13 cancer data sets. Further, *TP53* mutation is associated with significantly lower age acceleration in five different cancer types (Additional file 17), including AML (*P* = 0.0023), breast cancer (*P* = 1.4E-5 and *P* = 3.7E-8), ovarian cancer (*P* = 0.03), and uterine corpus endometrioid (*P* = 0.00093). Further, marginally significant result can be observed in lung squamous cell carcinoma (Additional file 17) and colorectal cancer (*P* = 0.073, below). I could only find one cancer type (glioblastoma multiforme (GBM)) where mutations in *TP53* are associated with a nominally significant increased age acceleration (*P* = 0.02; Figure 5H). Overall, these results suggest that p53 signaling can trigger processes that accelerate DNAm age, which supports the EMS model of DNAm age.

### Somatic mutations in steroid receptors accelerate DNAm age in breast cancer

**Figure 8:**
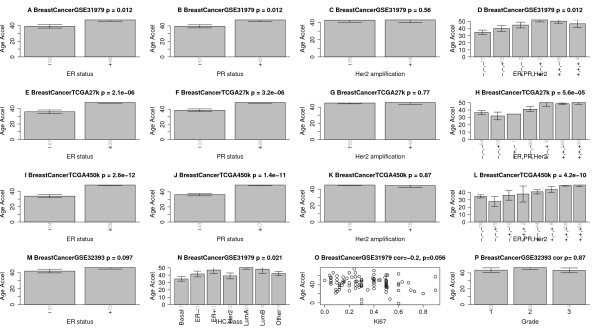
**Age acceleration in breast cancer.** Panels in the first column **(A,E,I,M)** show that estrogen receptor (ER)-positive breast cancer samples have increased age acceleration in four independent data sets. Panels in the second column **(B,F,J)** show the same result for progesterone receptor (PR)-positive cancers. Panels in the third column **(C,G,K)** show that HER2/neu amplification is not associated with age acceleration. Panels in the fourth column **(D,H,L)** show how combinations of these genomic aberrations affect age acceleration. **(N)** Age acceleration across the following breast cancer types: Basal-like, HER2-type, luminal A, luminal B, and healthy (normal) breast tissue. **(O)** Ki-67 expression versus age acceleration. **(P)** Tumor grade is not significantly related to age accelerations, reflecting results from Additional file 14. Vertical grey numbers on the x-axis report sample sizes. The figure titles report the data source (GSE identifier from Gene Expression Omnibus or TCGA), and the Kruskal Wallis test *P*-value (except for panels **(O,P)**, which report correlation test *P*-values). Error bars represent 1 standard error.

In the following, I show that DNAm age changes across different breast cancer types. Somatic mutations in steroid receptors have a pronounced effect on DNAm age in breast cancer samples: samples with a mutated estrogen receptor (ER) or mutated progesterone receptor (PR) exhibit a much higher age acceleration than ER- or PR- samples in four independent data sets (Figure 8). In contrast, HER2/neu amplification has no significant relationship with age acceleration. Age acceleration differs greatly across different breast cancer types (Figure 4N): luminal A tumors (typically ER+ or PR+, HER2-, low Ki67), show the highest positive age acceleration. Luminal B tumors (typically ER+ or PR+, HER2+ or HER2- with high Ki67) show a similar effect. The lowest age acceleration can be observed for basal-like tumors (often triple negative ER-, PR-, HER2-) and HER2 type tumors (typically HER2+, ER-, PR-).

### Proto-oncogenes affect DNAm age in colorectal cancer

**Figure 9:**
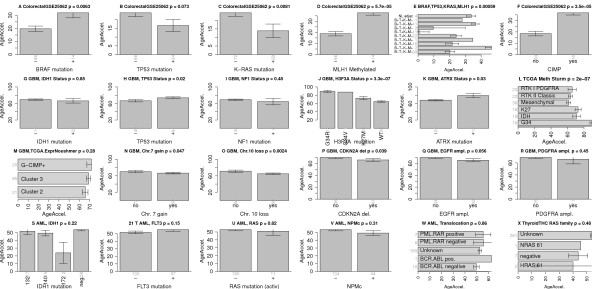
**Age acceleration in colorectal cancer, glioblastoma multiforme and acute myeloid leukemia. (A-F)** Results for colorectal cancer. Mean age acceleration (y-axis) in colorectal cancer versus mutation status (denoted by a plus sign) in **(A)***BRAF*, **(B)***TP53*, **(C)***K-RAS*. **(D)** Promoter hyper methylation of the mismatch repair gene *MLH1* (denoted by a plus sign) is significantly (*P* = 5.7E-5) associated with age acceleration. **(E)** Mean age acceleration across different patient groups defined by combinations of *BRAF*, *TP53*, *K-RAS*, *MLH1* status. The first bar reports the age acceleration in normal adjacent colorectal tissue from cancer patients but the sample size of 4 is rather low. **(F)** CpG island methylator phenotype is associated with age acceleration (*P* = 3.5E-5). **(G-R)** Results for various genomic abnormalities in glioblastoma multiforme. **(J)** A highly significant (*P* = 3.3E-7) relationship can be found between *H3F3A* mutations and age acceleration. Samples with a G34R mutation have the highest age acceleration. **(S-W)** Results for various genomic aberrations in acute myeloid leukemia. **(X)** Thyroid cancer age acceleration versus RAS family mutation status is inconclusive since mutation status was largely unknown. Error bars represent 1 standard error.

Colorectal cancer samples with a BRAF (V600E) mutation are associated with an increased age acceleration (Figure 9A) whereas samples with a K-RAS mutation have a decreased age acceleration (Figure 9C). Echoing previous results, TP53 mutations appear to be associated with decreased age acceleration (marginally significant *P* = 0.073; Figure 9B). Promoter hypermethylation of the mismatch repair gene *MLH1* leads to the most significant increase in age acceleration (*P* = 5.7E-5; Figure 9D), which supports the EMS model of DNAm age. The CpG island methylator phenotype, defined by exceptionally high cancer-specific DNA hypermethylation [39], is also significantly (*P* = 3.5E-5; Figure 9F) associated with age acceleration, which may reflect its association with *MLH1* hypermethylation and BRAF mutations.

### DNAm age in glioblastoma multiforme

In general, the CpG island methylator phenotype and age acceleration measure different properties as can be seen in GBM (Figure 9M).

Interestingly, age acceleration in GBM samples is highly significantly (*P* = 3.3E-7; Figure 9J) associated with certain mutations in *H3F3A*, which encodes the replication-independent histone variant H3.3. These mutations are single-nucleotide variants changing lysine 27 to methionine (K27M) or changing glycine 34 to arginine (G34R) [40]. The fact that GBMs with a G34R mutation in H3F3A have a much higher age acceleration than those with a K27M mutation (Figure 9J,L) makes sense since each *H3F3A* mutation defines an epigenetic subgroup of GBM with a distinct global methylation pattern and acts through a different set of genes [40]. Lysine 27 is a critical residue of histone 3 variants, and methylation at this position (H3K27me), which may be mimicked by the terminal CH3 of methionine substituted at this residue [40], is commonly associated with transcriptional repression [41] while H3K36 methylation or acetylation typically promotes gene transcription [42]. G34-mutant cells exhibit increased RNA polymerase II binding, and increased gene expression, most notably that of the oncogene *MYCN*[43]. Both *H3F3A* mutations are mutually exclusive with *IDH1* mutations, which characterize a third mutation-defined subgroup [44]. Age acceleration in GBM samples is also associated with the following genomic aberrations: *TP53* mutation, *ATRX* mutation, chromosome 7 gain, chromosome 10 loss, *CDKN2A* deletion, and *EGFR* amplification (Figure 9G-I). Reflecting these results for individual markers, age acceleration varies significantly (*P* = 2E-7; Figure 9L) across the GBM subtypes defined in [44].

### Acute myeloid leukemia

Mutations in *IDH1* (similar to the case of GBM), *FLT*, *RAS*, *NPMc*, and various well characterized translocations do not seem to relate to age acceleration in AML samples (Figure 9S-W).

### DNAm age of cancer cell lines

Using seven publicly available cell line data sets (Additional files 12 and 13), I was able to estimate the DNAm age of 59 different cancer cell lines (from bladder, breast, gliomas, head/neck, leukemia, and osteosarcoma). Across all cell lines, DNAm age does not have a significant correlation with the chronological age of the patient from whom the cancer cell line was derived (Additional file 18B). However, a marginally significant age correlation can be observed across osteosarcoma cell lines (cor = 0.41, *P* = 0.08; Additional file 18C). Overall, DNAm age acceleration varies greatly across the cancer lines lines (Additional files 18A and 19): the highest values can be observed for AML cell lines (KG1A, 182 years; HL-60, 177 years); the lowest values for head/neck squamous cell carcinoma cell line (UPCI SCC47, 6 years) and two breast cancer cell lines (SK-BR-3, 8 years; MDA-MB-468, 11 years). It will be interesting to test whether DNAm age relates to other characteristics of cancer cell lines.

## Conclusions

Through the generosity of hundreds of researchers, I was able to analyze an unprecedented collection of DNA methylation data from healthy tissues, cancer tissues, and cancer cell lines. The healthy tissue data allowed me to develop a multi-tissue predictor of age (mathematical details are provided in Additional file 2). An R software tutorial can be found in Additional file 20 (which requires Additional files 21, 22, 23, 24, 25, 26 and 27 as input). The basic approach is to form a weighted average of the 353 clock CpGs, which is then transformed to DNAm age using a calibration function. The calibration function reveals that the epigenetic clock has a high tick rate until adulthood, after which it slows to a constant tick rate.

I propose that DNAm age measures the cumulative work done by an epigenetic maintenance system. This EMS model of DNAm age leads to several testable model predictions that I validate using cancer data. But irrespective of the validity of the EMS model, the findings in cancer are interesting in their own right. While all cancer tissues exhibit signs of severe age acceleration, this is not necessarily the case for individual cancer cell lines. Overall, high age acceleration is associated with fewer somatic mutations in cancer tissue. Mutations in TP53 are associated with lower DNAm age. To provide a glimpse of how DNAm age can inform cancer research, I relate it to several widely used genomic aberrations in breast cancer, colorectal cancer, GBM, and AML.

DNAm age is arguably a promising marker for studying human development, aging, and cancer. It may become a useful surrogate marker for evaluating rejuvenation therapies. The most salient feature of DNAm age is its applicability to a broad spectrum of tissues and cell types. Since it allows one to contrast the ages of different tissues from the same subject, it can be used to identify tissues that show evidence of accelerated age due to disease (for example, cancer). It remains to be seen whether the DNAm age of easily accessible fluids/tissues (for example, saliva, buccal cells, blood, skin) can serve as a surrogate marker for inaccessible tissues (for example, brain, kidney, liver). It is noteworthy that DNAm age is applicable to chimpanzee tissues. Given the high heritability of age acceleration in young subjects, I expect that age acceleration will mainly be a relevant measure in older subjects. Using a relatively small data set, I did not find any evidence that a premature aging disease (progeria) is associated with accelerated DNAm age (Figure 2T). In Additional file 2, I discuss whether DNAm age fulfils the biomarker criteria developed by the American Federation for Aging Research.

Future research will need to clarify whether DNAm age is only a marker of aging or relates to an effector of aging. In conclusion, the epigenetic clock described here is likely to become a valuable addition to the telomere clock.

## Materials and methods

### Definition of DNAm age using a penalized regression model

Using the training data sets, I used a penalized regression model (implemented in the R package glmnet [45]) to regress a calibrated version of chronological age on 21,369 CpG probes that a) were present both on the Illumina 450K and 27K platform and b) had fewer than 10 missing values. The alpha parameter of glmnet was chosen to 0.5 (elastic net regression) and the lambda value was chosen using cross-validation on the training data (lambda = 0.0226). DNAm age was defined as predicted age. Mathematical details are provided in Additional file 2.

### Short description of the healthy tissue data sets

All data are publicly available (Additional file 1). Many data sets involve normal adjacent tissue from TCGA. Details on the individual data sets can be found in Additional file 2. To give credit to the many researchers who generated the data, I briefly mention relevant citations. Data sets 1 and 2 (whole blood samples from a Dutch population) were generated by Roel Ophoff and colleagues [14]. Data set 3 (whole blood) consists of whole blood samples from a recent large scale study of healthy individuals [24]. The authors used these and other data to estimate human aging rates and developed a highly accurate predictor of age based on blood data. Data set 4 consists of leukocyte samples from healthy male children from Children’s Hospital Boston [46]. Data set 5 consists of peripheral blood leukocyte samples [47]. Data set 6 consists of cord blood samples from newborns [30]. Data set 7 consists of cerebellum samples, which were provided by C Liu and C Chen (Gene Expression Omnibus (GEO) identifier GSE38873). Data sets 8, 9, 10, and 13 consist of cerebellum, frontal cortex, pons, and temporal cortex samples, respectively, obtained from the same subjects [48]. Data set 11 consists of prefrontal cortex samples from healthy controls [22]. Data set 12 consists of neuron and glial cell samples from [49]. Data set 14 consists of normal breast tissue samples [50]. Data set 15 consists of buccal cells from 109 15-year-old adolescents from a longitudinal study of child development [51]. Data set 16 consists of buccal cells from eight different subjects [15]. Data set 17 consists of buccal cells from monozygotic (MZ) and dizygotic (DZ) twin pairs from the Peri/postnatal Epigenetic Twins Study (PETS) cohort [52]. Data set 18 consists of cartilage (chondrocyte) samples from [53]. Data set 19 normal consists of adjacent colon tissue from TCGA. Data set 20 consists of colon mucosa samples from [54]. Data set 21 consists of dermal fibroblast samples from [21]. Data set 22 consists of epidermis samples from [55]. Data set 23 consists of gastric tissue samples from [56]. Data set 24 consists of head/neck normal adjacent tissue samples from TCGA (HNSC data). Data set 25 consists of heart tissue samples from [57]. Data set 26 consists of normal adjacent renal papillary tissue from TCGA (KIRP data). Data sets 27 consists of normal adjacent tissue from TCGA (KIRC data). Data set 28 consists of normal adjacent liver samples from [58]. Data set 29 consists of normal adjacent lung tissue from TCGA (LUSC data). Data set 30 consists of normal adjacent lung tissue samples from TCGA (LUAD data). Data set 31 is from TCGA (LUSC). Data set 32 consists of mesenchymal stromal cells isolated from bone marrow [59]. Data set 33 consists of placenta samples from mothers of monozygotic and dizygotic twins [60]. Data set 34 consists of prostate samples from [61]. Data set 35 consists of normal adjacent prostate tissue from TCGA (PRAD data). Data set 36 consists of male saliva samples from [62]. Data set 37 consists of male saliva samples from [23]. Data set 38 consists of stomach from TCGA (STAD data). Data set 39 consists of thyroid TCGA (THCA data). Data set 40 consists of whole blood from type 1 diabetics [10,63]. Data set 41 consists of whole blood from [15]. Data sets 42 and 43 consist of involve whole blood samples from women with ovarian cancer and healthy controls, respectively; these are the samples from the United Kingdom Ovarian Cancer Population Study [10,63]. Data set 44 consists of whole blood from [64]. Data set 45 consists of leukocytes from healthy children of the Simons Simple Collection [46]. Data set 46 consists of peripheral blood mononuclear cells from [65]. Data set 47 consists of peripheral blood mononuclear cells from [66]. Data set 48 consists of cord blood samples from newborns provided by N Turan and C Sapienza (GEO GSE36812). Data set 49 consists of cord blood mononuclear cells from [67]. Data set 50 consists of cord blood mononuclear cells from [60]. Data set 51 consists of CD4 T cells from infants [68]. Data set 52 consists of CD4+ T cells and CD14+ monocytes from [15]. Data set 53 consists of immortalized B cells and other cells from progeria, Werner syndrome patients, and controls [69]. Data sets 54 and 55 are brain samples from [70]. Data sets 56 and 57 consist of breast tissue from TCGA (27K and 450K platforms, respectively). Data set 58 consists of buccal cells from [71]. Data set 59 consists of colon from TCGA (COAD data). Data set 60 consists of fat (adipose) tissue from [72]. Data set 61 consists of human heart tissue from [27]. Data set 62 consists of kidney (normal adjacent) tissue from TCGA (KIRC). Data set 63 consists of liver (normal adjacent tissue) from TCGA (LIHC data). Data set 64 consists of lung from TCGA. Data set 65 consists of muscle tissue from [72]. Data set 66 consists of muscle tissue from [73]. Data set 67 consists of placenta samples from [74]. Data set 68 consists of female saliva samples [62]. Data set 69 consists of uterine cervix samples from [50,75]. Data set 70 consists of uterine endometrium (normal adjacent) tissue from TCGA (UCEC data). Data set 71 consists of various human tissues from the ENCODE/HAIB Project (GEO GSE40700). Data set 72 consists of chimpanzee and human tissues from [27]. Data set 73 consists of great ape blood samples from [28]. Data set 74 consists of sperm samples from [76]. Data set 75 consists of sperm samples from [77]. Data set 76 consists of vascular endothelial cells from human umbilical cords from [60]. Data sets 77 and 78 (special cell types) involve human embryonic stem cells, iPS cells, and somatic cell samples measured on the Illumina 27K array and Illumina 450K array, respectively [78]. Data set 79 consists of reprogrammed mesenchymal stromal cells from human bone marrow (iP-MSC), initial mesenchymal stromal cells, and embryonic stem cells [79]. Data set 80 consists of human ES cells and normal primary tissue from [80]. Data set 81 consists of human ES cells from [81]. Data set 82 consists of blood cell type data from [82].

### Description of the cancer data sets

An overview of the cancer tissue and cancer cell line data sets is provided in Additional file 12. More details can be found in Additional file 2.

All data are publicly available as can be seen from the column that reports GSE identifiers from the GEO database and other online resources. Most cancer data sets came from TCGA. Data set 3, GBM from [44]; data set 4, breast cancer from [83]; data set 5, breast cancer from [84]; data set 6, breast cancer from [50]; data set 10, colorectal cancer from [39]; data set 23, prostate cancer from [61]; data set 30, urothelial carcinoma from [85].

### DNA methylation profiling and normalization steps

All of the public Illumina DNA data were generated by following the standard protocol of Illumina methylation assays, which quantifies DNA methylation levels by the β value. A detailed description of the pre-processing and data normalization steps is provided in Additional file 2.

### Meta analysis for measuring pure age effects (irrespective of tissue type)

I used the *metaAnalysis* R function in the WGCNA R package [86] to measure pure age effects (Additional file 9) as detailed in Additional file 2.

### Analysis of variance for measuring tissue variation

To measure tissue effects in the training data (Additional file 8), I used analysis of variance (ANOVA) to calculate an F statistic as follows. First, a multivariate regression model was used to regress each CpG (dependent variable) on age and tissue type. The analysis adjusted for age since the different data sets have very different mean ages (Additional file 1). Next, ANOVA based on the multivariate regression model was used to calculate an F statistic, *F.tissueTraining*, for measuring the tissue effect in the training data. This F statistic measures the tissue effect after adjusting for age in the training data sets. I did not translate the F statistic into a corresponding *P*-value since the latter turned out to be extremely significant for most CpGs. Additional file 8D shows that *F.tissueTraining* is highly correlated with an independent measure of tissue variance (defined using adult somatic tissues from data set 77).

### Characterizing the CpGs using sequence properties

I studied occupancy counts for Polycomb-group target (PCGT) genes since they have an increased chance of becoming methylated with age compared to non-targets [10]. Toward this end, I used the occupancy counts of Suz12, Eed, and H3K27me3 published in [87]. To obtain the protein binding site occupancy throughout the entire nonrepeat portion of the human genome, Lee *et al*. [87] isolated DNA sequences bound to a particular protein of interest (for example, Polycomb-group protein SUZ12) by immunoprecipitating that protein (chromatin immunoprecipitation) and subsequently hybridizing the resulting fragments to a DNA microarray. More details on the chromatin state data from [29] can be found in Additional file 2.

## Abbreviations

AML: Acute myeloid leukemia; BLCA: Bladder urothelial carcinoma; CBMC: Cord blood mononuclear cell; CESC: Cervical squamous cell carcinoma and endocervical adenocarcinoma; COAD: Colon adenocarcinoma; EMS: Epigenetic maintenance system; ER: Estrogen receptor; ES: Embryonic stem; GBM: Glioblastoma multiforme; GEO: Gene Expression Omnibus; HNSC: Head/neck squamous cell carcinoma; HUVEC: Cell- human umbilical vascular endothelial cells; iPS: Induced pluripotent stem; KIRC: Kidney renal clear cell carcinoma; KIRP: Kidney renal papillary cell carcinoma; LIHC: Liver hepatocellular carcinoma; LOO: Leave one data set out; MSC: Mesenchymal stromal cell; PR: Progesterone receptor; PRAD: Prostate adenocarcinoma; READ: Rectum adenocarcinoma; SARC: Sarcoma; SCM: Skin cutaneous melanoma; TCGA: The Cancer Genome Atlas; THCA: Thyroid carcinoma; UCEC: Uterine corpus endometrioid carcinoma.

## Competing interests

The Regents of the University of California is the sole owner of a provisional patent application directed at this invention for which SH is a named inventor.

## Supplementary Material

Additional file 1 **DNA methylation data involving healthy (non-cancer) tissue.** The rows correspond to 82 publicly available Illumina data sets. Column 1 reports the data set number and corresponding color code. Other columns report the source of the DNA (for example, tissue), Illumina platform, sample size n, proportion of females, median age, age range (minimum and maximum age), relevant citation (first author and publication year), public availability (for example, GEO identifier). The column 'Data Use’ reports whether the data set was used as a training set, test set, or served another purpose. The table also reports the age correlation, Cor(Age, DNAmAge), median error, and median age acceleration for DNAm age. The last two columns of the table report the age correlation (Cor LOOCV) and median error (Error LOOCV) resulting from a leave-one-data-set-out cross-validation analysis. Click here for file

Additional file 2 **Materials and methods supplement.** This document has the following sections: Limitations; Description of the healthy tissue and cell line data sets; Criteria guiding the choice of the training sets; Description of the cancer data sets; DNAm profiling and pre-processing steps; Normalization methods for the DNA methylation data; Explicit details on the definition of DNAm age; Chromatin state data used for Additional file 9; Comparing the multi-tissue predictor with other age predictors; Meta analysis for finding age-related CpGs; Variation of age related CpGs across somatic tissues; Studying age effects using gene expression data; Meta-analysis applied to gene expression data; Names of the genes whose mutations are associated with age acceleration; Is DNAm age a biomarker of aging? Click here for file

Additional file 3 **Coefficient values for the DNAm age predictor.** This Excel file provides detailed information on the multi-tissue age predictor defined using the training set data. The multi-tissue age predictor uses 353 CpGs, of which 193 and 160 have positive and negative correlations with age, respectively. The table also represents the coefficient values for the shrunken age predictor that is based on a subset of 110 CpGs (a subset of the 353 CpGs). Although this information is sufficient for predicting age, I recommend using the R software tutorial since it implements the normalization method. The table reports a host of additional information for each CpG, including its variance, minimum value, maximum value, and median value across all training and test data. Further, it reports the median beta value in subjects aged younger than 35 years and in subjects older than 55 years. Click here for file

Additional file 4 **Age predictions in blood data sets. (A)** DNAm age has a high correlation with chronological age (y-axis) across all blood data sets. **(B-S)** Results for individual blood data sets. The negligible age correlation in panel 0) reflects very young subjects that were either zero or 0.75 years (9 months) old. (S) DNAm age in different cord blood data sets (x-axis). Bars report the mean DNAm age (±1 standard error). The mean DNAm age in data sets 6 and 50 is close to its expected value (zero) and it is not significantly different from zero in data set 48. (T) Mean DNAm age across whole blood, peripheral blood mononuclear cells, granulocytes as well as seven isolated cell populations (CD4+ T cells, CD8+ T cells, CD56+ natural killer cells, CD19+ B cells, CD14+ monocytes, neutrophils, and eosinophils) from healthy male subjects [82]. The red vertical line indicates the average age across subjects. No significant difference in DNAm age could be detected between these groups, but note the relatively small group sizes (indicated by the grey numbers on the y-axis). Click here for file

Additional file 5 **Age predictions in brain data sets. (A)** Scatter plot showing that DNAm age (defined using the training set CpGs) has a high correlation (cor = 0.96, error = 3.2 years) with chronological age (y-axis) across all training and test data sets. **(B-J)** Results in individual brain data sets. (G) The brain samples of data set 12 are composed of 58 glial cell (labeled G, blue color), 58 neuron cell (labeled N, red color), 20 bulk (labeled B, turquoise), and 9 mixed samples (labeled M, brown). **(K)** Comparison of mean DNAm ages (horizontal bars) across different brain regions from the same subjects [48] reveals no significant difference between temporal cortex, pons, frontal cortex, and cerebellum. Differing group sizes (grey numbers on the y-axis) reflect that some suspicious samples were removed in an unbiased fashion (Additional file 2). **(L)** Using data sets 54 and 55, I found no significant difference in DNAm age (x-axis) between cerebellum and occipital cortex from the same subjects [70]. Click here for file

Additional file 6 **Age predictions in breast data sets. (A)** DNAm age is highly correlated with age across all breast data sets, but the high error of 12 years reflects accelerated aging in normal adjacent breast cancer tissue (data sets 56, 57). **(B-D)** Relationship between DNAm age and chronological age in individual data sets. As expected, the lowest error (8.9 years) is observed in normal breast tissue (training data set 14, panel (B)). Click here for file

Additional file 7 **Ingenuity Pathway Analysis.** The document describes the results from applying Ingenuity Pathway Analysis to the 353 genes that are located near the 353 clock CpGs. Top biological function analysis implicated cell death/survival (74 genes, *P* = 1.1E-7) and cellular growth/development (71 genes, *P* = 3.7E-5). Significant overlap can be observed for the following disease-related gene sets: cancer (109 genes, *P* = 9.2E-5), endocrine system disorder (28, *P* = 2.6E-4), hereditary disorders (50 genes, 2.6E-4), and reproductive system disease (37 genes, *P* = 2.6E-4). Significant Ingenuity networks include a) hematological system development, tissue morphology, cell death and survival (*P* = E-37), b) cellular growth and proliferation, cell signaling, developmental disorder (*P* = E-37). Click here for file

Additional file 8 **Marginal analysis of CpGs.** The figure shows how individual CpGs (corresponding to points) relate to age and tissue variation. Red and blue points correspond the 193 positively and the 160 negatively related clock CpGs, respectively. **(A)** The variance across adult somatic tissues is highly correlated with variance across fetal somatic tissues, which illustrates that it is robustly defined. Note that data set 77 [78] was not used for defining DNAm age. **(B,C)** Average variance of DNAm levels across adult and fetal somatic tissues, respectively. The blue and red bars correspond to groups of positively and negatively related clock CpGs, respectively. **(D)** Tissue variance across the training data (F statistic from ANOVA) is highly correlated (cor = 0.73) with tissue variance across adult somatic tissues (data set 77), which illustrates that tissue variance is robustly defined. **(E)** Pure (unconfounded) age effects in the training data (x-axis) relate to those in all data sets (y-axis). To estimate pure age effects, I used a meta-analysis method that implicitly conditions on data set (Materials and methods; Additional file 2). The logarithm (base 10) of the meta-analysis *P*-value was multiplied by -1 or 1 so that high positive (negative) values indicate that the CpG is positively (negatively) correlated with age. The high correlation illustrates that little information is lost by focusing on the training data. Further, note that the most significantly positively (red dots) and negatively related CpGs (blue dots) are used in the epigenetic clock. **(F)** Tissue variance in the training data (y-axis) versus the signed logarithm of the meta-analysis *P*-value in the training data (x-axis). Click here for file

Additional file 9 **Characterizing the clock CpGs using DNA sequence properties.** Figure titles are preceded by ' + ’ or '-’ if they report properties of positively related or negatively related clock CpGs, respectively. Panels in the first row **(A-E)** relate the clock CpGs to chromatin state annotation provided in [29]. The y-axis reports the mean number of cell lines (out of 9 cell lines) for which the CpGs were in the chromatin state mentioned in the title. (A) The bar plots shows that the 193 positively related CpGs were significantly (*P* = 1.6E-6) less likely to be in chromatin state 1 (active promoters) than the other 21k CpGs, which is not the case for the 160 negatively related CpGs (C). (B) Positively related CpGs were more likely to be in chromatin state 3 regions (poised promoters). (D) Negatively related CpGs were more likely to be in chromatin states 2 (weak promoters). (E) Negatively related CpG are often located chromatin state 4 regions (strong enhancers). (F) No significant relationship with CpG island status can be observed for the positively related CpGs. (K) Negatively related CpGs are significantly over-represented in shores. (G) Positively related CpGs were outside of RNApol2 bound regions (annotation from [87]). This is not the case for negatively related CpGs (L). (H-J) Positively related CpGs are over-represented near Polycomb-group target genes, that is, in regions with high occupancy of Suz12 (*P* = 7.1E-6, H), EED (*P* = 0.0030, I), and H3K27m3 (*P* = 0.0048, J). This is not the case for the negatively related CpGs (M-O). Click here for file

Additional file 10 **Estimating the heritability of age acceleration.** Two twin data sets (data sets 41 and 50) are used to estimate the broad sense heritability of accelerated age (defined as difference between DNAm age and chronological age). **(A,E)** Age histograms for data set 41 (median age 63 years, all females) and data set 50 (composed of newborns), respectively. **(B,F)** All twins irrespective of zygosity. Each point corresponds to a twin pair and is colored red if the twins are monozygotic. Age acceleration of the first twin (randomly chosen) versus that in the second twin, respectively. (C,G) Monozygotic twins only. **(D,H)** Dizygotic twins only. The high correlations in monozygotic twins (cor = 0.4 for data set 41 and cor = 0.77 for data set 50) contrast sharply with those observed for dizygotic twins (cor = 0.20 and cor = -0.21). Click here for file

Additional file 11 **Aging effects in gene expression (mRNA) and DNAm data.** Due to space limitations, I can only report results for the direct approach of matching each individual CpG to its corresponding gene symbol. Using publicly available gene expression data (Additional file 2), I do not find a significant relationship between age effects on messenger RNA levels and age effects on DNAm levels in **(A)** blood, **(C)** brain, **(E)** kidney, **(G)** muscle, and **(I)** CD8 T cells. For each data modality, I estimated 'pure’ age effect using a meta-analysis method that conditioned on data (as described in Additional file 2). The y-axis reports a signed logarithm (base 10) of the meta-analysis *P*-value, that is, a high positive (negative) value indicates that the gene expression level increases (decreases) with age. Gene expression data and CpG data were matched according to gene symbol as described in [88]. Each point in the scatter plots corresponds to a CpG (x-axis) and the corresponding gene symbol (y-axis). Genes corresponding to the positively related and negatively related clock CpGs are colored in red and blue, respectively. **(B,D,F,H,J,L)** Mean age effect (y-axis) across gene groups defined by their corresponding CpG. **(K,L)** Aging effects on DNAm levels (x-axis) do not affect genes known to be differentially expressed between naive CD8 T cells and CD8 memory cells. The y-axis reports the signed logarithm of the Student *t*-test *P*-value of differential expression. Click here for file

Additional file 12 **Description of cancer data sets.** The file describes 32 publicly available cancer tissue data sets and 7 cancer cell line data sets. Column 1 reports the data number and corresponding color code. Other columns report the affected tissue, Illumina platform, sample size n, proportion of females, median age, age range (minimum and maximum age), relevant citation (TCGA or first author with publication year), and public availability. None of these data sets were used in the construction of estimator of DNAm age. The table also reports the age correlation, cor(Age,DNAmage), median error, and median age acceleration. Click here for file

Additional file 13 **DNAm age versus chronological age in cancer.** Each point corresponds to a DNA methylation sample (cancer sample from a human subject). Points are colored and labeled according to the underlying cancer data sets as described in Additional file 12. **(A)** Across all cancer data sets, there is only a weak correlation (cor = 0.15, *P* = 1.9E-29) between DNAm age (x-axis) and chronological patient age (y-axis). The high error (40 years) reflects high age accelerations. **(B)** Each cancer/affected tissue shows evidence of significant age acceleration (y-axis) with an average age acceleration of 36.2 years. **(C-W)** Results for individual cancers/affected tissues. Several cancer tissues maintain moderately large age correlations (larger than 0.3), including brain (cor = 0.61) (E), thyroid (cor = 0.6) (U), kidney (cor = 0.45) (K,L), liver (cor = 0.42) (M), colorectal (cor = 0.37) (I), and breast (cor = 0.31) (F). Click here for file

Additional file 14 **Age acceleration versus tumor grade and stage.** Panels correspond to the cancer data sets described in Additional file 12. Nominally significant negative correlations between grade and age acceleration can be observed in ovarian serous cystadenocarcinoma (panel G; *P* = 0.032) and uterine corpus endometroids (panel J; *P* = 0.019). A nominally significant positive correlation between stage and age acceleration can be observed for colon adenocarcinoma (panel O; *P* = 0.021). Only the highly significant negative correlation between stage and age acceleration in thyroid cancer (panel Z; *P* = 8.7E-9) remains significant after adjusting for multiple comparisons. Since grade and stage are often considered as ordinal variables, correlation test *P*-values are reported in all panels except the last. (H) For prostate cancer, the x-axis reports the Gleason sum score. The last panel shows that mean age acceleration in acute myeloid leukemia is not significantly related to French American British (FAB) morphology but some groups (notably M6 and M7) are very small (rotated grey numbers). Click here for file

Additional file 15 **Age acceleration versus mutation count status in breast cancer.** Mutation count status (x-axis) was defined by assigning tumor samples to the high mutation count group if their number of somatic mutations was larger than 50. Other thresholds lead to similar results. **(A-L)** Findings for Illumina 27K (A-F) and 450K data (G-L). (A,G) The barplots show that mean age acceleration (y-axis) is lower in breast cancer samples with high mutation count (compared to those samples whose somatic mutation count is less than 50). This result can also be found in ER+ (B,H), ER- (C,I), PR + (D,J), PR- (E,K), and triple negative (F,L) breast cancer samples. Click here for file

Additional file 16 **Selected significant gene mutations versus age acceleration.** The TCGA data sets were stratified by cancer type and Illumina platform. Mean age acceleration (y-axis) versus mutation status (x-axis) for up to two of the most significant genes per data set. Note that age acceleration in bone marrow (AML) was most highly related to mutation in the following two genes: *U2AF1* and *TP53*. Age acceleration in the two breast cancer data sets was most highly related to mutations in *GATA3*, *TP53*, and *TTN*. For kidney renal cell carcinoma (KIRC): only AKAP9 was significant. Strikingly, *TP53* was among the top two most significant mutated genes in 4 out of 13 cancer data sets. More information on these genes is presented in Additional file 2. Click here for file

Additional file 17 **Effect of *TP53 *mutation on age acceleration.** Mutations in *TP53* are associated with significantly lower age acceleration in five cancers: including AML (*P* = 0.0023), breast cancer (*P* = 1.4E-5 and *P* = 3.7E-8), ovarian serous cystadenocarcinoma (*P* = 0.03) (I), and uterine corpus endometrioid (*P* = 0.00093). Marginally significant results could be observed in lung squamous cell carcinoma (*P* = 0.047 for the 27K data but insignificant results for the 450K data). Click here for file

Additional file 18 **DNAm age of cancer cell lines. (A)** A high variation of DNAm age (x-axis) can be observed across various cancer lines lines (y-axis). The DNAm age is reported in Additional file 19. **(B)** Across all cell lines, DNAm age (x-axis) does not have a significant correlation with the chronological age of the patient from whom the cancer cell line was derived. **(C)** Results for osteosarcoma cell lines. Click here for file

Additional file 19 **Cancer lines and DNAm age.** This Excel file reports the DNAm age and age acceleration for 59 cancer cell lines. Click here for file

Additional file 20 **R software tutorial.** This file contains an R software tutorial that describes how to estimate DNAm age for data set 55. Further, it shows how to relate two measures of age acceleration to autism disease status. The R tutorial requires Additional files 21, 22, 23, 24, 25, 26 and 27 as input. Click here for file

Additional file 21 **Probe annotation file for the Illumina 27K array.** This comma-delimited text file (.csv file) is needed for the R software tutorial. Click here for file

Additional file 22 **Additional probe annotation file for the R tutorial.** This comma-delimited text file (.csv file) is needed for the R software tutorial. Click here for file

Additional file 23 **Coefficient values of the age predictor.** This comma-delimited text file (.csv file) is needed for the R software tutorial. This file is very similar to Additional file 3 but rows appear in a different order. Click here for file

Additional file 24 **R code for normalizing the DNA methylation data.** This text file is needed for the R software tutorial. It contains R code for normalizing the DNA methylation data and adapts R functions described in [89]. Click here for file

Additional file 25 **This text file is needed for the R software tutorial.** It contains R code implementing analysis steps. Click here for file

Additional file 26 **Methylation data from data set 55.** This comma-delimited text file (.csv file) contains the DNA methylation data needed for the R software tutorial. Click here for file

Additional file 27 This comma-delimited text file (.csv file) contains the sample annotation data needed for the R software tutorial. Click here for file
