# Erratum to: DNA methylation age of human tissues and cell types

**DOI:** 10.1186/s13059-015-0649-6

**Published:** 2015-05-13

**Authors:** Steve Horvath

**Affiliations:** Human Genetics, David Geffen School of Medicine, University of California Los Angeles, Los Angeles, CA 90095 USA; Biostatistics, School of Public Health, University of California Los Angeles, Los Angeles, California 90095 USA

I recently described an epigenetic biomarker of aging based on DNA methylation (DNAm) levels [1]. Unfortunately, I made a software coding error in my analysis of the cancer data, but not of the non-cancer tissue data. The error effectively added an offset term to the age estimates. All of my results from [1] that involve non-cancerous tissue or cancer cell lines remain valid but I have to report some corrections for the cancer tissue data. In particular, I have to retract the statement that cancer is associated with an increased DNA methylation age (i.e. positive age acceleration) in most cancer types. In fact, while some cancer types show positive age acceleration, others exhibit negative age acceleration. I deeply regret this software coding error. The error arose from me using the wrong age calibration function for the cancer tissue data sets, which led to a systematic over-estimation of DNA methylation age (Figure 1).

**Figure 1:**
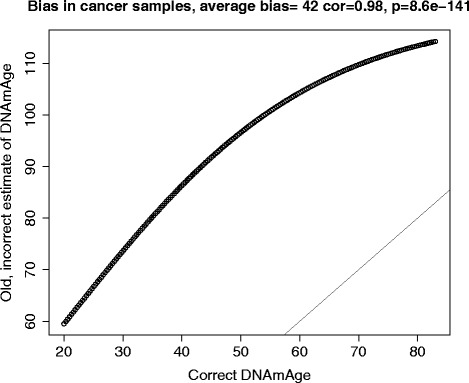
Evaluating the effect of the error on the DNAm age estimate in the cancer samples. The old, incorrect estimate of DNAm age (y-axis) versus the correct estimate (x-axis). Note that the two estimates are highly correlated (r = 0.98), which explains why most results are unaffected, but the old estimate is poorly calibrated, which leads to an average bias of 42 years. After using the correct estimate, I can no longer observe a positive age acceleration effect in cancer.

Fortunately, all of the other statements about cancer remain intact since the coding error effectively added an offset term to predicted age that changed little with chronological age (Figure 1). I am comforted by the fact that most of the reported results for cancer become even more significant, including the following. First, the results for cancer tissues are now more congruent with those obtained for cancer cell lines (which remain unchanged). Second, the age predictor leads to a much lower error in cancer tissues (now 16 years). Third, the results for TP53 become more significant, that is TP53 mutations are associated with lower age acceleration in colorectal cancer.

As a result of this error, the following Figures and Additional files are incorrect in the published paper, and correct versions are presented here:

- Figure seven in the original publication; Figure 2 here: Age acceleration versus number of somatic mutations in the TCGA data.

**Figure 2:**
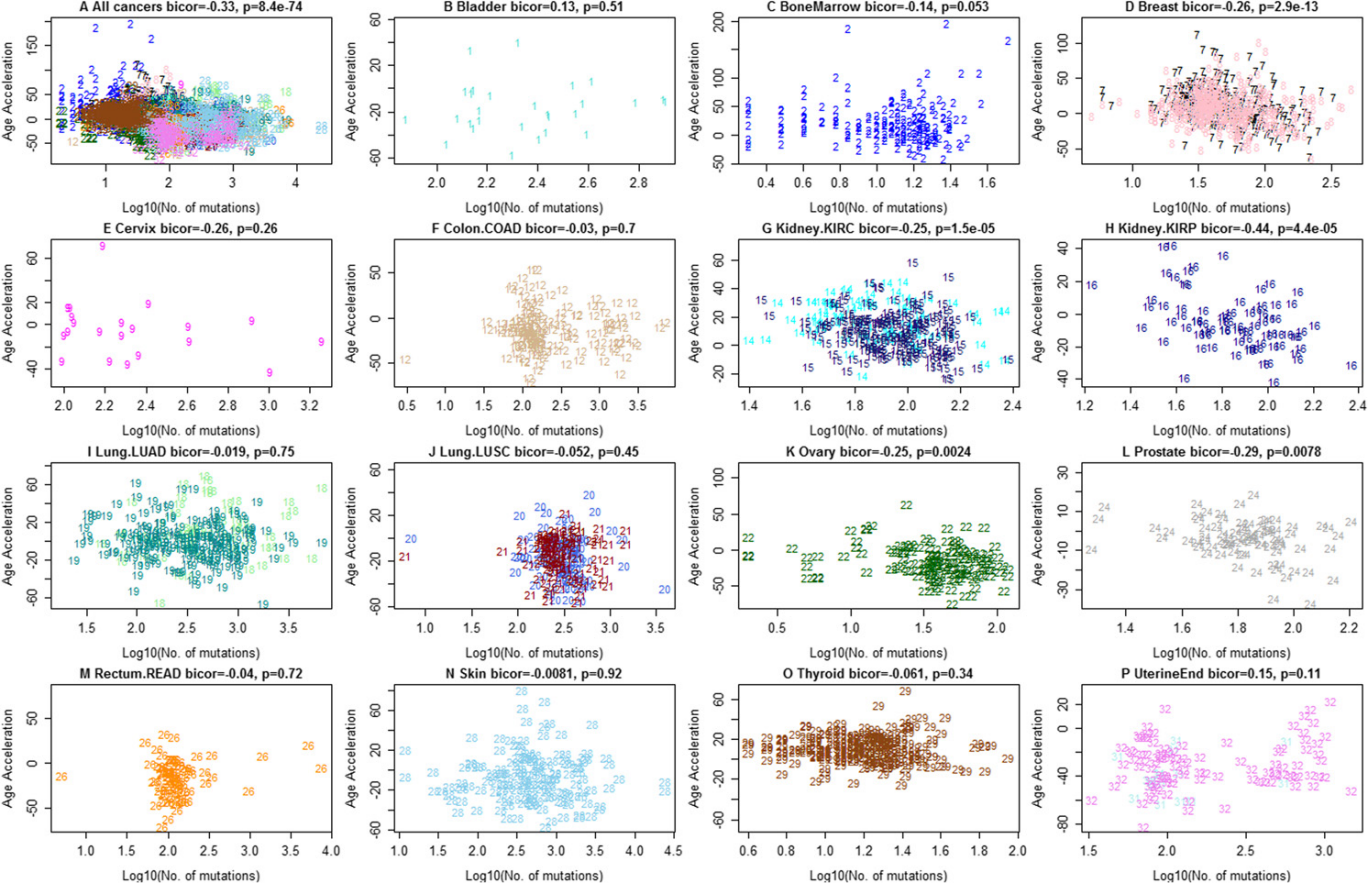
Age acceleration versus number of somatic mutations in the TCGA data. Mutation data from TCGA were used to count the number of mutations per cancer sample. **A)** Age acceleration versus (log transformed) mutation count per sample across all cancers. Note that this analysis is confounded by cancer/tissue type. **B-P)** A significant negative relationship between age acceleration and number of somatic mutations can be observed in the following seven affected tissues/cancers: **C)** bone marrow (AML), **D)** breast carcinoma (BRCA), **G)** kidney (KIRC), **H)** kidney (KIRP), **K)** ovarian cancer (OVAR), **L)** prostate (PRAD), and **O)** thyroid (THCA). No significant relationship could be found in the following six cancer types: **F)** colon carcinoma (COAD), **I)** lung adenocarcinoma (LUAD), **J)** lung squamous cell carcinoma (LUSC), **P)** uterine endometrioid, M) rectal cancer (READ), **N)** skin. Due to the low sample size, the results are inconclusive for **B)** bladder cancer and **E)** cervical cancer. Each point corresponds to a DNA methylation sample (cancer sample from a human subject) analogous to Additional file 1. The x-axis reports the log transformed (base 10) number of mutations observed per sample. The figure titles report the biweight midcorrelation, which is a robust measure of correlation.
- Figure eight in the original publication; Figure 3 here: Age acceleration in breast cancer.

**Figure 3:**
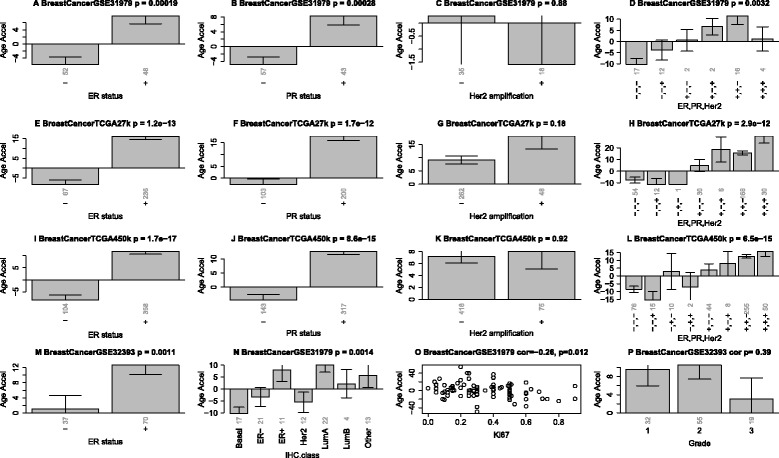
Age acceleration in breast cancer. Panels in the first column **(A,E,I,M)** show that estrogen receptor positive breast cancer samples have increased age acceleration in four independent data sets. Panels in the second column **(B,F,J)** show the same result for progesterone receptor positive cancers. Panels in the third columns **(C,G,K)** show that HER2/neu amplification is not associated with age acceleration. Panels in the fourth column **(D,H,L)** show how combinations of these genomic aberrations affect age acceleration. **N)** Age acceleration across the following breast cancer types: Basal-like, HER2-type, luminal A, luminal B, and healthy (normal) breast tissue. **O)** Ki-67 expression versus age acceleration. **P)** Tumor grade is not significantly related to age accelerations reflecting results from Additional file 3. Vertical grey numbers on the x-axis report sample sizes. The figure titles report the data source (GSE identifier from GEO or TCGA), and the Kruskal Wallis test p-value (except for panels O and P which report correlation test p-values).
- Figure nine in the original publication; Figure 4 here: Age acceleration in colorectal cancer, glioblastoma multiforme and acute myeloid leukemia.

**Figure 4:**
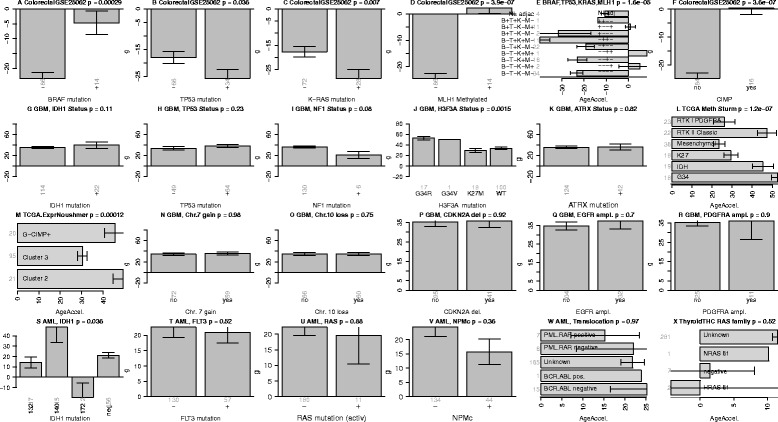
Age acceleration in colorectal cancer, GBM and AML. **A-F)** report results for colorectal cancer. Mean age acceleration (y-axis) in colorectal cancer versus mutation status (denoted by +) in **A)** BRAF, **B)** TP53, **C)** K-RAS. **D)** Promoter hyper methylation of the mismatch repair gene MLH1 (denoted +) is significantly associated with age acceleration. **E)** Mean age acceleration across different patient groups defined by combinations of BRAF, TP53, K-RAS, MLH1 status. The first bar reports the age acceleration in normal adjacent colorectal tissue from cancer patients but the sample size of 4 is rather low. **F)** CpG island methylator phenotype is associated with age acceleration. **G-R)** present results for various genomic abnormalities in glioblastoma multiforme. **J)** H3F3A mutations versus age acceleration. Samples with a G34R mutation have the highest age acceleration. Panels **S-W** (last row) show results for various genomic aberrations in acute myeloid leukemia. **X)** Thyroid cancer age acceleration versus RAS family mutation status is inconclusive since mutation status was largely unknown.
- Additional file twelve in the original publication; Additional file 1 here: Description of cancer data sets.
- Additional file thirteen in the original publication; Additional file 2 here: DNAm versus chronological age in cancer.
- Additional file fourteen in the original publication; Additional file 3 here: Age acceleration versus tumor grade and stage.
- Additional file fifteen in the original publication; Additional file 4 here: Age acceleration versus mutation count status in breast cancer.
- Additional file sixteen in the original publication; Additional file 5 here: Selected significant gene mutations versus age acceleration.
- Additional file seventeen in the original publication; Additional file 6 here: Effect of TP53 mutation on age acceleration.

Below, for sections of the original paper that are affected by the error, I explain how the corrected results are different from those that were reported.

## DNAm age of cancer tissue versus tumor morphology

In the original paper, I reported the correlation between DNAm age and chronological age as being 0.15 (P = 1.0×10^−29^). The correct correlation is 0.16 (p = 2.5×10^−33^; Additional file 2A). In addition, I reported that each cancer/affected tissue shows evidence of significant age acceleration. Instead, out of 20 cancer/affected tissues, only 6 exhibit positive age acceleration effects while others often show negative age acceleration effects, i.e. they appeared younger than expected (Figure 2B).

The following original statement remains unchanged: “Tumor morphology (grade and stage) has only a weak relationship with age acceleration in most cancers: only 4 out of 33 hypothesis tests led to a nominally (p < 0.05) significant result (Additional file 3)”.

But I have to retract the statement that only the negative correlation between stage and age acceleration in thyroid cancer remains significant after a applying a Bonferroni correction. It turns out that the uncorrected p-value of 0.0048 (Additional file 3Z) is not significant after multiplying it by 33.

### Cancer tissues with high age acceleration exhibit fewer somatic mutations

The original statement that the number of mutations per cancer sample tends to be inversely correlated with age acceleration (Figure 2A) remains unchanged.

But I have to retract the claim that one can observe a significant negative relationship between age acceleration and the number of somatic mutations in thyroid cancer (Figure 2O).

### TP53 mutations are associated with lower age acceleration

Additional file 5 presents the genes whose mutation has the strongest effect on age acceleration. The following original statement remains unchanged: "Strikingly, TP53 was among the top 2 most significant genes in 4 out of the 13 cancer data sets".

But I have to revise the following paragraph:

"Further, TP53 mutation is associated with significantly lower age acceleration in five different cancer types including AML (p = 0.0023), breast cancer (p = 1.4E-5 and p = 3.7E-8), ovarian cancer (p = 0.03), and uterine corpus endometrioid (p = 0.00093). Further, marginally significant result can be observed in lung squamous cell carcinoma and colorectal cancer (p = 0.073, below). I could only find one cancer type (GBM) where mutations in TP53 are associated with a nominally significant increased age acceleration (p = 0.02)".

as follows:

TP53 mutation is associated with significantly lower age acceleration in six data sets (Additional file 6) including AML (P = 0.0041), breast cancer (P = 7.8×10^−12^ and P = 1.4×10^−12^), ovarian cancer (P = 0.04), uterine corpus endometrioid (P = 0.0012), and colorectal cancer (P = 0.036, Figure 4B). Further, marginally significant result can be observed in lung squamous cell carcinoma (P = 0.088 Additional file 6G).

### Somatic mutations in steroid receptors accelerate DNAm age in breast cancer

The following original statement remains unchanged: “Age acceleration differs greatly across different breast cancer types (Figure 4N): Luminal A tumors (typically ER+ or PR+, HER2-, low Ki67), show the highest positive age acceleration”.

But I retract the statement that luminal B tumors (typically ER+ or PR+, HER2+ or HER2- with high Ki67) show a similar effect.

### Proto-oncogenes affect DNAm age in colorectal cancer

The p-value in the following statement "Echoing previous results, TP53 mutations appear to be associated with decreased age acceleration (p = 0.073)" needs to be revised to (p = 0.036, Figure 4B).

The p-value in the following statement "Promoter hypermethylation of the mismatch repair gene MLH1 leads to the most significant increase in age acceleration (P = 5.7×10^−5^)" needs to be revised to (p = 3.9×10^−7^, Figure 4D).

The p-value in the following statement "The CpG island methylator phenotype, defined by exceptionally high cancer-specific DNA hypermethylation, is also significantly (p = 3.5×10^−5^) associated with age acceleration" needs to be revised to (p = 3.6×10^−7^, Figure 4F).

### DNAm age in glioblastoma multiforme (GBM)

The p-value in the following statement "Interestingly, age acceleration in GBM samples is highly significantly (p = 3.3×10^−7^) associated with certain mutations in H3F3A" needs to be revised to (p = 0.0015, Figure 4J).

The p-value in the following statement "…age acceleration varies significantly (p = 2×10^−7^) across the GBM subtypes defined in (Sturm et al 2012)" needs to be revised to (p = 1.2×10^−7^, Figure 4L).

### Acute myeloid leukemia

The following statement remains unchanged: “Mutations in FLT3, RAS, NPMc, and various well characterized translocations do not seem to relate to age acceleration in AML samples”.

But I have to retract the claim that mutations in IDH1 do not relate to age acceleration. Rather, IDH1 mutations are nominally significantly related with age acceleration (p = 0.036, Figure 4S).

### DNAm age of cancer cell lines

My original results for cancer cell lines were not affected by the coding error;that is, they remain correct.

## Conclusions

My conclusion section remains largely unchanged. But I have to revise the following sentence: "While all cancer tissues exhibit signs of severe age acceleration, this is not necessarily the case for individual cancer cell lines".

It turns out that cancer types are similar to individual cancer cell lines. Some cancer types exhibit positive age acceleration effects (e.g. luminal breast cancer) while others exhibit negative age acceleration (e.g. basal breast cancer, Figure 3N).

## Additional files

Additional file 1:
**Description of cancer data sets.** The file describes 32 publicly available cancer tissue data sets and 7 cancer cell line data sets. Column 1 reports the data number and corresponding color code. Other columns report the affected tissue, Illumina platform, sample size n, proportion of females, median age, age range (minimum and maximum age), relevant citation (TCGA or first author with publication year), and public availability. None of these data sets were used in the construction of estimator of DNAm age. The table also reports the age correlation, cor(Age,DNAmage), median error, and median age acceleration. Additional file 2:
**DNAm age versus chronological age in cancer.** Each point corresponds to a DNA methylation sample (cancer sample from a human subject). Points are colored and labelled according to the underlying cancer data sets as described in Additional file 1. A) Across all cancer data sets, there is only a weak correlation (cor=0.16, p=2.5E-33) between DNAm age (x-axis) and chronological patient age (y-axis). B) Mean age acceleration (y-axis) versus cancer type. C-W) Results for individual cancers/affected tissues. Several cancer tissues maintain moderately large age correlations including E) brain, U) thyroid, K,L) kidney, M) liver, I) colorectal, and F) breast cancer. Additional file 3:
**Age acceleration versus tumor grade and stage.** Panels correspond to the cancer data sets described in Additional file 1. Nominally significant negative correlations between grade and age acceleration can be observed in ovarian serous cystadenocarcinoma (panel G) and uterine corpus endometroids (panel J). A nominally significant positive correlation between stage and age acceleration can be observed for colon adenocarcinoma (panel O). (Z) A significant negative correlation between stage and age acceleration can be observed in thyroid cancer. Since grade and stage are often considered as ordinal variables, correlation test p-values are reported in all panels except the last. H) For prostate cancer, the x-axis reports the Gleason sum score. The last panel shows that mean age acceleration in acute myeloid leukemia is not significantly related to French American British (FAB) morphology but some groups (notably M6 and M7) are very small (rotated grey numbers). Additional file 4:
**Age acceleration versus mutation count status in breast cancer.** Mutation count status (x-axis) was defined by assigning tumor samples to the high mutation count group if their number of somatic mutations was larger than 50. Other thresholds lead to similar results. A-F) and G-L) report findings for Illumina 27K and 450K data, respectively. A, G) The barplots show that mean age acceleration (y-axis) is lower in breast cancer samples with high mutation count (compared to those samples whose somatic mutation count is less than 50). This result can also be found in ER+ (panels B,H), ER- (C,I), PR+ (D,J), PR- (E,K), and triple negative (F,L) breast cancer samples. Additional file 5:
**Selected significant gene mutations versus age acceleration.** The TCGA data sets were stratified by cancer type and Illumina platform. Mean age acceleration (y-axis) versus mutation status (x-axis) for up to two of the most significant genes per data set. Note that age acceleration in bone marrow (AML) was most highly related to mutation in the following 2 genes: PHF6 and TP53. Age acceleration in the two breast cancer data was most highly related to mutations in GATA3, TP53, and TTN. Strikingly, TP53 was among the top 2 most significant mutated genes in four out of 13 cancer data sets. Additional file 6:
**Effect of TP53 mutation on age acceleration.** Mutations in TP53 are associated with significantly lower age acceleration in 5 cancers: including AML, breast cancer, ovarian serous cystadenocarcinoma, and uterine corpus endometrioid. Marginally significant results could be observed in lung squamous cell carcinoma (p=0.088 for the 27K data but not for the 450K data).
