# Supplementary material for: DNA methylation age of human tissues and cell types
Source: Genome Biol. 2013 Oct 21;14(10):R115. doi: 10.1186/gb-2013-14-10-r115 (PMC4015143; doi:10.1186/gb-2013-14-10-r115)
Supplement: Additional file 2 — Materials and methods supplement. This document has the following sections: Limitations; Description of the healthy tissue and cell line data sets; Criteria guiding the choice of the training sets; Description of the cancer data sets; DNAm profiling and pre-processing steps; Normalization methods for the DNA methylation data; Explicit details on the definition of DNAm age; Chromatin state data used for Additional file 9; Comparing the multi-tissue predictor with other age predictors; Meta analysis for finding age-related CpGs; Variation of age related CpGs across somatic tissues; Studying age effects using gene expression data; Meta-analysis applied to gene expression data; Names of the genes whose mutations are associated with age acceleration; Is DNAm age a biomarker of aging? [file gb-2013-14-10-r115-S2.docx]

**Additional File 2 "Materials and Methods"**

**for "DNA methylation age of human tissues and cell types"**

**Steve Horvath**

Contents

[Limitations 1](#_Toc368641091)

[Description of the healthy tissue and cell line data sets 2](#_Toc368641092)

[Criteria guiding the choice of the training sets 11](#_Toc368641093)

[Description of the cancer data sets 11](#_Toc368641094)

[DNAm profiling and pre-processing steps 12](#_Toc368641095)

[Normalization methods for the DNA methylation data 12](#_Toc368641096)

[Explicit details on the definition of DNAm age 13](#_Toc368641097)

[Chromatin state data used for Additional File 9 14](#_Toc368641098)

[Comparing the multi-tissue predictor with other age predictors 15](#_Toc368641099)

[Meta analysis for finding age-related CpGs 16](#_Toc368641100)

[Variation of age related CpGs across somatic tissues 17](#_Toc368641101)

[Studying age effects using gene expression data 18](#_Toc368641102)

[Meta analysis applied to gene expression data 20](#_Toc368641103)

[Names of the genes whose mutations are associated with age acceleration 21](#_Toc368641104)

[Is DNAm age a biomarker of aging? 21](#_Toc368641105)

[Online DNAm age calculator 21](#_Toc368641106)

[References 22](#_Toc368641107)

# Limitations

Here I highlight several limitations of my study. First, the number of samples per tissue varied greatly (Additional file 1). Second, data were measured using two different platforms: the Illumina Infinium27K and the more recent Infinium450K platform. Third, chronological age was confounded with data set: e.g. several data sets involved only cord blood samples (age zero) while others involved many elderly subjects. Given these limitations, the test set accuracy of the age predictor is remarkable (Figure 2).

The following reasons may explain the remarkable accuracy of the age predictor in the test data sets. First, measurements from Illumina DNA methylation arrays (Methods) are known to be less affected by normalization issues than those from gene expression (mRNA) arrays and even non-normalized beta-values (Methods) turn out to be highly correlated with corresponding measures found using pyrosequencing [[1-3](#_ENREF_1)]. Second, the penalized regression model automatically selected CpGs that are relatively robust since it was trained on data sets from different labs and platforms. Third, the large number of data sets helped average out spurious results and artifacts. Fourth, age has a profound effect on the DNAm levels of tens of thousands of CpGs as shown by many authors [[4-13](#_ENREF_4)].

The results of this article do not contradict previous studies that have noted age-related DNA methylation changes which occur in a tissue specific manner, e.g. [[14](#_ENREF_14), [15](#_ENREF_15)]. Instead, the results of this article demonstrate that one can use a couple of hundred CpGs for forming an age predictor that a) performs remarkably well across a broad spectrum of human tissues (Figure 1, Figure 2, and Figure 3) and b) the resulting DNAm age estimate is biologically meaningful.

# Description of the healthy tissue and cell line data sets

A table that provides an overview of the publicly available human DNAm data sets is provided in Additional File 1. The table reports the sample size, median age, age range, tissue source, and citation. All data are publicly available as can be seen from the column that reports GSE identifiers from the Gene Expression Omnibus (GEO) database and other online resources. In the following I provide a more detailed description of the individual data sets.

Data sets 1 and 2 (**whole blood samples from a Dutch population**) are comprised of schizophrenics and healthy control subjects measured on the Illumina 27K and 450K array platform, respectively. These data from Dr. Roel Ophoff's lab were formerly used to find co-methylation modules related to age [[13](#_ENREF_13)]. The current study has a different aim, namely the development of an age predictor based on methylation levels. Since schizophrenia status had a negligible effect on age relationships [[13](#_ENREF_13)], it was ignored in this analysis. Further, it turned out that schizophrenia status was not related to DNAm age. GEO identifier of the data is GSE41037.

Data set 3 (**whole blood**) consists of whole blood samples from a recent large scale study of healthy individuals [[16](#_ENREF_16)]. The authors used these data (and additional data) to estimate human aging rates and developed a highly accurate predictor of age based on blood data.

Data set 4 (**leukocytes from healthy male children** from Children's Hospital Boston) consists of 72 peripheral blood leukocyte samples from healthy males (mean age 5, range 1-16) [[17](#_ENREF_17)].

Data set 5 (**peripheral blood leukocytes**) from a DNAm study of Crohn's disease and ulcerative colitis [[18](#_ENREF_18)]. Illumina 450K were used on 48 samples of peripheral blood leukocyte (PBL) DNA from discordant MZ twin pairs (CD: 3; UC: 3) and treatment-naive pediatric cases of IBD (CD: 14; UC: 8), as well as controls (n = 14). I ignored disease status in the analysis. I did not find significant evidence that disease status affects DNAm age in this moderately sized data set.

Data set 6 (**cord blood from newborns**) is comprised of cord blood samples from 216 subjects (of age zero) [[19](#_ENREF_19)].

Data set 7 (**cerebellum**) is comprised of postmortem cerebellum brains. The data were provided by C. Liu and C. Chen (GEO identifier GSE38873).

Data set 8, 9, 10, 13 (**cerebellum, frontal cortex, pons, temporal cortex**) consist of brain tissue samples obtained from the same subjects whose mean age was 49 (range 15-101) [[20](#_ENREF_20)]. These subjects, who had donated their brains for research, were of non-Hispanic, Caucasian ethnicity, and none had a clinical history of neurological or cerebrovascular disease, or a diagnosis of cognitive impairment during life. Demographics, tissue source and cause of death for each subject are reported in [[20](#_ENREF_20)]. Unbiased removal of potential outliers (as described in the section on sample pre-processing) reduced the number of retained samples (Additional File 1).

Data set 11 (**prefrontal cortex from healthy controls**) consists of 108 samples (mean age 26, ranging from samples before birth up to age 84) [[21](#_ENREF_21)]. These post-mortem human brains from non-psychiatric controls were collected at the Clinical Brain Disorders Branch (National Institute of Mental Health). The DNAm data are publicly available from the webpage of the standalone package BrainCloudMethyl, which can be downloaded from the following URL:

http://braincloud.jhmi.edu/Methylation32/BrainCloudMethyl.htm

Data set 12 (**neuron and glial cells**) from [[22](#_ENREF_22)]. The authors developed a cell epigenotype specific model for the correction of brain cellular heterogeneity bias and applied it to study age, brain region and major depression. After performing fluorescence activated cell sorting (FACS) of neuronal nuclei in post mortem frontal cortex 58 samples(29 major depression and 29 matched control samples) followed by Illumina HM450 microarray based DNAm profiling, the authors characterized the extent of neuron and glia specific DNAm variation independent of disease status and identified significant cell type specific epigenetic variation at 51% of loci. I ignored disease status in the analysis. I found no evidence that disease status accelerated age in this data set.

Data set 14 (**breast**) consists of normal breast tissue from 23 females (mean age 48, range 19-75) downloaded from GEO [[23](#_ENREF_23)].

Data set 15 (**buccal cells**) involved 109 fifteen-year-old adolescents from a longitudinal study of child development [[24](#_ENREF_24)]. While the authors found that DNA derived from buccal epithelial cells showed differential methylation among adolescents whose parents reported high levels of stress during their children’s early lives, parental stress was ignored. All samples have the same chronological age (15 years).

Data set 16 (**buccal cells**) involved 8 different subjects. Rakyan et al (2010) confirmed that these buccal cell preparations contained very little, if any, leukocyte contamination, hence showing that the measured methylation profiles were predominantly from buccal cells [[25](#_ENREF_25)].

Data set 17 **(buccal cells**) from [[26](#_ENREF_26)]. The authors applied the Illumina 450K platform to buccal swabs from 10 monozygotic (MZ) and 5 dizygotic (DZ) twin pairs from the Peri/postnatal Epigenetic Twins Study (PETS) cohort. In this longitudinal study, DNAm profiles were generated at birth (age 0) and at age 1.5 years (18 months).

Data set 18 (**cartilage, chondrocytes**) from [[27](#_ENREF_27)]. The authors analyzed human articular chondrocytes from osteoarthritic patients and healthy cartilage samples. I did not find a relationship between disease status and accelerated DNAm age.

Data sets 19 (**colon, normal tissue**) consists of samples downloaded from TCGA data base measured on the Illumina 27K array.

Data set 20 (**colon mucosa**) from [[28](#_ENREF_28)]. Crohn's disease, ulcerative colitis, and normal colon mucosa samples were measured on the Illumina Infinium HumanMethylation450 BeadChip v1.1. Samples came from 9 Crohn's disease affected, 5 ulcerative colitis affected, and 10 normal individuals. I did not detect a significant relationship between disease status and DNAm age acceleration.

Data set 21 (**dermal fibroblasts**) consists of 14 female fibroblast samples (mean age 32, range 6-73). The samples came from different locations on the human body (5 abdomen, 2 arm, 2 breast, 3 ear, and 2 leg samples) [[2](#_ENREF_2)]. The single blepharoblast sample was removed from this data set since hierarchical clustering (based on the Euclidean distance, single linkage) indicated that it was an outlier.

Data set 22 (**epidermis**) came from a study that evaluated the epigenetic effects of aging and chronic sun exposure [[29](#_ENREF_29)]. I used the 10 epidermal samples collected using suction blistering.

Data set 23 (**gastric tissue**) from [[30](#_ENREF_30)]. The Illumina HumanMethylation27 BeadChip was used to obtain DNAm profiles across 27,578 CpGs in 203 gastric tumors and 94 matched non-malignant gastric samples. I focused on matched control samples.

Data set 24 (**head/neck normal adjacent tissues**) measured on the Illumina 450K platform from the TCGA data base (HNSC data).

Data set 25 (**heart tissue**) [[31](#_ENREF_31)]. The authors generated DNAm profiles from human left ventricular myocardium DNA in order to study alterations in cardiac DNAm in human dilated cardiomyopathy (DCM). There were n = 8 controls (patients after heart transplantation) and n = 9 patients with idiopathic DCM. I ignored disease status in the analysis. I could find no significant evidence that disease status affects DNAm age in this small data set.

Data sets 26 (**renal papillary, normal tissue**) consists of 44 samples (mean age 66) downloaded from TCGA data base (KIRP) measured on the Illumina 450K array.

Data sets 27 (**adjacent normal tissue, kidney measured on the Illumina 450K array**) from TCGA (Kidney Clear Cell Renal Carcinoma, KIRC).

Data set 28 (**liver**) consists of normal adjacent tissue samples from Taiwanese hepatocellular carcinoma subjects[[32](#_ENREF_32)]. The data were downloaded from GEO (GSE37988).

Data set 29 (**lung** **squamous cells** from normal adjacent tissue) consists of samples downloaded from TCGA data base (normal from LUSC) that were measured on the Illumina 27K array.

Data set 30 (**lung** normal adjacent lung tissue, Illumina 27K) from the The Cancer Genome Atlas (TCGA) data base (<http://tcga-data.nci.nih.gov/>), LUAD.

Data sets 31 (**lung squamous cells** from normal adjacent tissue measured on the Illumina 450K) from the TCGA data base (normal samples from LUSC).

Data set 32 (**mesenchymal stromal cells from bone marrow**) consists of 16 female samples (mean age 53, range 21-85) [[33](#_ENREF_33)]. The MSC from human bone marrow were either isolated from bone marrow aspirates or from the caput femoris upon hip fracture of elderly donors [[33](#_ENREF_33)]. Due to sample size constraints, cell passage status (reflecting short versus long term culture) was ignored.

Data set 33 (**placenta**) from mothers of monozygotic and dizygotic twins [[34](#_ENREF_34)]. Since placenta only develops during pregnancy, its chronological age was set to zero.

Data set 34 (**prostate**) consists of 69 normal prostate samples (mean age 61) [[35](#_ENREF_35)].

Data set 35 (**prostate,** normal adjacent tissue) measured on the Illumina 450K platform from the TCGA data base (PRAD data).

Data set 36 (**saliva** from alcoholic males) is from [[36](#_ENREF_36)] as data set 68, but involves 131 male samples (again with mean age 32, range 21-55). Thus, I split the original data by gender.

Data set 37 (**saliva from healthy men**) involved 69 healthy male samples (mean age 35, range 21-55). We used these twin pairs and triplets to develop a saliva based predictor of age [[3](#_ENREF_3)]. Since all twins were monozygotic, I could not use these data to estimate heritability with Falconer's formula.

Data sets 38 (**stomach** normal adjacent tissue measured on the Illumina 27K array) consists of 41 samples (mean age 69) downloaded from TCGA data base (STAD data).

Data set 39 (**thyroid**, normal adjacent tissue) measured on the Illumina 450K platform from the TCGA data base (THCA data).

Data set 40 (**WB from type 1 diabetics**) consists of samples from 191 subjects (mean age 44, range 24-74) [[12](#_ENREF_12), [37](#_ENREF_37)]. Since all subjects had type 1 diabetes, disease status was ignored. These data were downloaded from GEO (GSE20067).

Data set 41 (**WB from healthy females**) consists of 93 whole blood samples from women whose mean age was 63 (range 49-74) [[25](#_ENREF_25)]. The samples were collected from different healthy females (both twin pairs and singletons).

Data set 42 (**WB from postmenopausal women**) consists of 262 whole blood samples from women with ovarian cancer (mean age 66 , range 49-91). These are the cases from the UKOPS data (see data set 43). These samples were used since ovarian cancer did not have a global effect on blood methylation levels [[12](#_ENREF_12), [37](#_ENREF_37)].

Data set 43 (**WB from healthy postmenopausal women**) consists of 269 whole blood samples from women with a mean of 65 (range 52-78) [[12](#_ENREF_12), [37](#_ENREF_37)]. While the data come from the United Kingdom Ovarian Cancer Population Study (UKOPS), it is important to emphasize that the samples come from healthy age matched controls of ovarian cancer patients. The data were downloaded from GEO (GSE19711).

Data set 44 (**WB from rheumatoid arthritis**) from a differential DNAm study of rheumatoid arthritis [[38](#_ENREF_38)]. The authors found DNAm could serve as an intermediary of genetic risk in rheumatoid arthritis. I ignored disease status in the analysis. I did find that the whole blood of rheumatoid arthritis patients showed evidence of negative age acceleration compared to controls. While the large sample size led to a statistically significant (p=0.0049) finding, the effect size (age difference of 1.2 years) appears to be negligible.

Data set 45 (**leukocytes from healthy children** of the Simons Simple Collection) consists of peripheral blood leukocyte samples from 386 healthy (mostly male) subjects (mean age 10, range 3-17). These are healthy siblings of subjects with autism spectrum disorder (ASD) [[17](#_ENREF_17)].

Data set 46 (**peripheral blood mononuclear cells from newborns and nonagenarians**) [[39](#_ENREF_39)] can be downloaded from GEO GSE30870.

Data set 47 (**peripheral blood mononuclear cells**) collected from a community-based cohort stratified for early-life socioeconomic status [[40](#_ENREF_40)]. The data were downloaded from GEO (GSE37008). The authors found that psychosocial factors, such as perceived stress, and cortisol output were associated with DNAm patterns, as was early-life socioeconomic status. But none of these factors turned out to be related to DNAm age which justified that these covariates were ignored in this study.

Data set 48 (**cord blood samples from newborns**) comes from a study that related DNAm data to birth weight. Incidentally, DNAm age did not appear to be correlated with birth weight. No citation appears to be available for these data that were submitted to GEO (GSE36812) by N Turan and C Sapienza.

Data set 49 (**cord blood mononuclear cells**) comes from a study that investigated the effects of periconceptional maternal micronutrient supplementation on infant blood methylation patterns from offspring of Gambian women enrolled into a randomized, double blind controlled trial [[41](#_ENREF_41)]. No significant relationship between DNAm age and micronutrient supplementation status could be observed.

Data set 50 (**cord blood mononuclear cells**) is from monozygotic and dizygotic twins [[34](#_ENREF_34)] but twin status was ignored in our analysis.

Data set 51 (**CD4 T** cells from infants) consisted of sorted CD4+ T cell samples. The authors used the data to investigate the dynamics and relationship between DNAm and gene expression during early T-cell development[[42](#_ENREF_42)]. The mononuclear cells were collected from 24 infants at birth (n=12) and resampled at 12 months (n=12). CD4+ cells were purified and the DNA analyzed using Illumina Inf450K arrays. The data were downloaded from GEO (GSE34639).

Data set 52 (**CD4+T cells and CD14+monocytes**) consisted of sorted CD4+ T-cells and CD14+ monocytes from blood of an independent cohort of 25 healthy subjects [[25](#_ENREF_25)].

Data set 53 (**immortalized B cells)** and other cells from progeria and Werner syndrome patients and controls [[43](#_ENREF_43)]. The Hutchinson-Gilford Progeria Syndrome (HGP) and Werner Syndrome are two premature aging diseases showing features of common aging. Mutations in LMNA and WRN genes are associated to disease onset; however for a subset of patients the underlying causative mechanisms remains elusive. In this study, the authors aimed to evaluate the role of epigenetic alteration on premature aging diseases by performing genome-wide DNAm profiling of HGP and WS patients. The authors analyzed Epstein-Bar virus (EBV) immortalized B cells, naive B-cells, and peripheral blood mononuclear cells. The authors found aberrant DNAm profiles in the premature aging disorders Hutchinson-Gilford Progeria and Werner syndrome [[43](#_ENREF_43)]. In this relatively small data set, I found no evidence that these premature aging diseases accelerate DNAm age in immortalized B cells. Future studies could evaluate whether premature aging diseases are associated with accelerated DNAm age in other tissues or cell types. Interestingly, chronological age continued to be highly correlated with DNAm age in these immortalized B cells which suggests that immortalization via EBV does not have a major effect on DNAm age.

Data set 54 (**cerebellar** samples) and data set 55 (**occipital cortex samples**) from autism cases and controls [[44](#_ENREF_44)]. The authors collected idiopathic autistic and control cerebellar and BA19 (occipital) brain tissues. Here we ignored autism disease status. Incidentally, we could not detect an association between autism status and DNAm age.

Data set 56 (**breast**, normal adjacent tissue, Illumina 450K) consists of normal breast tissue samples from 90 female breast cancer cases (mean age 57, range 28-90) from TCGA, but unlike data set 57 these samples were assayed on the Illumina 450K platform.

Data set 57 (**breast,** normal adjacent tissue, Illumina 27K) consists of normal breast tissue samples from 27 female breast cancer cases (mean age 55, range 35-88) from the The Cancer Genome Atlas (TCGA) data base (http://tcga-data.nci.nih.gov/).

Data set 58 (**buccal cells**) from [[45](#_ENREF_45)]. The authors performed a longitudinal study of DNA methylation at birth and age 18 months in DNA from buccal swabs from 10 monozygotic (MZ) and 5 dizygotic (DZ) twin pairs from the Peri/postnatal Epigenetic Twins Study (PETS) cohort.

Data sets 59 (**colon**) normal adjacent tissue measured on the Illumina 450K array, downloaded from TCGA (COAD data).

Data set 60 (**adipose**) from monozygotic Twins Discordant for Type 2 Diabetes. [[46](#_ENREF_46)]. Monozygotic twins discordant for type 2 diabetes constitute an ideal model to study environmental contributions to type 2 diabetic traits. The authors aimed to examine whether global DNAm differences exist in major glucose metabolic tissues from twelve 53–80 year-old monozygotic discordant twin pairs. DNAm was measured by the Illumina HumanMethylation27 BeadChip in 22 (11 pairs) skeletal muscle and 10 (5 pairs) subcutaneous adipose tissue biopsies. Diabetes status was ignored in my analysis. I could find no significant evidence that disease status affects DNAm age in this small data set.

Data set 61 (**heart tissue**) consists of only 6 human male samples (mean age 61, range 55-71) [[47](#_ENREF_47)]. Clearly, larger sample sizes will be needed to evaluate this tissue.

Data set 62 (**kidney)** normal adjacent tissue from clear cell renal carcinoma consists of samples downloaded from the TCGA data base (KIRC) that were measured on the Illumina 27K platform.

Data set 63 (**liver normal adjacent tissues**) measured on the Illumina 450K platform from the TCGA data base (LIHC data).

Data sets 64 (**lung, normal adjacent tissue**) measured on the Illumina 450K arrays. The data consists of samples downloaded from TCGA data base (normal from LUAD).

Data set 65 (**muscle**) from monozygotic Twins Discordant for Type 2 Diabetes. [[46](#_ENREF_46)]. Monozygotic twins discordant for type 2 diabetes constitute an ideal model to study environmental contributions to type 2 diabetic traits. The authors aimed to examine whether global DNAm differences exist in major glucose metabolic tissues from twelve 53–80 year-old monozygotic discordant twin pairs. DNAm was measured by the Illumina HumanMethylation27 BeadChip in 22 (11 pairs) skeletal muscle and 10 (5 pairs) subcutaneous adipose tissue biopsies. Diabetes status was ignored in my analysis. I could find no significant evidence that disease status affects DNAm age in this small data set.

Data set 66 (**muscle**) tissue from healthy men who were 24 years old. These data came from an epigenetic analysis of healthy young men following a control and high-fat overfeeding diet [[48](#_ENREF_48)]. These data came from a randomized cross-over design, where all subjects received both treatments (control and high-fat overfeeding diet). Biopsies were obtained from 23 different individuals amounting to 22 samples following the control diet and 22 samples following the high-fat overfeeding diet (paired n=21). The resulting 44 samples were analyzed using the Illumina 27K platform. Diet status was ignored in my analysis. I could find no significant evidence that diet affects DNAm age in this relatively small data set.

Data set 67 (**placenta**) from [[49](#_ENREF_49)]. DNA from 20 third trimester early onset preeclampsia placentas and 20 gestational age matched controls.

Data sets 68 (**saliva)** from alcoholic females involved 52 samples (mean age 32, range 21-55) [[36](#_ENREF_36)].

Data set 69 (**uterine cervix**) involved cytologically normal cells from the uterine cervix of 152 women [[23](#_ENREF_23), [50](#_ENREF_50)].

Data set 70 (**uterine endometrium normal adjacent tissue**) measured on the Illumina 450K platform from the TCGA data base (UCEC data).

Data set 71 (**various human tissues**)from the ENCODE/HAIB Project. These Illumina 27K data were downloaded from GEO GSE40700.

Data set 72 (**chimpanzees and humans**) from [[47](#_ENREF_47)] The authors used the Illumina 27K array to compare DNAm profiles in the following human and chimpanzee tissue samples: 6 human livers, 6 human kidneys, 6 human heart, 6 chimpanzee livers, 6 chimpanzee kidneys, and 6 chimpanzee hearts.

Data set 73 (**ape blood**) from [[51](#_ENREF_51)]. The authors applied the Illumina 450K arrays to blood derived DNA from humans, chimpanzees, bonobos, gorillas and orangutans. Since ages were not available for humans and orangutans, I focused on chimpanzees, bonobos, gorillas for whom ages were available.

Data set 74 (**sperm**) from [[52](#_ENREF_52)]. The authors performed a genome-wide analysis of sperm DNA isolated from 21 men with a range of semen parameters presenting to a tertiary male reproductive health clinic. DNAm was measured with the Illumina Infinium array at 27,000 CpG loci.

Data set 75 (**sperm**) from [[53](#_ENREF_53)]. The authors applied the 450K platform to DNA derived from 26 normal sperm samples.

Data set 76 (**vascular endothelial cells from human umbilical cords**) from monozygotic and dizygotic twins [[34](#_ENREF_34)].

Data sets 77 and 78(**special cell types**) involved human embryonic stem cells, iPS cells, and somatic cell samples measured on the Illumina 27K array and Illumina 450K array, respectively [[54](#_ENREF_54)]. Although no specific age information was available, these two valuable data sets could be used a) to compare adult somatic tissues versus fetal somatic tissues (Additional File 8), b) to compare the DNAm ages of different tissues from the same individual (Figure 3), c) to assess the variance of methylation probes across adult somatic tissues and fetal somatic tissues (Additional File 8) d) to study how the DNAm age of iPS cells compares to that of somatic primary tissue and primary cell lines (Figure 6), e) to evaluate how cell passaging effects DNAm age (Figure 6). Data set 78 contained multiple tissue samples from two adults. For data set 78, the following tissues and sample sizes were available: Adipose (n=2 samples), Adrenal (n=4), Aorta (2), Bladder (2), Blood (2), Brain (3), Breast (1), Colon (1), Diaphragm (2), Duodenum (1), human embryonic stem (ES) cells (118), Gallbladder (1), Heart (2), iPS (46), Kidney (2), Liver (1), Lung (4), Lymph Node (2), Ovary (2), Pancreas (2), Prostate (1), Skeletal Muscle (2), Skin (1), Small Intestine (1), Somatic Primary Cell Line (49), Spleen (3), Stomach (4), Tongue (1) Ureter (2). For data set 52, the following sample sizes were available [[54](#_ENREF_54)] Adipose (2), Adrenal (5), Bladder (2), Blood (2), Brain (5), ES (19), Heart (5), iPSC (29), Kidney (5), Liver (4), Lung (7), Lymph Node (2), Pancreas (2), Skeletal Muscle (2), Somatic Primary Cell Line (22), Spleen (5), Stomach (6), Thymus (2), Tongue (2), Ureter (2).

Data set 79 (**reprogrammed mesenchymal stromal cells from human bone marrow (iP-MSC), initial MSC, and embryonic stem cells**) [[55](#_ENREF_55)]. The authors reprogrammed mesenchymal stromal cells from human bone marrow (iP-MSC) and compared their DNAm profiles with initial MSC and embryonic stem cells (ESCs) using the Illumina 450K array. The data were downloaded from GEO (GSE37066).

Data set 80 (**hESC and normal primary tissue**) from [[56](#_ENREF_56)]. The authors extracted DNA from the following well-characterized human embryonic stem cell (hESC)lines: SHEF-1, SHEF-4, SHEF-5, SHEF-7, H7, H14, H14S9, H7S14, HS181 and I3. The authors used DNA from human normal primary tissues provided by Biochain (Hayward, CA, USA).

Data set 81 (**hESC**) from [[57](#_ENREF_57)].DNA derived from H9, H13C, SHEF2 hESC cultured in two different media. The medium was not significantly related with DNAm age estimate.

Data set 82 (**blood cell type data**) [[58](#_ENREF_58)] Six healthy male blood donors, age 38 ± 13.6 years, were included in the study. From each individual, global DNAm levels were analyzed in whole blood, peripheral blood mononuclear cells (PBMC) and granulocytes as well as for seven isolated cell populations (CD4+ T cells, CD8+ T cells, CD56+ NK cells, CD19+ B cells, CD14+ monocytes, neutrophils, and eosinophils), n=60 samples analyzed in total. The data were downloaded from GEO (GSE35069).

# Criteria guiding the choice of the training sets

The choice of training data sets was guided by the following criteria: First, the training data should represent a wide spectrum of tissues and cell types. As can be seen from Additional file 1, the training data involve blood (whole blood, cord blood, PBMCs), brain (cerebellum, frontal cortex, pons, prefrontal cortex, temporal cortex, neurons and glial cells), breast, buccal epithelium, cartilage, colon, dermal fibroblasts, epidermis, gastric tissue, head/neck tissue, heart, kidney, liver, lung, mesenchymal stromal cells, prostate, saliva, stomach, thyroid, etc.

Second, the mean age in the training data (43 years, standard deviation= 25) should be comparable to that of the test data (42 years, SD=25). Third, the training data should contain a high proportion of samples (37%) measured on the Illumina 450K platform since many on-going studies use this recent Illumina platform. Incidentally, 34% of test set samples were measured on the 450K platform. Here I only studied 21369 probes measured with the Infinium type II assay which satisfied the following criteria: a) they were present on both Illumina platforms (Infinium 450K and 27K) and b) had fewer than 10 missing values.

# Description of the cancer data sets

An overview of the publicly available cancer DNAm data sets can be found in Additional file 1. The table reports the sample size, median age, age range, and tissue source, citation. All data are publicly available as can be seen from the column that reports GSE identifiers from the **Gene Expression Omnibus (GEO)** database and other online resources. More details on the individual data sets are given in the following.

Data set 3 (**glioblastoma multiforme**, GBM) measured on the Illumina 450K array from [[59](#_ENREF_59)] (GEO identifier GSE36278).

Data set 4 (**breast cancer**) measured on the Illumina 27K array from [[60](#_ENREF_60)] (GEO identifier GSE31979).

Data set 5 (**breast cancer**) measured on the Illumina 27K array from [[61](#_ENREF_61)](GEO identifier GSE20712).

Data set 6 (**breast cancer**) measured on the Illumina 27K array from [[23](#_ENREF_23)] (GEO identifier GSE33510).

Data set 10 (**colorectal cancer**) measured on the Illumina 27K array from [[62](#_ENREF_62)] (GEO identifier GSE25062).

Data set 23 (**prostate cancer**) measured on the Illumina 27K array from [[35](#_ENREF_35)] (GEO identifier GSE26126).

Data set 30 (**urothelial carcinoma**) measured on the Illumina 27L array from [[63](#_ENREF_63)].

All other cancer data sets came from the **TCGA** data base. In particular, acute myeloid leukemia (AML), bladder urothelial carcinoma (BLCA), cervical squamous cell carcinoma and endocervical adenocarcinoma (CESC), colon adenocarcinoma (COAD), head/neck squamous cell carcinoma (HNSC), liver hepatocellular carcinoma (LIHC), kidney renal clear cell carcinoma (KIRC), kidney renal papillary cell carcinoma (KIRP), liver ovarian serous cystadenocarcinoma (OVAR), prostate adenocarcinoma (PRAD), rectum adenocarcinoma (READ), sarcoma (SARC), thyroid carcinoma (THCA), skin cutaneous melanoma (SKCM), uterine corpus endometrioid carcinoma (UCEC).

# DNAm profiling and pre-processing steps

Full experimental methods and detailed descriptions of these public data sets can be found in the original references. The following briefly summarizes the main steps. Methylation analysis was performed either using the Illumina Infinium Human Methylation27 BeadChip [[64](#_ENREF_64)] or the Illumina Infinium HumanMethylation450 BeadChip. The Illumina HumanMethylation27 BeadChips measures bisulfite-conversion-based, single-CpG resolution DNAm levels at 27,578 different CpG sites within 5' promoter regions of 14,475 well-annotated genes in the human genome. Data from the two platforms were merged by focusing on the roughly 26k CpG sites that are present on both platforms. The HumanMethylation27 BeadChip mainly represents specific CpG that are located near gene promoter regions.

All of the public data were generated by following the standard protocol of Illumina methylation assays, which quantifies DNAm levels by the β value using the ratio of intensities between methylated (signal A) and un-methylated (signal B) alleles. Specifically, the β value was calculated from the intensity of the methylated (M corresponding to signal A) and un-methylated (U corresponding to signal B) alleles, as the ratio of fluorescent signals β= Max(M,0)/[Max(M,0)+Max(U,0)+100]. Thus, β values range from 0 (completely un-methylated) to 1 (completely methylated) [[65](#_ENREF_65)].

The mean inter-array correlation was used to measure how similar (correlated) a given sample is compared to the remaining samples of the data set. To ensure high quality data without technical artifacts, non-cancer samples were only used if their mean inter-array correlation was larger than 0.90 and if their maximum DNAm level (across all probes) was larger than 0.96. This filtering step was *not* applied to the cancer samples since it is well known that cancer greatly affects the DNAm levels. It is worth mentioning that my results would barely change if all samples had been used.

# Normalization methods for the DNA methylation data

I carried out several normalization steps to ensure that these data are comparable. While quantile normalization is often used in gene expression studies, it is less frequently used in DNAm studies. Before explaining my unbiased normalization strategy, I briefly provide some background. The Illumina 450K platforms uses two different chemical assays. The Infinium I and Infinium II assays for the assessment of the DNAm status of more than 480,000 cytosines distributed over the whole genome. The older Illumina 27K platform only uses the Infinium II assays. Several authors have noted that the data generated by the two chemical assays used by the 450K platform are not entirely compatible [[66](#_ENREF_66)]. Dedeurwaerder et al (2011) showed that their correction technique called 'peak-based correction', which rescales type II probes on the basis of type I probes greatly improved the signal in Illumina Inf450K data. Similarly, Maksimovic et al (2012) showed that their subset-quantile within array normalization (SWAN) substantially improves the results for the Illumina 450K platform [[67](#_ENREF_67)]. Unfortunately, I could not adopt the SWAN normalization here since it requires idat input files, which were not available for many of the data sets.

Teschendorff et al (2012) developed a model-based intra-array normalization strategy for the 450K platform, called BMIQ (Beta MIxture Quantile dilation), which adjusts beta-values of type II probes into a statistical distribution characteristic of type I probes[[68](#_ENREF_68)].

My own studies support the claim of these authors that normalizing type II probes so that they correspond to type I probes is a very useful pre-processing step for any study using the Illumina 450K platform. I could not adopt these techniques directly since my study only involves type II probes from the 27K platform. About 26000 CpGs from the 27K platform are also represented on the 450K platform and have the same probe identifier. Therefore, it is straightforward to merge data from the two platforms as long as one restricts attention to these overlapping probes. The age predictor was trained on the roughly 21368 type II probes that a) are shared between the Illumina 27K and the 450K platforms and b) had <= 10 missing values across the training data. However, I adopted the idea underlying these articles as follows. Instead of using type I probes as gold standard for rescaling type II probes, I created another gold standard by forming the mean DNAm value in the largest single study of this article (data set 1, i.e. whole blood samples from [[13](#_ENREF_13)]). Next, I adapted the BMIQ R function from Teschendorff et al (2013) [[68](#_ENREF_68)] so that it would rescale the overlapping 21k probes of each array so that their distribution matched that of the new gold standard. My empirical studies showed that this pre-processing step improved the accuracy of the resulting age predictor especially when it comes to the median error.

# Explicit details on the definition of DNAm age

Based on the training set data, I found that it is advantageous to transform age before carrying out a elastic net regression analysis. Toward this end, I used the following function F for transforming age:

F(age)=log(age+1)-log(adult.age+1) if age<= adult.age.

F(age)=(age-adult.age)/(adult.age+1) if age> adult.age.

The parameter adult.age was set to 20 for humans and 15 for chimpanzees. Note that F satisfies the following desirable properties: it

i) is a continuous, monotonically increasing function (which can be inverted),

ii) has a logarithmic dependence on age until adulthood (here set at 20 years),

iii) has a linear dependence on age after adulthood (here set to 20),

iv) is defined for negative ages (i.e. prenatal samples) by adding 1 (year) to age in the logarithm,

v) it has a continuous first derivative (slope function). In particular the slope at age=adult.age is given by 1/(adult.age+1).

The function F is visualized in Figure 6B,C by a red line. As expected, the red line passes through the weighted average of the CpGs (i.e. the linear part of the regression model). The inverse of the function F, denoted by inverse.F, is used to transform the linear part of the regression model into DNAm age.

An elastic net regression model (implemented in the glmnet R function) was used to regress a transformed version of age on the roughly 21k beta values in the training data. The elastic net regression results in a linear regression model whose coefficients b_0_, b_1_, ..., b_353_ relate to transformed age as follows

$F\left( chronological age \right)=b_{0}+b_{1}CpG_{1}+\ldots+b_{353}CpG_{353}$+error

The coefficient values can be found in Additional file 3. Based, on the coefficient values from the regression model, DNAmAge is estimated as follows

$${DNAmAge=inverse.F(b}_{0}+b_{1}CpG_{1}+\ldots+b_{353}CpG_{353})$$

Thus, the regression model can be used to predict the transformed age value by simply plugging the beta values of the selected CpGs into the formula. The linear part, (i.e. the weighted average of the selected CpGs) is visualized as red line in Figure 6B,C.

The glmnet function requires the user to specify two parameters (alpha and beta). Since I used an elastic net predictor, alpha was set to 0.5. But the lambda value of 0.02255706 was chosen by applying a 10 fold cross validation to the training data (via the R function cv.glmnet).

The following R code provides details on the analysis.

library(glmnet)

# use 10 fold cross validation to estimate the lambda parameter

# in the training data

glmnet.Training.CV = cv.glmnet(datMethTraining, F(Age), nfolds=10,alpha=alpha,family="gaussian")

# The definition of the lambda parameter:

lambda.glmnet.Training = glmnet.Training.CV$lambda.min

# Fit the elastic net predictor to the training data

glmnet.Training = glmnet(datMethTraining, F(Age), family="gaussian", alpha=0.5, nlambda=100)

# Arrive at an estimate of of DNAmAge

DNAmAgeBasedOnTraining=inverse.F(predict(glmnet.Training,datMeth,type="response",s=lambda.glmnet.Training))

# Chromatin state data used for Additional File 9

While specific histone modifications correlate with regulator binding, transcriptional initiation and elongation, enhancer activity and repression, combinations of chromatin modifications can provide even more precise insight into chromatin state [[69](#_ENREF_69)]. Here I used the chromatin state data from [[69](#_ENREF_69)]. The authors profiled nine human cell types, including common lines designated by the ENCODE consortium and primary cell types. These consisted of embryonic stem cells (H1 ES), erythrocytic leukemia cells (K562), B-lymphoblastoid cells (GM12878), hepatocellular carcinoma cells (HepG2), umbilical vein endothelial cells (HUVEC), skeletal muscle myoblasts (HSMM), normal lung fibroblasts (NHLF), normal epidermal keratinocytes (NHEK), and mammary epithelial cells (HMEC).

Ernst et al (2011) distinguish six broad classes of chromatin states, referred to as promoter, enhancer, insulator, transcribed, repressed, and inactive states. Within them, active, weak and poised promoters (states 1-3) differ in expression levels, strong and weak candidate enhancers (states 4-7) differ in expression of proximal genes, and strongly and weakly transcribed regions (states 9-11) also differ in their positional enrichments along transcripts. Similarly, Polycomb-repressed regions (state 12) differ from heterochromatic and repetitive states (states 13-15), which are also enriched for H3K9me3. It will be interesting to map the 353 clock CpGs to the states of individual cell lines. Since the number of profiled cell lines keeps expanding and warrants a comprehensive analysis, reporting results for individual cell lines is beyond the scope of this article. Instead, I provide a broad overview by averaging the results across the 9 cell lines mentioned by Ernst 2011. Specifically, the y-axis of Additional file 9 reports the mean number of cell lines (out of 9 cell lines) for which the CpGs were in the chromatin state mentioned in the title.

# Comparing the multi-tissue predictor with other age predictors

Several recent publications describe age predictors based on DNA methylation levels [[2](#_ENREF_2), [3](#_ENREF_3), [16](#_ENREF_16)]. Hannum et al (2012) found that computing a DNAm based age predictor for different tissues gave basically no overlap, e.g. blood-derived predictive CpGs were different from those from other tissues [[16](#_ENREF_16)]. This suggests that an optimal age predictor for one tissue may be sub-optimal for another. I don't disagree with these results. Instead, I show that one can build a multi-tissue age predictor which can be used for addressing a wide range of questions arising in aging research. While slight gains in accuracy can probably be achieved by focusing on a single tissue and considering more CpGs, the major strength of the proposed multi-tissue age predictor lies in its wide applicability: for most tissues it will not require any adjustments or offsets. The proposed multi-tissue age predictor greatly outperforms the predictors by [[2](#_ENREF_2), [3](#_ENREF_3)] as detailed below. I could not directly evaluate the predictor by [[16](#_ENREF_16)] since a) only seven out of its 71 CpGs are represented on the Illumina 27K platform, b) it included gender and body mass index as covariates. However, I was able to evaluate the performance of a sparse version of the published predictor by using the seven overlapping CpGs that could be found on both Illumina platforms. In the following, I provide more details. To provide an unbiased comparison, I constructed each predictor in an analogous fashion in the training data, i.e. its coefficient values were estimated using the same penalized regression approach. Thus, the predictors only differed with respect to the sets of CpGs that were considered in the penalized regression model. While this does not allow me to assess the performance of the published predictors directly, it provides a completely unbiased comparison of the age predictors. Using the coefficient values from the respective publications would have biased the comparison against them since most were constructed on significantly smaller training data sets (often involving a single tissue) or using a single Illumina platform.

I evaluated the performance of each age predictor a) across the training data sets and b) across the test data sets. Since I constructed each predictor using the training data sets, the estimated accuracy in the training set is overly optimistic. I also defined a "shrunken" version of my multi-tissue age predictor, which only involves a subset of 110 CpGs from the 353 CpGs. As indicated by its name, the shrunken predictor is defined by using a more stringent shrinkage parameter (50 times that of the original model) in the penalized regression model. The shrunken predictor is highly accurate in the training data (cor=0.95, error=4 years) and test data (cor=0.95, error=4.2 years). Coefficient values of the multi-tissue predictor and its shrunken version can be found in Additional File 3. I find that my multi-tissue age predictor greatly outperforms the predictors by [[2](#_ENREF_2), [3](#_ENREF_3)]. Even when I use the same penalized regression approach for re-training their CpGs, both predictors lead to high errors in training and test data (>14 years) and much lower age correlations (<=0.56). Hannum et al (2012) proposed an age predictor based on 71 CpGs [[16](#_ENREF_16)]. The authors built a predictive model of aging using a penalized regression method (elastic net) but it differs from the current analysis in the following aspects. First, the aging model from [[16](#_ENREF_16)] was trained on whole blood, which is a noteworthy advantage when it comes to the design of practical diagnostics and for testing blood samples collected from other studies. Second, it also included clinical parameters such as gender and body mass index as covariates. Third, it is based on CpGs from the Illumina 450K arrays while my predictor only involves CpGs from the Illumina 27K array. Since only seven of the 71 CpG markers from [[16](#_ENREF_16)] can be found on the Illumina 27K array, I could not carry out a direct comparison across the many tissues considered here. Instead, I was only able to evaluate the performance of a very sparse version of the published predictor by using the seven overlapping CpGs (cg04474832, cg05442902, cg06493994, cg09809672, cg19722847, cg21296230, cg22736354) that could be found on both Illumina platforms. The resulting sparse version performs well in the training data (age cor=0.82, error=8.0 years) and in the test data (cor=0.86, error=8.0 years).

In conclusion, a sparse version of the predictor from [[16](#_ENREF_16)](based on 7 CpGs) works best among predictors with fewer than 10 CpGs. The proposed multi-tissue predictor suggests that a couple of hundred CpGs will be needed to accurately predicted age across multiple tissue types and the two Illumina platforms.

# Meta analysis for finding age-related CpGs

To measure pure age effects in the marginal analysis (Additional file 9), I used the *metaAnalysis* R function in the WGCNA R package [[70](#_ENREF_70)]. This function allowed to to calculate two p-values: *pValueHighScale* and *pValueLowScale* for finding consistently positively and negatively age related CpGs, respectively. Thus, CpGs with a low *pValueHighScale* have a consistently high age correlation in the individual data sets. Since this meta analysis method conditions on the data sets, the p-values are not confounded by data set or tissue. I used the signed logarithm (base 10) of the meta analysis p-value in scatter plots (Additional file 8E,F). The sign was chosen so that CpGs with positive (negative) age correlations lead to positive (negative) log p-values. Additional file 8E shows that the meta analysis p-value based on the training data sets is highly correlated with a corresponding meta analysis p-value calculated using all training and test sets. The high correlation shows that little information is lost by focusing on the training data. The most significant age-related CpGs found in all data can already be found using the training data alone.

# Variation of age related CpGs across somatic tissues

Since the age predictor performs well across a wide spectrum of tissues, I hypothesized that many of the 353 CpGs used for estimating DNAm age vary little across tissues and that many of them correlate highly with age.

To test this hypothesis, I first defined three different measures of tissue variance. The first measure of tissue variance used analysis of variance (ANOVA) across the training data sets. Toward this end, I used a multivariate regression model to regress each CpG (dependent variable) on age and tissue type. The regression model included age as covariate since the analysis needed to adjust for the fact that different data sets had different age distributions (Additional File 1). ANOVA allowed me to calculate an F statistic for tissue effect which takes on a large value for CpGs that vary greatly across the different training set tissues. The second and third measure of tissue variance were defined using the adult somatic tissues and the fetal somatic tissues, respectively, from [[54](#_ENREF_54)] (Additional File 1, data set 77). As an aside, I mention that the mean DNAm age (predicted age) of fetal somatic tissues is close to zero, i.e. it is much lower than that of adult somatic tissues in this data set, which again validates the age predictor. The adult- and the fetal measure of tissue variance of each CpGs is defined by its variance across the adult and somatic tissue samples from [[54](#_ENREF_54)], respectively. I find that the adult and the fetal tissue variance measures are highly correlated (cor=0.8, Additional File 8A) which indicates that these measures are robustly defined and change little with age. Since the data from Nazor et al (data set 77 in Additional File 1) were not part of the training data, these measures could be used to validate the F-statistic measure of tissue variance. I find a high correlation between the adult measure of tissue variance and the F statistic (cor=0.73, Additional File 8D) which shows that these measures of tissue variance are highly reproducible. I also defined a stringent measure of age variation for each CpG using a meta analysis approach. The meta analysis calculated age correlations in each training data set separately and next aggregated the correlation test p-values resulting in a meta analysis p-value. Different from the construction of the age predictor, the meta analysis approach explicitly conditioned on each data set. Thus, a CpG has a significant meta analysis p-value if it consistently correlates with age irrespective of tissue type, data set effect, or Illumina platform version. It did not really matter that I calculated the meta analysis p-value using the training data alone since the resulting p-value is highly correlated (cor=0.97, Additional File 8E) with the analogous p-value that results from using all data sets.

To address the question how the tissue variation of a CpG relates to its age variation, I plotted tissue variance versus age variance. Using the ANOVA F statistic for tissue effect, I find the that CpGs with high positive or negative age correlations do not vary much across the somatic adult tissues (Additional File 8F). A completely analogous result can be observed when using the somatic variance measures involving the adult and fetal tissues from Nazor et al (data 77). CpGs that vary little across tissues appear to be more susceptible to aging effects. Conversely, CpGs that vary greatly across tissues are less affected by aging effects which might reflect that they are actively protected against aging effects. The red and blue dots in Additional File 8 correspond to the 353 CpGs used for estimating DNAm age. Additional File 8 also shows that the most significant CpGs were selected by the age predictor.

# Studying age effects using gene expression data

The publicly available microarray data sets involved mainly healthy individuals (in particular no cancer samples were considered).

To estimate the age effect on gene expression levels, I analyzed multiple independent publicly available microarray data sets. Blood microarray data sets involving mainly healthy control individuals (referred to as SAFHS [[71](#_ENREF_71)], Chaussabel [[72](#_ENREF_72)] and NOWAC [[73](#_ENREF_73)] data) and the CD8 T cell microarray data Cao [[74](#_ENREF_74)]. To assess whether a gene was differentially expressed between naive CD8+ T cells and antigen exposed CD8+ T cells, I used the data from Willinger et al [[75](#_ENREF_75), [76](#_ENREF_76)]). In the following I provide more details.

The data from a study of post-menopausal women (the NOWAC data). In my largest expression data set, the San Antonio Family Heart Study (SAFHS) data set, individuals were ascertained from probands meeting two criteria: 1) having a living spouse and 2) having six first-degree relatives 16 years or older in the San Antonio area – excluding parents. While this data set was used to study cardiovascular phenotypes, the data was obtained without selection bias towards these traits, and therefore can be considered a random sampling.

I obtained the San Antonio Family Heart Study (SAFHS) blood data set, which was previously analyzed by Goring, et al [[71](#_ENREF_71)].This data set was derived from lymphocytes; RNA was hybridized to Illumina Sentrix Human Whole Genome (WG-6) Series I BeadChips with probe sets corresponding to 18,544 genes. Quantile normalization was applied to the raw data. This data set consisted of 1,084 samples: 452 males and 632 females between ages 15 and 94 after outlier removal. Specifically, outlier detection and removal was performed using an iterative process of removing outliers with average interarray correlation (IAC) ≤ 2 SD below the mean until visual inspection of the cluster dendrogram and plot of the mean IAC revealed no further outliers. This analysis was completely unbiased and agnostic to chronological age. Toward this end, I used our recently developed sampleNetwork R function described in [[77](#_ENREF_77)]

The Chaussabel data set was originally published by Pankla, et al.[[72](#_ENREF_72)] and was used to study melioidosis. 67 whole blood samples were hybridized to Illumina Sentrix Human-6 V2 BeadChip arrays with 12,483 genes. Background subtraction and average normalization was performed using Illumina BeadStudio version 2 software, and standard normalization for one-color array data was performed using Gene-Spring GX7.3 software (Agilent Technologies) by the original authors. This data set consisted of 35 men and 32 women between the ages of 18 and 74. I also used healthy postmenopausal women from the Norwegian Women and Cancer (NOWAC)study [[73](#_ENREF_73)]. The whole blood data were measured using AB Human Genome Survey Microarray V2.0 with 16,753 genes. For sets of technical replicates, arrays with the least number of probes with a S/N > 3 were excluded. Arrays with less than 40% of probes with a S/N ≥ 3 were removed. Probes with an S/N > 3 in less than 50% of samples were excluded. Log (base 2) transformation, quantile normalization and imputation was performed. I furthermore excluded samples using an iterative process of removing samples with average interarray correlation <2 SD ultimately resulting in 245 samples. Age ranges of [48,53), [53,58) and [58,63] were given, and I used for the analysis corresponding ages of 50, 55 and 60.

In the CD8+ T cell data set from Cao, et al.[[74](#_ENREF_74)] Affymetrix HG-U133A_2 Gene Arrays were used to explore the expression profiles of three male and six female donors whose ages ranged from 23 to 81. Microarray Suite Version 5.0 (MAS 5.0; Affymetrix) was used to quantify the expression levels of 12,483 genes. In the CD8+ T cell data set from Willinger et al [[75](#_ENREF_75), [76](#_ENREF_76)], Affymetrix HG-U133 plus 2.0 arrays (log transformed MAS5 data) were used to explore the expression profiles of human CD8+ naive T cells (TN), central memory (TCM), effector memory (TEM), and effector memory RA (TEMRA) CD8+ T cells. TN can be regarded as peripheral stem cells, while TEM and TEMRA are differentiated cells with effector function. For each T cell type, the original data set contained 4 replicates (i.e. there were 16 arrays). Since one of the central memory samples had very low interarray correlation with the other samples, I removed this potential outlier from the analysis. A Student t-test of differential expression was used to compare expression levels in naive CD8+ cells versus the memory T cells.

The first brain data set was previously analyzed by Lu, et al. [[78](#_ENREF_78)]. 30 frontal lobe samples were hybridized to Affymetrix HG-U95Av2 oligonucleotide arrays with 8,760 genes. Arrays were normalized by Lu, et al. using dChip V1.3 software, and after using the aforementioned iterative process of removing samples with average interarray correlation <2 SD below the mean I obtained 25 samples. This data set consisted of 16 men and 9 women between ages 26 and 91.

The second cortical brain data set was previously analyzed by Myers, et al. [[79](#_ENREF_79)]. The Illumina HumanRef-8 Expression BeadChip was utilized, and expression profiles were rank-invariant normalized using Illumina BeadStudio software. I utilized an iterative normalization process and removed 25 samples for a total of 168 samples and 19,880 genes. This data set consisted of 92 men and 76 women between ages 65 and 100. The third cortical brain data set was previously analyzed by Oldham, et al. [[80](#_ENREF_80)].Affymetrix HG-U95Av2 microarrays were used. Quantile normalization was utilized. Ultimately I identified 7763 genes in 67 individuals. This data set consisted of 48 men and 19 women between ages 22 and 81. The kidney data sets were previously analyzed by Rodwell, et al. [[81](#_ENREF_81)]. I utilized data from HG-U133A high-density oligonucleotide arrays; Rodwell, et al. normalized data using the dChip program according to the stable invariant set, and I further processed using the normalization and iterative outlier removal process. These normalization and outlier detection procedures resulted in 63 kidney cortex samples and 52 kidney medulla samples. There were 12,606 genes in both data sets. The kidney cortex data set consisted of 35 men and 26 women between ages 27 and 87, and the kidney medulla data set consisted of 29 men and 23 women between ages 29 and 92.

The muscle data set was previously analyzed by Zahn, et al. [[82](#_ENREF_82)]. 81 samples were hybridized to Affymetrix HG-U133 2.0 Plus high-density oligonucleotide arrays. The authors used the DChip program to normalize the data. I omitted 10 samples using the iterative normalization and outlier removal process, resulting in 71 samples and 19,621 genes. This data set consisted of 39 men and 32 women between ages 16 and 89.

# Meta analysis applied to gene expression data

In the following, I describe how I obtained the Pearson correlation coefficient, the corresponding t-test statistic Z in each data set, the metaZ statistics summarizing correlation test statistics across multiple data, a corresponding empirical p-value (pMetaZ). I denote by r_s_ the Pearson correlation coefficient (e.g. between age and the gene expression profile) in the s-th data set. The Student t-test statistic for testing whether the correlation is different from zero is given by $Z_{s}=\frac{\sqrt{m_{s}-2}\cdot r_{s}}{\sqrt{1-r_{s}^{2}}}$where m_s_ denotes the number of observations (i.e. microarrays, individuals) in the s-th data set. This Z statistic is equivalent to the Wald test statistic resulting from a univariate regression model where age is regressed on the gene expression profile. To combine multiple correlation test statistics across the data sets, I used the metaZ statistic $metaZ=\frac{\sum_{s=1}^{no.dataSets} w_{s}Z_{s}}{\sqrt{\sum_{s=1}^{no.dataSets} \left(w_{s}\right)^{2}}}$ where w_s_ denotes a weight associated with the s-th data set. All data sets received a weight of w_s_=1 but the weight had a negligible effect. Under the null hypothesis of zero correlation, metaZ follows an approximate normal distribution under weak assumptions, which will be outlined in the following. First, metaZ follows approximately a standard normal distribution if each individual Z_s_ follows approximately a standard normal distribution since the data sets are independent. Second, even if individual Z statistics do not follow a normal distribution, one can invoke the central limit theorem if many independent data sets are being considered.

# Names of the genes whose mutations are associated with age acceleration

Mutations in the following genes either increase or decrease DNAm age.

AKAP9–A kinase (PRKA) anchor protein (yotiao) 9

CHD7–chromodomain helicase DNA binding protein 7 [Homo sapiens]

CTNND2–catenin (cadherin-associated protein), delta 2

DMBT1–deleted in malignant brain tumors 1

DSG3–desmoglein 3

FAM123C–family with sequence similarity 123C

FAT4–FAT atypical cadherin 4

GATA3–GATA binding protein 3

KCNB1–potassium voltage-gated channel, Shab-related subfamily, member 1

LEPR–leptin receptor

MACF1–microtubule-actin crosslinking factor 1

MB21D1–Mab-21 domain containing 1

MGAM–maltase-glucoamylase (alpha-glucosidase)

MUC17–mucin 17, cell surface associated

MYH7–myosin, heavy chain 7, cardiac muscle, beta

RELN–reelin

THOC2–THO complex 2

TMEM132D–transmembrane protein 132D

TTN–titin

TP53–tumor protein p53

U2AF1–U2 small nuclear RNA auxiliary factor 1

# Is DNAm age a biomarker of aging?

The American Federation for Aging Research proposed the following criteria for a biomarker of aging (reviewed in [[83-85](#_ENREF_83)]):

1. It must predict the rate of aging.

2. It must monitor a basic process that underlies the aging process, not the effects of disease.

3. It must be able to be tested repeatedly without harming the person.

4. It must be something that works in humans and in laboratory animals.

I will address these criteria in reverse order. DNAm age probably meets criterion 4 if chimpanzees are acceptable as lab animals (given my results in Figure 4). There is a good chance that it meets criterion 3 (given my results in blood, saliva, buccal cells, skin) and criterion 2 (see my EMS model of DNAm age and the vast literature on aging effects on DNA methylation levels). Large cohort studies will be very valuable for addressing criterion 1. These studies need to test whether a measure of DNAm based age acceleration will, in the absence of disease, better predict functional capability than chronological age [[86](#_ENREF_86)].

# Online DNAm age calculator

I plan to develop a user-friendly web implementation of this software in the near future; please see my website http://labs.genetics.ucla.edu/horvath/htdocs/dnamage/ for updates.

# References

1. Koch CM, Suschek CV, Lin Q, Bork S, Goergens M, Joussen S, Pallua N, Ho AD, Zenke M, Wagner W: **Specific Age-Associated DNA Methylation Changes in Human Dermal Fibroblasts.** *PLoS ONE* 2011, **6:**e16679.

2. Koch C, Wagner W: **Epigenetic-aging-signature to determine age in different tissues.** *Aging* 2011, **3:**1018–1027.

3. Bocklandt S, Lin W, Sehl ME, Sanchez FJ, Sinsheimer JS, Horvath S, Vilain E: **Epigenetic predictor of age.** *PLoS ONE* 2011, **6:**e14821.

4. Esteller M: **Epigenetic lesions causing genetic lesions in human cancer: promoter hypermethylation of DNA repair genes.** *European Journal of Cancer* 2000, **36:**2294-2300.

5. Ushijima T: **Detection and interpretation of altered methylation patterns in cancer cells.** *Nat Rev Cancer* 2005, **5:**223-231.

6. So K, Tamura G, Honda T, Homma N, Waki T, Togawa N, Nishizuka S, Motoyama T: **Multiple tumor suppressor genes are increasingly methylated with age in non-neoplastic gastric epithelia.** *Cancer Science* 2006, **97:**1155-1158.

7. Fraga MF, Esteller M: **Epigenetics and aging: the targets and the marks.** *Trends in Genetics* 2007, **23:**413-418.

8. Fraga MF, Agrelo R, Esteller M: **Cross-Talk between Aging and Cancer.** *Annals of the New York Academy of Sciences* 2007, **1100:**60-74.

9. Bjornsson HT, Sigurdsson MI, Fallin MD, Irizarry RA, Aspelund T, Cui H, Yu W, Rongione MA, Ekström TJ, Harris TB, et al: **Intra-individual Change Over Time in DNA Methylation With Familial Clustering.** *JAMA: The Journal of the American Medical Association* 2008, **299:**2877-2883.

10. Christensen B, Houseman E, Marsit C, Zheng S, Wrensch M, Wiemels J, Nelson H, Karagas M, Padbury J, Bueno R, et al: **Aging and Environmental Exposures Alter Tissue-Specific DNA Methylation Dependent upon CpG Island Context.** *PLoS Genet* 2009, **5:**e1000602.

11. Rodríguez-Rodero S, Fernández-Morera J, Fernandez A, Menéndez-Torre E, Fraga M: **Epigenetic regulation of aging.** *Discov Med* 2010, **10:**225-233.

12. Teschendorff AE, Menon U, Gentry-Maharaj A, Ramus SJ, Weisenberger DJ, Shen H, Campan M, Noushmehr H, Bell CG, Maxwell AP, et al: **Age-dependent DNA methylation of genes that are suppressed in stem cells is a hallmark of cancer.** *Genome Res* 2010, **20:**440-446.

13. Horvath S, Zhang Y, Langfelder P, Kahn R, Boks M, van Eijk K, van den Berg L, Ophoff RA: **Aging effects on DNA methylation modules in human brain and blood tissue.** *Genome Biology* 2012, **13**.

14. Issa J-PJ, Ottaviano YL, Celano P, Hamilton SR, Davidson NE, Baylin SB: **Methylation of the oestrogen receptor CpG island links ageing and neoplasia in human colon.** *Nat Genet* 1994, **7:**536-540.

15. Maegawa S, Hinkal G, Kim HS, Shen L, Zhang L, Zhang J, Zhang N, Liang S, Donehower LA, Issa J-PJ: **Widespread and tissue specific age-related DNA methylation changes in mice.** *Genome Res* 2010, **20:**332-340.

16. Hannum G, Guinney J, Zhao L, Zhang L, Hughes G, Sadda S, Klotzle B, Bibikova M, Fan J-B, Gao Y, et al: **Genome-wide Methylation Profiles Reveal Quantitative Views of Human Aging Rates.** *Molecular cell* 2012.

17. Alisch RS, Barwick BG, Chopra P, Myrick LK, Satten GA, Conneely KN, Warren ST: **Age-associated DNA methylation in pediatric populations.** *Genome Res* 2012, **22:**623-632.

18. Harris R, Nagy-Szakal D, Pedersen N, Opekun A, Bronsky J, Munkholm P, Jespersgaard C, Andersen P, Melegh B, Ferry G, et al: **Genome-wide peripheral blood leukocyte DNA methylation microarrays identified a single association with inflammatory bowel diseases** *Inflamm Bowel Dis* 2012, **18:**2334-2341.

19. Adkins RM, Krushkal J, Tylavsky FA, Thomas F: **Racial differences in gene-specific DNA methylation levels are present at birth.** *Birth Defects Research Part A: Clinical and Molecular Teratology* 2011, **91:**728-736.

20. Gibbs JR, van der Brug MP, Hernandez DG, Traynor BJ, Nalls MA, Lai S-L, Arepalli S, Dillman A, Rafferty IP, Troncoso J, et al: **Abundant Quantitative Trait Loci Exist for DNA Methylation and Gene Expression in Human Brain.** *PLoS Genet* 2010, **6:**e1000952.

21. Numata S, Ye T, Hyde Thomas M, Guitart-Navarro X, Tao R, Wininger M, Colantuoni C, Weinberger Daniel R, Kleinman Joel E, Lipska Barbara K: **DNA Methylation Signatures in Development and Aging of the Human Prefrontal Cortex.** *The American Journal of Human Genetics* 2012, **90:**260-272.

22. Guintivano J, Aryee MJ, Kaminsky ZA: **A cell epigenotype specific model for the correction of brain cellular heterogeneity bias and its application to age, brain region and major depression.** *Epigenetics* 2013, **8:**290-302.

23. Zhuang J, Jones A, Lee S-H, Ng E, Fiegl H, Zikan M, Cibula D, Sargent A, Salvesen HB, Jacobs IJ, et al: **The Dynamics and Prognostic Potential of DNA Methylation Changes at Stem Cell Gene Loci in Women's Cancer.** *PLoS Genet* 2012, **8:**e1002517.

24. Essex MJ, Thomas Boyce W, Hertzman C, Lam LL, Armstrong JM, Neumann SMA, Kobor MS: **Epigenetic Vestiges of Early Developmental Adversity: Childhood Stress Exposure and DNA Methylation in Adolescence.** *Child Development* 2011, **84:**58-75.

25. Rakyan VK, Down TA, Maslau S, Andrew T, Yang TP, Beyan H, Whittaker P, McCann OT, Finer S, Valdes AM, et al: **Human aging-associated DNA hypermethylation occurs preferentially at bivalent chromatin domains.** *Genome Res* 2010, **20:**434-439.

26. Martino DJ, Tulic MK, Gordon L, Hodder M, Richman T, Metcalfe J, Prescott SL, Saffery R: **Evidence for age-related and individual-specific changes in DNA methylation profile of mononuclear cells during early immune development in humans.** *Epigenetics: official journal of the DNA Methylation Society* 2011, **6**.

27. Fernández-Tajes J, Soto-Hermida A, Vázquez-Mosquera ME, Cortés-Pereira E, Mosquera A, Fernández-Moreno M, Oreiro N, Fernández-López C, Fernández JL, Rego-Pérez I, Blanco FJ: **Genome-wide DNA methylation analysis of articular chondrocytes reveals a cluster of osteoarthritic patients.** *Annals of the Rheumatic Diseases* 2013**:**PMID: 23505229.

28. Harris RA, Nagy-Szakal D, Kellermayer R: **Human metastable epiallele candidates link to common disorders.** *Epigenetics* 2013, **8:**157-163.

29. Grönniger E, Weber B, Heil O, Peters N, Stäb F, Wenck H, Korn B, Winnefeld M, Lyko F: **Aging and Chronic Sun Exposure Cause Distinct Epigenetic Changes in Human Skin.** *PLoS Genet* 2010, **6:**e1000971.

30. Zouridis H, Deng N, Ivanova T, Zhu Y, Wong B, Huang D, Wu YH, Wu Y, Tan IB, Liem N, et al: **Methylation Subtypes and Large-Scale Epigenetic Alterations in Gastric Cancer.** *Science Translational Medicine* 2012, **4:**156ra140.

31. Haas J, Frese KS, Park YJ, Keller A, Vogel B, Lindroth AM, Weichenhan D, Franke J, Fischer S, Bauer A, et al: **Alterations in cardiac DNA methylation in human dilated cardiomyopathy.** *EMBO Molecular Medicine* 2013, **5:**413-429.

32. Shen J, Wang S, Zhang Y-J, Kappil M, Wu H-C, Kibriya MG, Wang Q, Jasmine F, Ahsan H, Lee P-H, et al: **Genome-wide DNA methylation profiles in hepatocellular carcinoma.** *Hepatology* 2012, **55:**1799-1808.

33. Bork S, Pfister S, Witt H, Horn P, Korn, B, Ho A, Wagner W: **DNA methylation pattern changes upon long-term culture and aging of human mesenchymal stromal cells.** *Aging Cell* 2010, **9:**54-63.

34. Gordon L, Joo JE, Powell JE, Ollikainen M, Novakovic B, Li X, Andronikos R, Cruickshank MN, Conneely KN, Smith AK, et al: **Neonatal DNA methylation profile in human twins is specified by a complex interplay between intrauterine environmental and genetic factors, subject to tissue-specific influence.** *Genome Res* 2012, **22:**1395-1406.

35. Kobayashi Y, Absher DM, Gulzar ZG, Young SR, McKenney JK, Peehl DM, Brooks JD, Myers RM, Sherlock G: **DNA methylation profiling reveals novel biomarkers and important roles for DNA methyltransferases in prostate cancer.** *Genome Res* 2011, **21:**1017-1027.

36. Liu J, Morgan M, Hutchison K, Calhoun VD: **A Study of the Influence of Sex on Genome Wide Methylation.** *PLoS ONE* 2010, **5:**e10028.

37. Song H, Ramus SJ, Tyrer J, Bolton KL, Gentry-Maharaj A, Wozniak E, Anton-Culver H, Chang-Claude J, Cramer DW, DiCioccio R, et al: **A genome-wide association study identifies a new ovarian cancer susceptibility locus on 9p22.2.** *Nat Genet* 2009, **41:**996-1000.

38. Liu Y, Aryee MJ, Padyukov L, Fallin MD, Hesselberg E, Runarsson A, Reinius L, Acevedo N, Taub M, Ronninger M, et al: **Epigenome-wide association data implicate DNA methylation as an intermediary of genetic risk in rheumatoid arthritis.** *Nat Biotech* 2013, **31:**142-147.

39. Heyn H, Li N, Ferreira HJ, Moran S, Pisano DG, Gomez A, Diez J, Sanchez-Mut JV, Setien F, Carmona FJ, et al: **Distinct DNA methylomes of newborns and centenarians.** *Proceedings of the National Academy of Sciences* 2012, **109:**10522-10527.

40. Lam LL, Emberly E, Fraser HB, Neumann SM, Chen E, Miller GE, Kobor MS: **Factors underlying variable DNA methylation in a human community cohort.** *Proceedings of the National Academy of Sciences* 2012, **109:**17253-17260.

41. Khulan B, Cooper WN, Skinner BM, Bauer J, Owens S, Prentice AM, Belteki G, Constancia M, Dunger D, Affara NA: **Periconceptional maternal micronutrient supplementation is associated with widespread gender related changes in the epigenome: a study of a unique resource in the Gambia.** *Human Molecular Genetics* 2012, **21:**2086-2101.

42. Martino D, Maksimovic J, Joo JH, Prescott SL, Saffery R: **Genome-scale profiling reveals a subset of genes regulated by DNA methylation that program somatic T-cell phenotypes in humans.** *Genes Immun* 2012, **13:**388-398.

43. Heyn H, Moran S, Esteller M: **Aberrant DNA methylation profiles in the premature aging disorders Hutchinson-Gilford Progeria and Werner syndrome.** *Epigenetics* 2013, **8:**28-33.

44. Ginsberg MR, Rubin RA, Falcone T, Ting AH, Natowicz MR: **Brain Transcriptional and Epigenetic Associations with Autism.** *PLoS ONE* 2012, **7:**e44736.

45. Martino D, Loke Y, Gordon L, Ollikainen M, Cruickshank M, Saffery R, Craig J: **Longitudinal, genome-scale analysis of DNA methylation in twins from birth to 18 months of age reveals rapid epigenetic change in early life and pair-specific effects of discordance.** *Genome Biology* 2013, **14:**R42.

46. Ribel-Madsen R, Fraga MF, Jacobsen S, Bork-Jensen J, Lara E, Calvanese V, Fernandez AF, Friedrichsen M, Vind BF, Højlund K, et al: **Genome-Wide Analysis of DNA Methylation Differences in Muscle and Fat from Monozygotic Twins Discordant for Type 2 Diabetes.** *PLoS ONE* 2012, **7:**e51302.

47. Pai AA, Bell JT, Marioni JC, Pritchard JK, Gilad Y: **A Genome-Wide Study of DNA Methylation Patterns and Gene Expression Levels in Multiple Human and Chimpanzee Tissues.** *PLoS Genet* 2011, **7:**e1001316.

48. Jacobsen SC, Brøns C, Bork-Jensen J, Ribel-Madsen R, Yang B, Lara E, Hall E, Calvanese V, Nilsson E, Jørgensen SW, et al: **Effects of short-term high-fat overfeeding on genome-wide DNA methylation in the skeletal muscle of healthy young men.** *Diabetologia* 2012, **55:**3341-3349.

49. Blair JD, Yuen RKC, Lim BK, McFadden DE, von Dadelszen P, Robinson WP: **Widespread DNA hypomethylation at gene enhancer regions in placentas associated with early-onset pre-eclampsia.** *Molecular Human Reproduction* 2013.

50. Teschendorff A, Jones A, Fiegl H, Sargent A, Zhuang J, Kitchener H, Widschwendter M: **Epigenetic variability in cells of normal cytology is associated with the risk of future morphological transformation.** *Genome Medicine* 2012, **4:**24.

51. Hernando-Herraez I, Prado-Martinez J, Garg P, Fernandez-Callejo M, Heyn H, Hvilsom C, Navarro A, Esteller M, Sharp A, Marques-Bonet T: **Dynamics of DNA Methylation in Recent Human and Great Apes Evolution.** *PLoS Genet* 2013, **In Press**.

52. Pacheco SE, Houseman EA, Christensen BC, Marsit CJ, Kelsey KT, Sigman M, Boekelheide K: **Integrative DNA Methylation and Gene Expression Analyses Identify DNA Packaging and Epigenetic Regulatory Genes Associated with Low Motility Sperm.** *PLoS ONE* 2011, **6:**e20280.

53. Krausz C, Sandoval J, Sayols S, Chianese C, Giachini C, Heyn H, Esteller M: **Novel Insights into DNA Methylation Features in Spermatozoa: Stability and Peculiarities.** *PLoS ONE* 2012, **7:**e44479.

54. Nazor Kristopher L, Altun G, Lynch C, Tran H, Harness Julie V, Slavin I, Garitaonandia I, Müller F-J, Wang Y-C, Boscolo Francesca S, et al: **Recurrent Variations in DNA Methylation in Human Pluripotent Stem Cells and Their Differentiated Derivatives.** *Cell stem cell* 2012, **10:**620-634.

55. Shao K, Koch C, Gupta MK, Lin Q, Lenz M, Laufs S, Denecke B, Schmidt M, Linke M, Hennies HC, et al: **Induced Pluripotent Mesenchymal Stromal Cell Clones Retain Donor-derived Differences in DNA Methylation Profiles.** *Mol Ther* 2012.

56. Calvanese V, Fernández AF, Urdinguio RG, Suárez-Alvarez B, Mangas C, Pérez-García V, Bueno C, Montes R, Ramos-Mejía V, Martínez-Camblor P, et al: **A promoter DNA demethylation landscape of human hematopoietic differentiation.** *Nucleic Acids Research* 2012, **40:**116-131.

57. Ramos-Mejía V, Fernandez A, Ayllon V, Real P, Bueno C, Anderson P, Martín F, Fraga M, Menendez P: **Maintenance of human embryonic stem cells in mesenchymal stem cell-conditioned media augments hematopoietic specification.** *Stem Cells Dev* 2012, **21:**1549-1558.

58. Reinius LE, Acevedo N, Joerink M, Pershagen G, Dahlén S-E, Greco D, Söderhäll C, Scheynius A, Kere J: **Differential DNA Methylation in Purified Human Blood Cells: Implications for Cell Lineage and Studies on Disease Susceptibility.** *PLoS ONE* 2012, **7:**e41361.

59. Sturm D, Witt H, Hovestadt V, Khuong-Quang D-A, Jones David TW, Konermann C, Pfaff E, Tönjes M, Sill M, Bender S, et al: **Hotspot Mutations in H3F3A and IDH1 Define Distinct Epigenetic and Biological Subgroups of Glioblastoma.** *Cancer Cell* 2012, **22:**425-437.

60. Fackler MJ, Umbricht CB, Williams D, Argani P, Cruz L-A, Merino VF, Teo WW, Zhang Z, Huang P, Visvananthan K, et al: **Genome-wide Methylation Analysis Identifies Genes Specific to Breast Cancer Hormone Receptor Status and Risk of Recurrence.** *Cancer Research* 2011, **71:**6195-6207.

61. Dedeurwaerder S, Desmedt C, Calonne E, Singhal SK, Haibe-Kains B, Defrance M, Michiels S, Volkmar M, Deplus R, Luciani J, et al: **DNA methylation profiling reveals a predominant immune component in breast cancers.** *EMBO Molecular Medicine* 2011, **3:**726-741.

62. Hinoue T, Weisenberger DJ, Lange CPE, Shen H, Byun H-M, Van Den Berg D, Malik S, Pan F, Noushmehr H, van Dijk CM, et al: **Genome-scale analysis of aberrant DNA methylation in colorectal cancer.** *Genome Res* 2012, **22:**271-282.

63. Lauss M, Aine M, Sjödahl G, Veerla S, Patschan O, Gudjonsson S, Chebil G, Lövgren K, Fernö M, Månsson W, et al: **DNA methylation analyses of urothelial carcinoma reveal distinct epigenetic subtypes and an association between gene copy number and methylation status.** *Epigenetics* 2012, **7:**858-867.

64. Weisenberger D, den Berg D, Pan F, Berman B, Laird P: **Comprehensive DNA methylation analysis on the Illumina Infinium assay platform.** *Technical report Illumina, Inc, San Diego* 2008.

65. Dunning M, Barbosa-Morais N, Lynch A, Tavare S, Ritchie M: **Statistical issues in the analysis of Illumina data.** *BMC Bioinformatics* 2008, **9:**85.

66. Dedeurwaerder S, Defrance M, Calonne E, Denis H, Sotiriou C, Fuks F: **Evaluation of the Infinium Methylation 450K technology.** *Epigenomics* 2011, **3:**771-784.

67. Maksimovic J, Gordon L, Oshlack A: **SWAN: Subset-quantile Within Array Normalization for Illumina Infinium HumanMethylation450 BeadChips.** *Genome Biology* 2012, **13:**R44.

68. Teschendorff AE, Marabita F, Lechner M, Bartlett T, Tegner J, Gomez-Cabrero D, Beck S: **A beta-mixture quantile normalization method for correcting probe design bias in Illumina Infinium 450 k DNA methylation data.** *Bioinformatics* 2013, **29:**189-196.

69. Ernst J, Kheradpour P, Mikkelsen TS, Shoresh N, Ward LD, Epstein CB, Zhang X, Wang L, Issner R, Coyne M, et al: **Mapping and analysis of chromatin state dynamics in nine human cell types.** *Nature* 2011, **473:**43-49.

70. Langfelder P, Mischel PS, Horvath S: **When is hub gene selection better than standard meta-analysis?** *PLoS ONE* 2013, **8:**e61505.

71. Göring H, Curran J, Johnson M, Dyer T, Charlesworth J, Cole S, Jowett J, Abraham L, Rainwater D, Comuzzie A, et al: **Discovery of expression QTLs using large-scale transcriptional profiling in human lymphocytes.** *Nat Genet* 2007, **39:**1208-1216.

72. Pankla R, Buddhisa S, Berry M, Blankenship DM, Bancroft GJ, Banchereau J, Lertmemongkolchai G, Chaussabel D: **Genomic transcriptional profiling identifies a candidate blood biomarker signature for the diagnosis of septicemic melioidosis.** *Genome Biol* 2009, **10:**R127.

73. Dumeaux V, Olsen KS, Nuel G, Paulssen RH, B√∏rresen-Dale A-L, Lund E: **Deciphering normal blood gene expression variation--the NOWAC postgenome study.** *PLoS Genet*, **6:**e1000873.

74. Cao J-N, Gollapudi S, Sharman EH, Jia Z, Gupta S: **Age-related alterations of gene expression patterns in human CD8+ T cells.** *Aging Cell* 2010, **9:**19-31.

75. Willinger T, Freeman T, Hasegawa H, McMichael AJ, Callan MFC: **Molecular Signatures Distinguish Human Central Memory from Effector Memory CD8 T Cell Subsets.** *The Journal of Immunology* 2005, **175:**5895-5903.

76. Willinger T, Freeman T, Herbert M, Hasegawa H, McMichael AJ, Callan MFC: **Human Naive CD8 T Cells Down-Regulate Expression of the WNT Pathway Transcription Factors Lymphoid Enhancer Binding Factor 1 and Transcription Factor 7 (T Cell Factor-1) following Antigen Encounter In Vitro and In Vivo.** *The Journal of Immunology* 2006, **176:**1439-1446.

77. Oldham M, Langfelder P, Horvath S: **Network methods for describing sample relationships in genomic datasets: application to Huntington's disease.** *BMC Syst Biol* 2012, **6:**63.

78. Lu T, Pan Y, Kao S-Y, Li C, Kohane I, Chan J, Yankner BA: **Gene regulation and DNA damage in the ageing human brain.** *Nature* 2004, **429:**883-891.

79. Myers AJ, Gibbs JR, Webster JA, Rohrer K, Zhao A, Marlowe L, Kaleem M, Leung D, Bryden L, Nath P, et al: **A survey of genetic human cortical gene expression.** *Nat Genet* 2007, **39:**1494-1499.

80. Oldham M, Konopka G, Iwamoto K, Langfelder P, Kato T, Horvath S, Geschwind D: **Functional organization of the transcriptome in human brain. .** *Nature Neuroscience* 2008, **11:**1271-1282.

81. Rodwell GE, Sonu R, Zahn JM, Lund J, Wilhelmy J, Wang L, Xiao W, Mindrinos M, Crane E, Segal E, et al: **A transcriptional profile of aging in the human kidney.** *PLoS Biol* 2004, **2:**e427.

82. Zahn J, Sonu R, Vogel H, Crane E, Mazan-Mamczarz K, Rabkin R, Davis R, Becker K, Owen A, Kim S: **Transcriptional profiling of aging in human muscle reveals a common aging signature.** *PLoS Genet* 2006, **2:**e115.

83. Warner HR: **The Future of Aging Interventions.** *The Journals of Gerontology Series A: Biological Sciences and Medical Sciences* 2004, **59:**B692-B696.

84. Johnson T: **Recent results: Biomarkers of aging.** *Experimental Gerontology* 2006, **41:**1243-1246.

85. Mather KA, Jorm AF, Parslow RA, Christensen H: **Is Telomere Length a Biomarker of Aging? A Review.** *The Journals of Gerontology Series A: Biological Sciences and Medical Sciences* 2011, **66A:**202-213.

86. Baker G, Sprott R: **Biomarkers of aging.** *Exp Gerontol* 1988, **23:**223-239.
