# Supplementary figures and images for: DNA methylation age of human tissues and cell types
Source: Genome Biol. 2013 Oct 21;14(10):R115. doi: 10.1186/gb-2013-14-10-r115 (PMC4015143; doi:10.1186/gb-2013-14-10-r115)

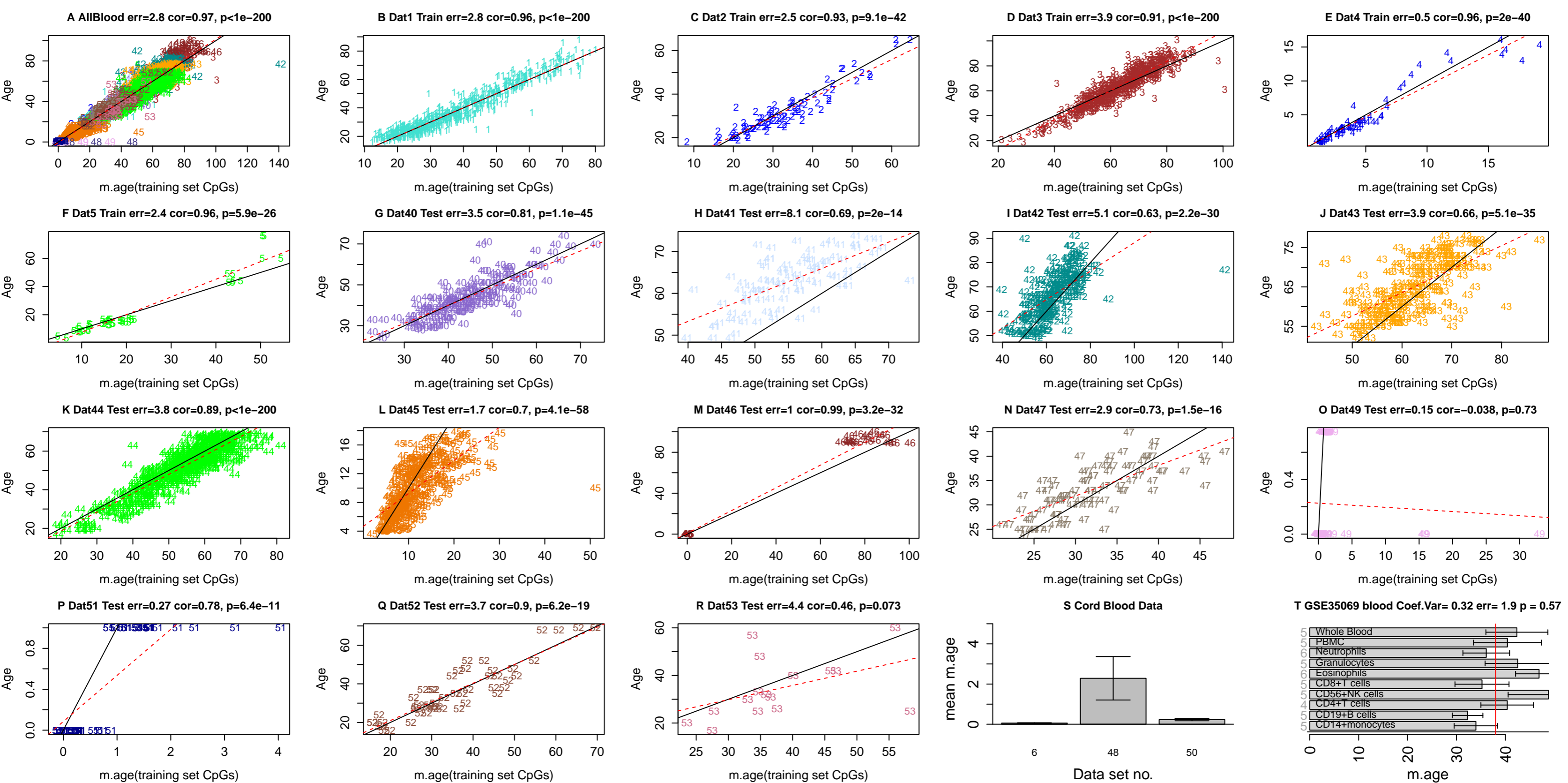

Supplement: Additional file 4 — Age predictions in blood data sets. (A) DNAm age has a high correlation with chronological age (y-axis) across all blood data sets. (B-S) Results for individual blood data sets. The negligible age correlation in panel 0) reflects very young subjects that were either zero or 0.75 years (9 months) old. (S) DNAm age in different cord blood data sets (x-axis). Bars report the mean DNAm age (±1 standard error). The mean DNAm age in data sets 6 and 50 is close to its expected value (zero) and it is not significantly different from zero in data set 48. (T) Mean DNAm age across whole blood, peripheral blood mononuclear cells, granulocytes as well as seven isolated cell populations (CD4+ T cells, CD8+ T cells, CD56+ natural killer cells, CD19+ B cells, CD14+ monocytes, neutrophils, and eosinophils) from healthy male subjects [82]. The red vertical line indicates the average age across subjects. No significant difference in DNAm age could be detected between these groups, but note the relatively small group sizes (indicated by the grey numbers on the y-axis). [file gb-2013-14-10-r115-S4.pdf]

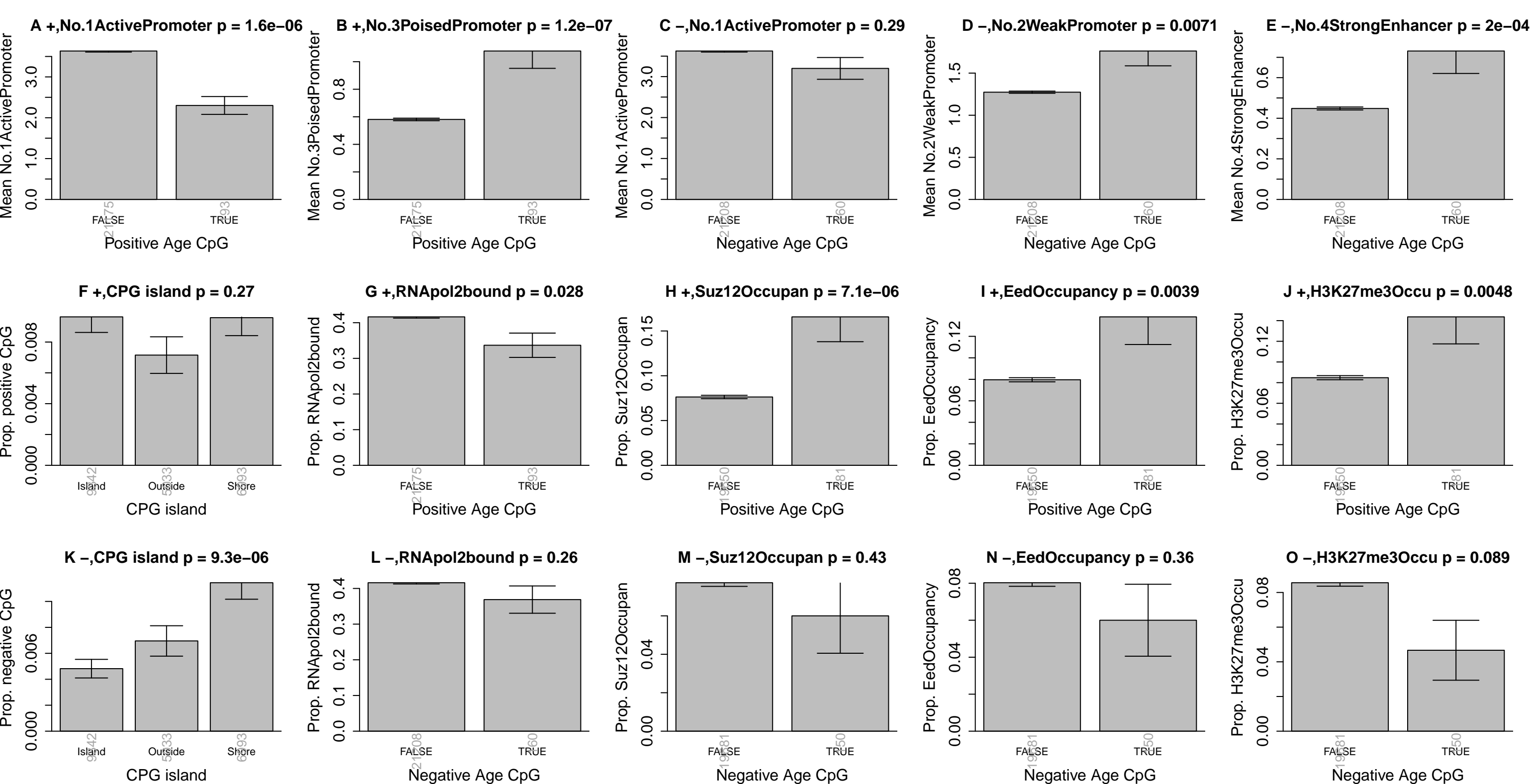

Supplement: Additional file 9 — Characterizing the clock CpGs using DNA sequence properties. Figure titles are preceded by ' + ’ or '-’ if they report properties of positively related or negatively related clock CpGs, respectively. Panels in the first row (A-E) relate the clock CpGs to chromatin state annotation provided in [29]. The y-axis reports the mean number of cell lines (out of 9 cell lines) for which the CpGs were in the chromatin state mentioned in the title. (A) The bar plots shows that the 193 positively related CpGs were significantly (P = 1.6E-6) less likely to be in chromatin state 1 (active promoters) than the other 21k CpGs, which is not the case for the 160 negatively related CpGs (C). (B) Positively related CpGs were more likely to be in chromatin state 3 regions (poised promoters). (D) Negatively related CpGs were more likely to be in chromatin states 2 (weak promoters). (E) Negatively related CpG are often located chromatin state 4 regions (strong enhancers). (F) No significant relationship with CpG island status can be observed for the positively related CpGs. (K) Negatively related CpGs are significantly over-represented in shores. (G) Positively related CpGs were outside of RNApol2 bound regions (annotation from [87]). This is not the case for negatively related CpGs (L). (H-J) Positively related CpGs are over-represented near Polycomb-group target genes, that is, in regions with high occupancy of Suz12 (P = 7.1E-6, H), EED (P = 0.0030, I), and H3K27m3 (P = 0.0048, J). This is not the case for the negatively related CpGs (M-O). [file gb-2013-14-10-r115-S9.pdf]

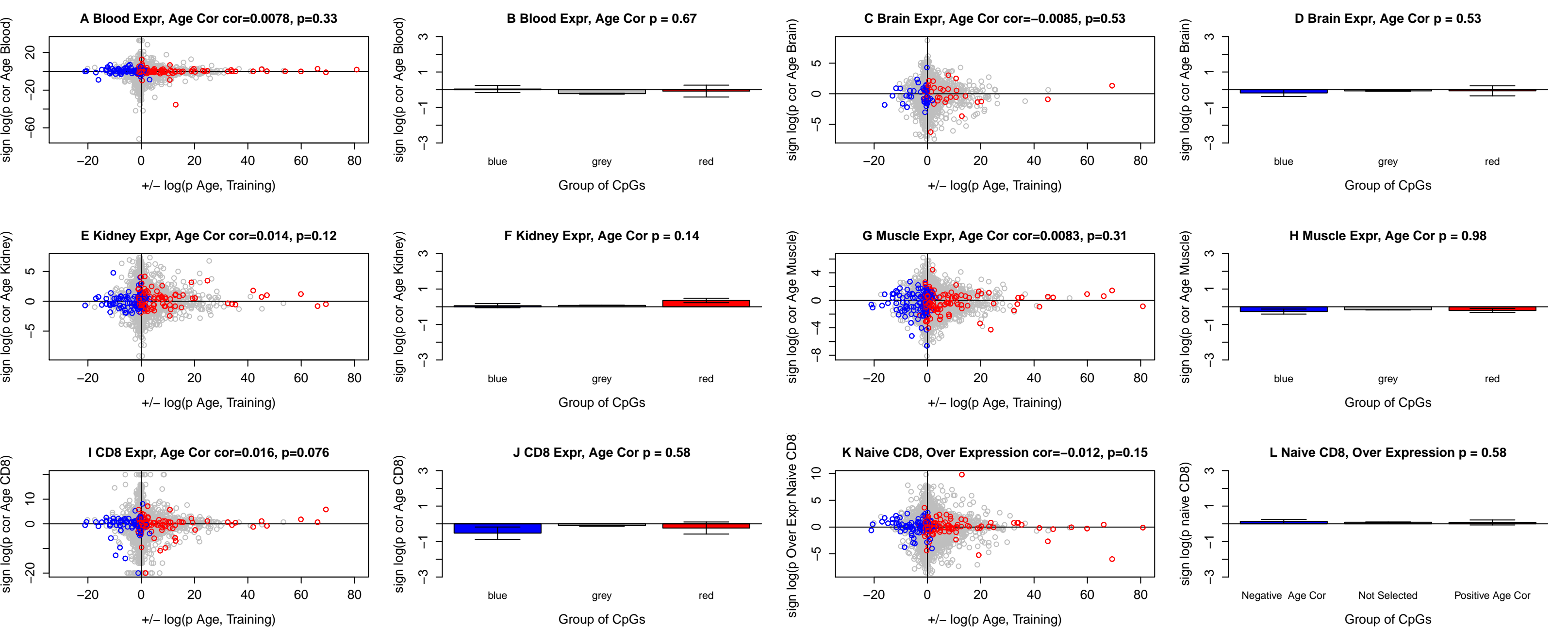

Supplement: Additional file 11 — Aging effects in gene expression (mRNA) and DNAm data. Due to space limitations, I can only report results for the direct approach of matching each individual CpG to its corresponding gene symbol. Using publicly available gene expression data (Additional file 2), I do not find a significant relationship between age effects on messenger RNA levels and age effects on DNAm levels in (A) blood, (C) brain, (E) kidney, (G) muscle, and (I) CD8 T cells. For each data modality, I estimated 'pure’ age effect using a meta-analysis method that conditioned on data (as described in Additional file 2). The y-axis reports a signed logarithm (base 10) of the meta-analysis P-value, that is, a high positive (negative) value indicates that the gene expression level increases (decreases) with age. Gene expression data and CpG data were matched according to gene symbol as described in [88]. Each point in the scatter plots corresponds to a CpG (x-axis) and the corresponding gene symbol (y-axis). Genes corresponding to the positively related and negatively related clock CpGs are colored in red and blue, respectively. (B,D,F,H,J,L) Mean age effect (y-axis) across gene groups defined by their corresponding CpG. (K,L) Aging effects on DNAm levels (x-axis) do not affect genes known to be differentially expressed between naive CD8 T cells and CD8 memory cells. The y-axis reports the signed logarithm of the Student t-test P-value of differential expression. [file gb-2013-14-10-r115-S11.pdf]

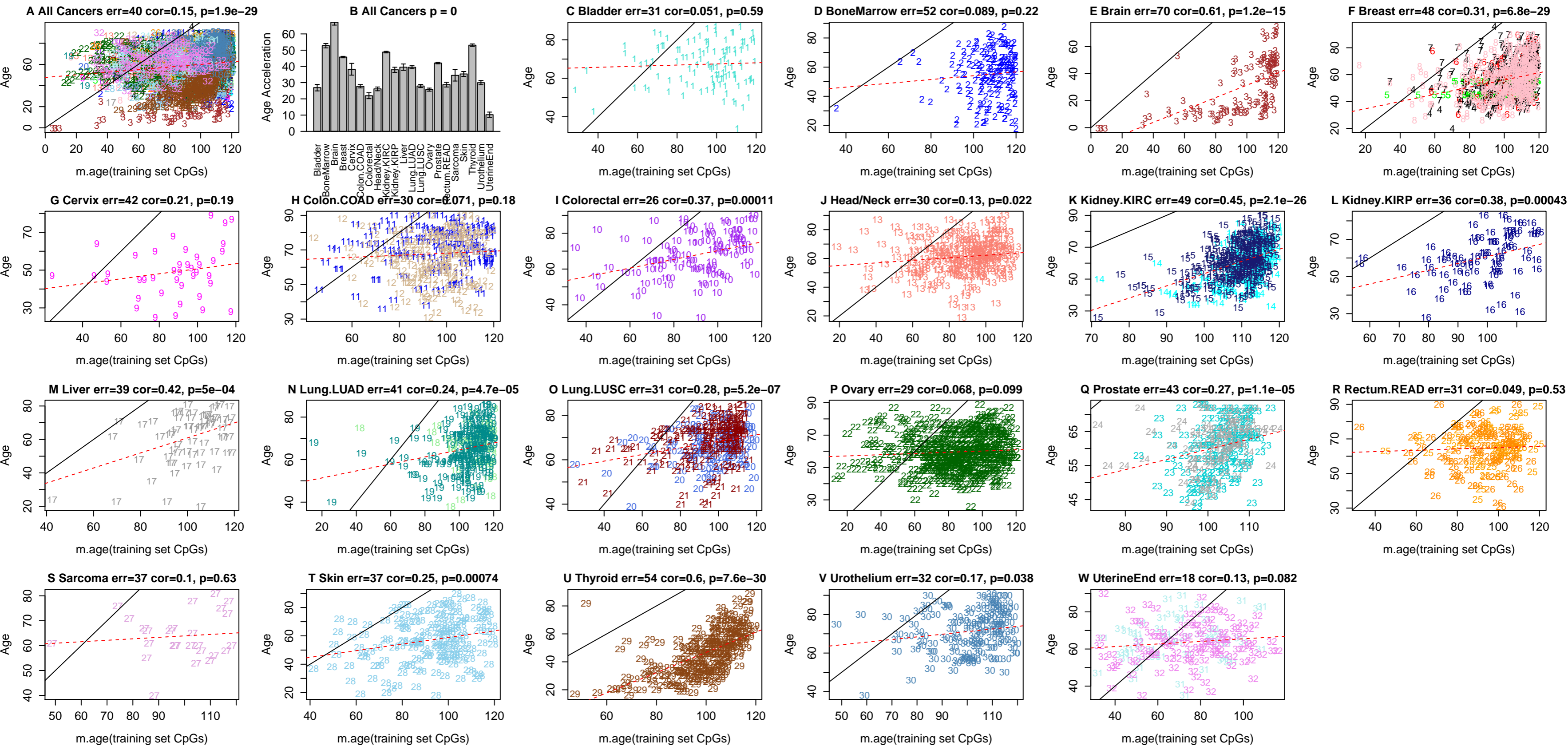

Supplement: Additional file 13 — DNAm age versus chronological age in cancer. Each point corresponds to a DNA methylation sample (cancer sample from a human subject). Points are colored and labeled according to the underlying cancer data sets as described in Additional file 12. (A) Across all cancer data sets, there is only a weak correlation (cor = 0.15, P = 1.9E-29) between DNAm age (x-axis) and chronological patient age (y-axis). The high error (40 years) reflects high age accelerations. (B) Each cancer/affected tissue shows evidence of significant age acceleration (y-axis) with an average age acceleration of 36.2 years. (C-W) Results for individual cancers/affected tissues. Several cancer tissues maintain moderately large age correlations (larger than 0.3), including brain (cor = 0.61) (E), thyroid (cor = 0.6) (U), kidney (cor = 0.45) (K,L), liver (cor = 0.42) (M), colorectal (cor = 0.37) (I), and breast (cor = 0.31) (F). [file gb-2013-14-10-r115-S13.pdf]

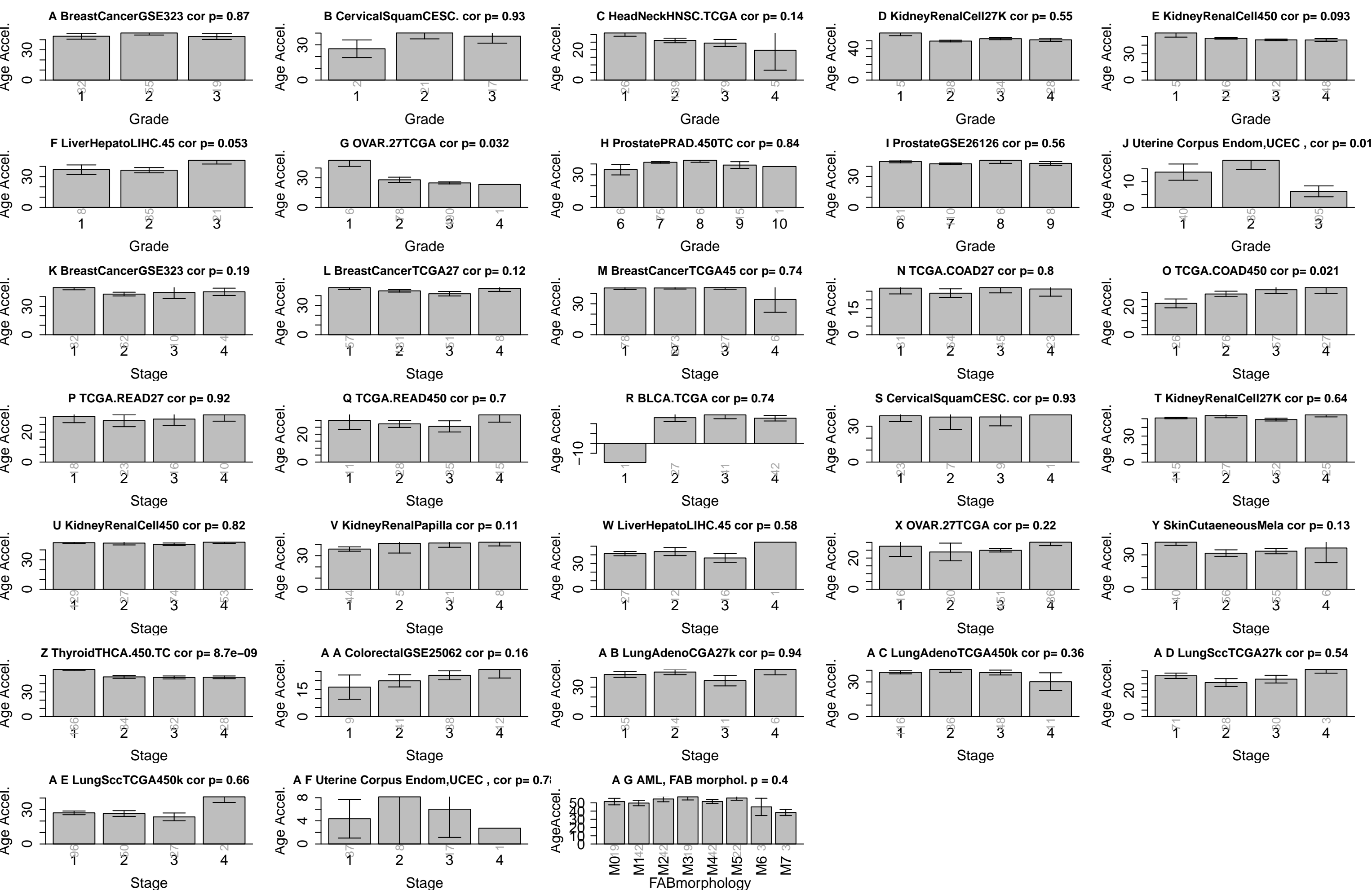

Supplement: Additional file 14 — Age acceleration versus tumor grade and stage. Panels correspond to the cancer data sets described in Additional file 12. Nominally significant negative correlations between grade and age acceleration can be observed in ovarian serous cystadenocarcinoma (panel G; P = 0.032) and uterine corpus endometroids (panel J; P = 0.019). A nominally significant positive correlation between stage and age acceleration can be observed for colon adenocarcinoma (panel O; P = 0.021). Only the highly significant negative correlation between stage and age acceleration in thyroid cancer (panel Z; P = 8.7E-9) remains significant after adjusting for multiple comparisons. Since grade and stage are often considered as ordinal variables, correlation test P-values are reported in all panels except the last. (H) For prostate cancer, the x-axis reports the Gleason sum score. The last panel shows that mean age acceleration in acute myeloid leukemia is not significantly related to French American British (FAB) morphology but some groups (notably M6 and M7) are very small (rotated grey numbers). [file gb-2013-14-10-r115-S14.pdf]

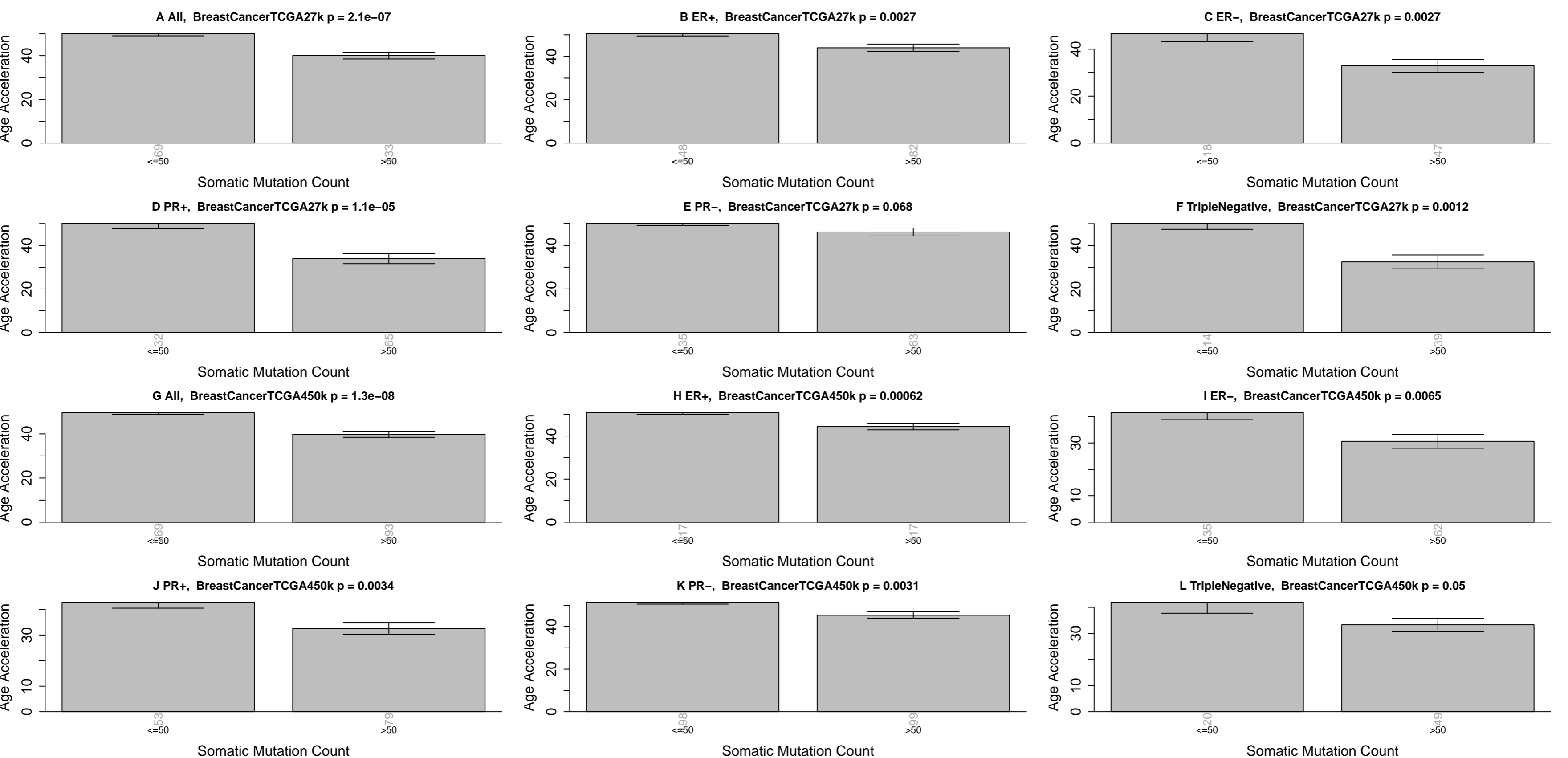

Supplement: Additional file 15 — Age acceleration versus mutation count status in breast cancer. Mutation count status (x-axis) was defined by assigning tumor samples to the high mutation count group if their number of somatic mutations was larger than 50. Other thresholds lead to similar results. (A-L) Findings for Illumina 27K (A-F) and 450K data (G-L). (A,G) The barplots show that mean age acceleration (y-axis) is lower in breast cancer samples with high mutation count (compared to those samples whose somatic mutation count is less than 50). This result can also be found in ER+ (B,H), ER- (C,I), PR + (D,J), PR- (E,K), and triple negative (F,L) breast cancer samples. [file gb-2013-14-10-r115-S15.pdf]

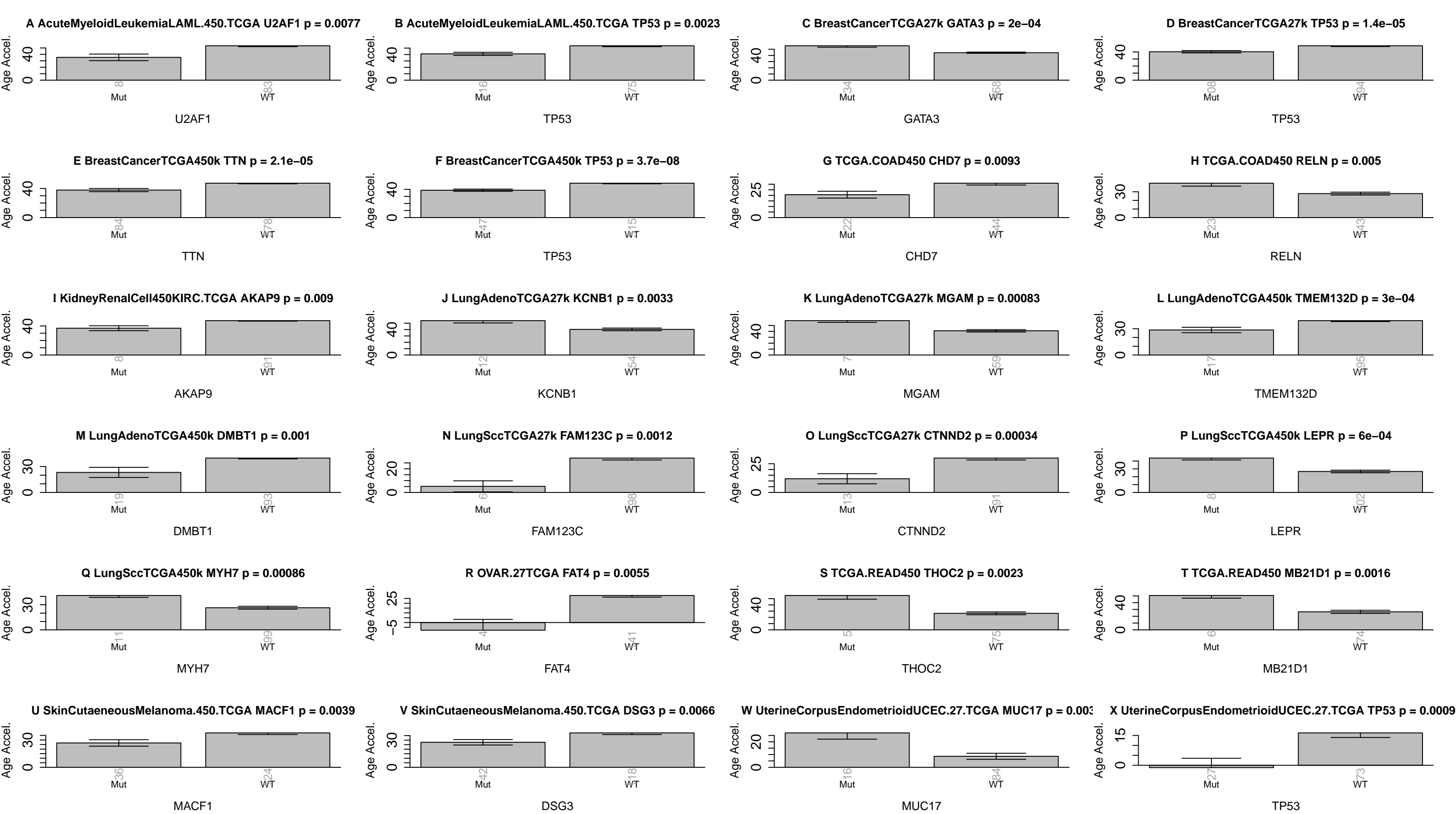

Supplement: Additional file 16 — Selected significant gene mutations versus age acceleration. The TCGA data sets were stratified by cancer type and Illumina platform. Mean age acceleration (y-axis) versus mutation status (x-axis) for up to two of the most significant genes per data set. Note that age acceleration in bone marrow (AML) was most highly related to mutation in the following two genes: U2AF1 and TP53. Age acceleration in the two breast cancer data sets was most highly related to mutations in GATA3, TP53, and TTN. For kidney renal cell carcinoma (KIRC): only AKAP9 was significant. Strikingly, TP53 was among the top two most significant mutated genes in 4 out of 13 cancer data sets. More information on these genes is presented in Additional file 2. [file gb-2013-14-10-r115-S16.pdf]

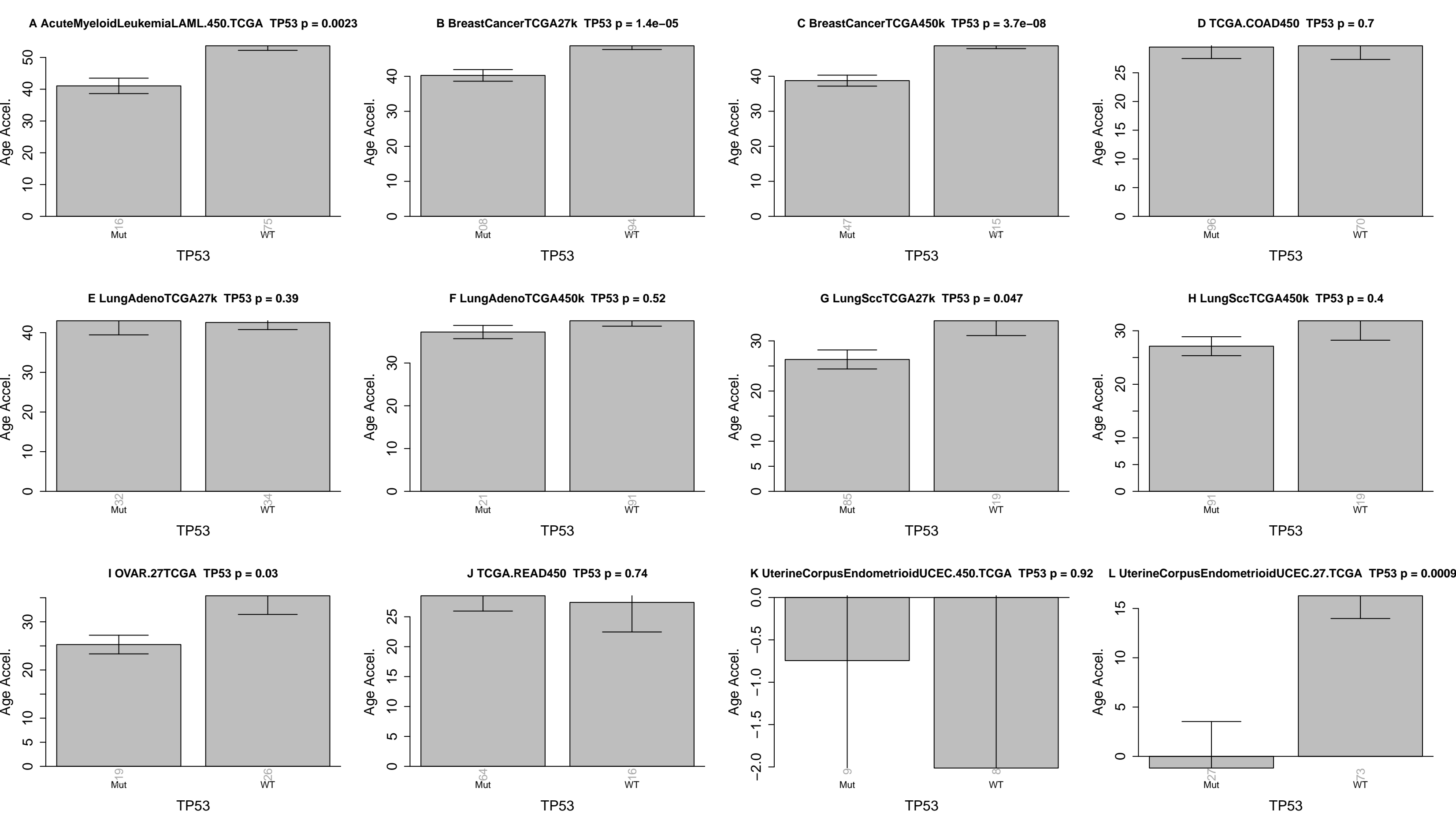

Supplement: Additional file 17 — Effect of TP53 mutation on age acceleration. Mutations in TP53 are associated with significantly lower age acceleration in five cancers: including AML (P = 0.0023), breast cancer (P = 1.4E-5 and P = 3.7E-8), ovarian serous cystadenocarcinoma (P = 0.03) (I), and uterine corpus endometrioid (P = 0.00093). Marginally significant results could be observed in lung squamous cell carcinoma (P = 0.047 for the 27K data but insignificant results for the 450K data). [file gb-2013-14-10-r115-S17.pdf]

A DNAmAge vs cell line

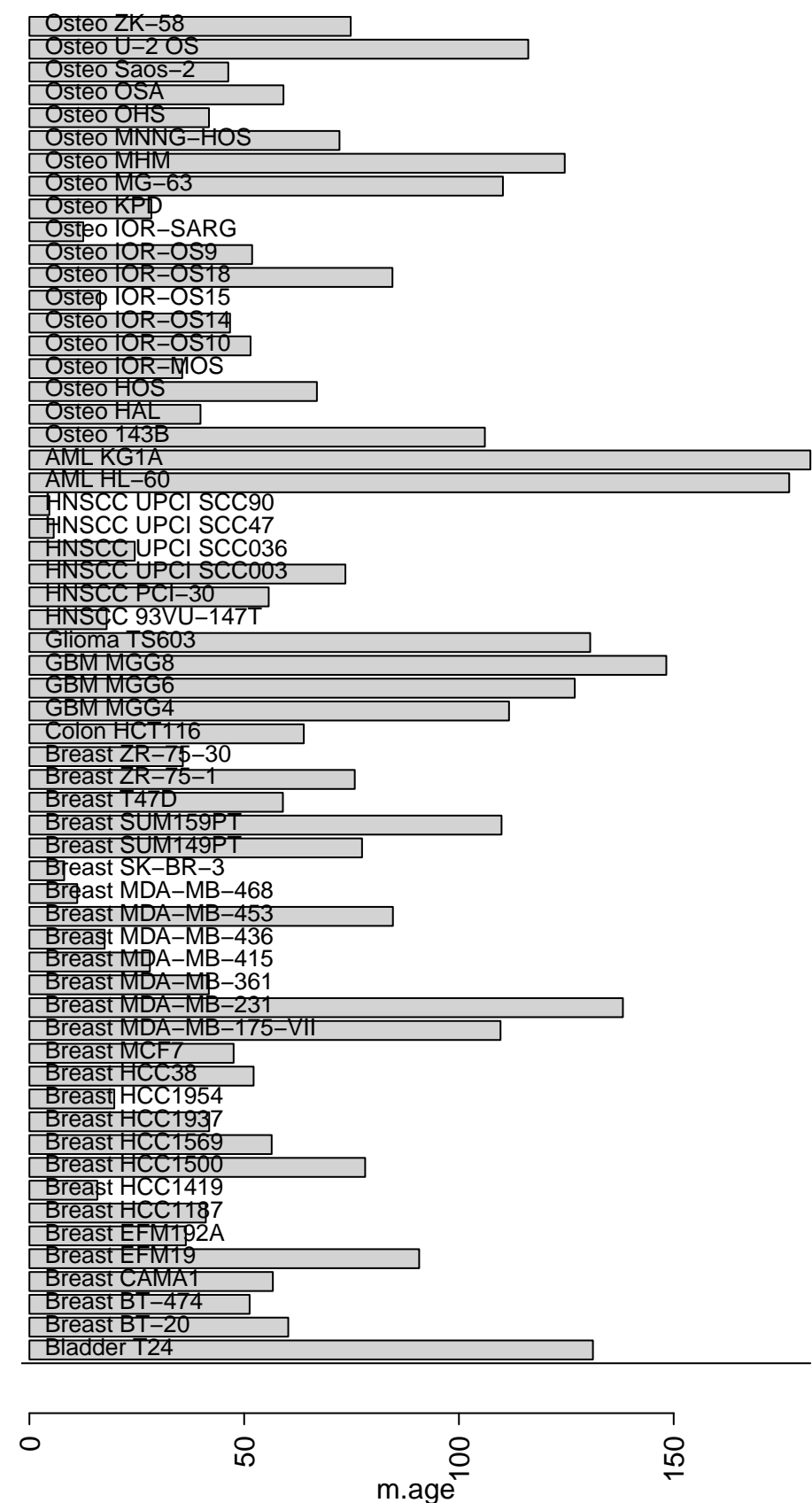

B Breast Cancer Cell Lines err=19 cor=0.15, p=0.47

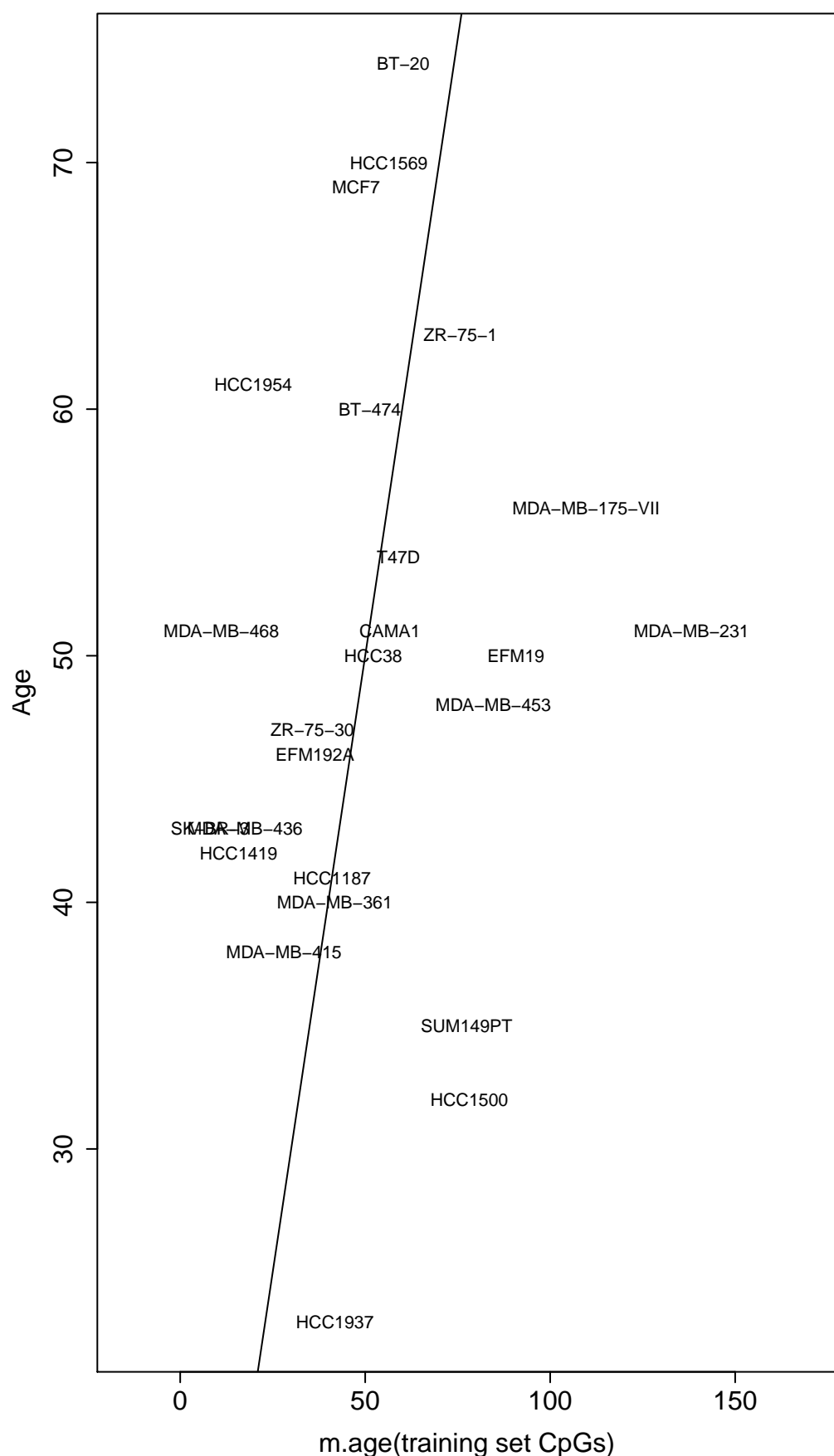

C Osteo Cancer Cell Lines err=40 cor=0.41, p=0.081

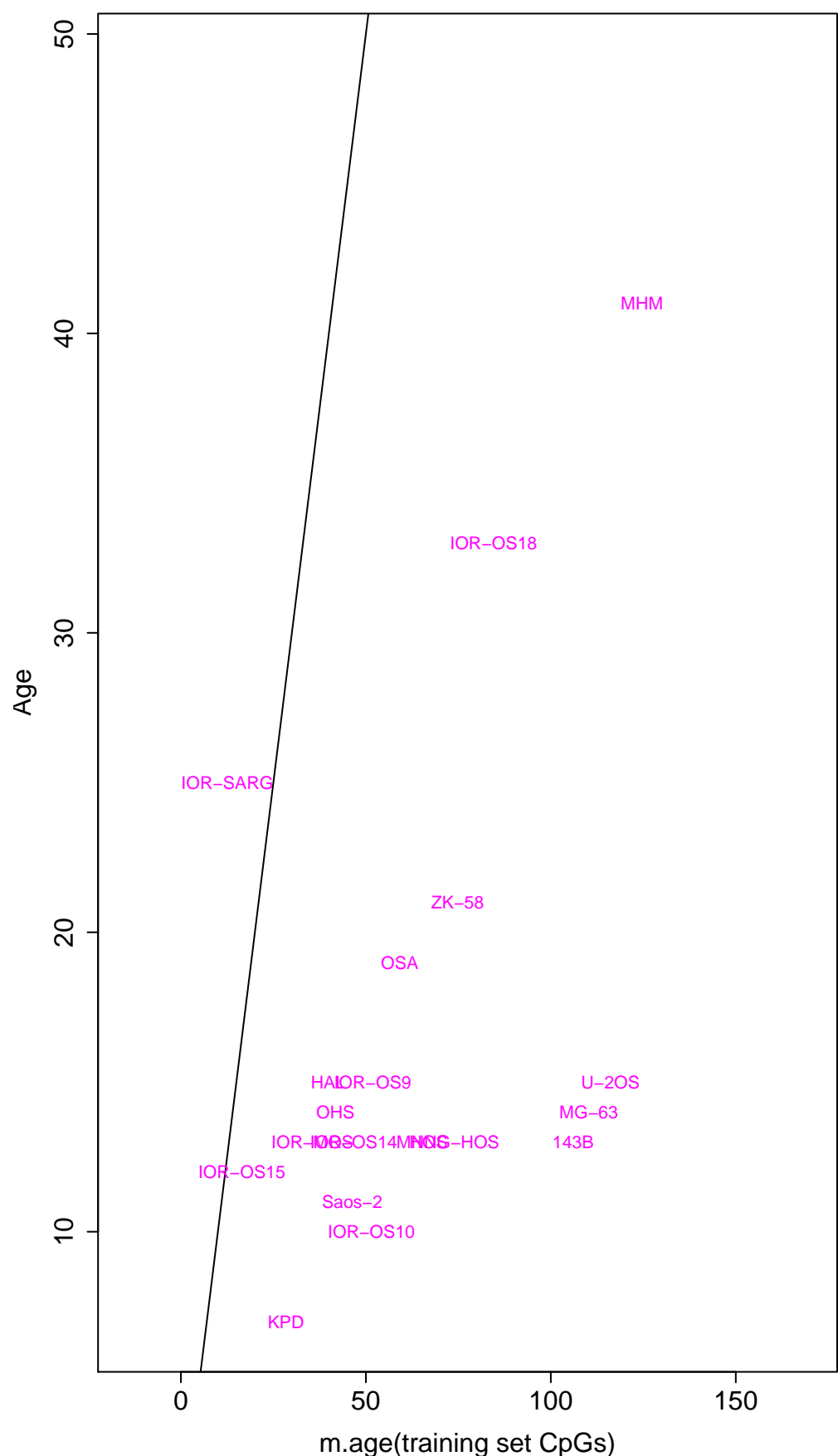

Supplement: Additional file 18 — DNAm age of cancer cell lines. (A) A high variation of DNAm age (x-axis) can be observed across various cancer lines lines (y-axis). The DNAm age is reported in Additional file 19. (B) Across all cell lines, DNAm age (x-axis) does not have a significant correlation with the chronological age of the patient from whom the cancer cell line was derived. (C) Results for osteosarcoma cell lines. [file gb-2013-14-10-r115-S18.pdf]
