# Supplementary material for: DNA methylation age of human tissues and cell types
Source: Genome Biol. 2013 Oct 21;14(10):R115. doi: 10.1186/gb-2013-14-10-r115 (PMC4015143; doi:10.1186/gb-2013-14-10-r115)
Supplement: Additional file 5 — Age predictions in brain data sets. (A) Scatter plot showing that DNAm age (defined using the training set CpGs) has a high correlation (cor = 0.96, error = 3.2 years) with chronological age (y-axis) across all training and test data sets. (B-J) Results in individual brain data sets. (G) The brain samples of data set 12 are composed of 58 glial cell (labeled G, blue color), 58 neuron cell (labeled N, red color), 20 bulk (labeled B, turquoise), and 9 mixed samples (labeled M, brown). (K) Comparison of mean DNAm ages (horizontal bars) across different brain regions from the same subjects [48] reveals no significant difference between temporal cortex, pons, frontal cortex, and cerebellum. Differing group sizes (grey numbers on the y-axis) reflect that some suspicious samples were removed in an unbiased fashion (Additional file 2). (L) Using data sets 54 and 55, I found no significant difference in DNAm age (x-axis) between cerebellum and occipital cortex from the same subjects [70]. [file gb-2013-14-10-r115-S5.pdf]

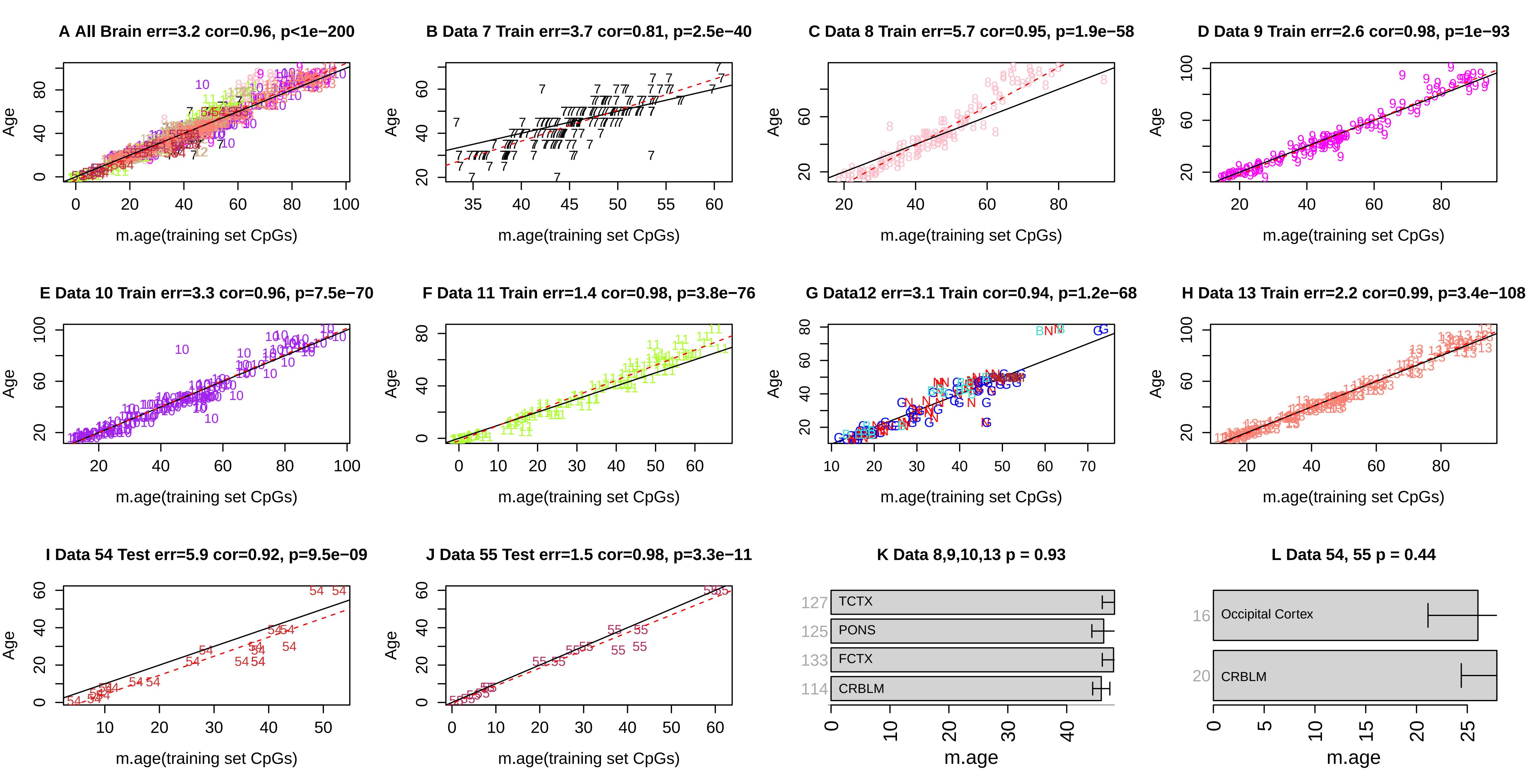
A All Brain err=3.2 cor=0.96, p<1e−200
B Data 7 Train err=3.7 cor=0.81, p=2.5e−40
C Data 8 Train err=5.7 cor=0.95, p=1.9e−58
D Data 9 Train err=2.6 cor=0.98, p=1e−93
E Data 10 Train err=3.3 cor=0.96, p=7.5e−70
F Data 11 Train err=1.4 cor=0.98, p=3.8e−76
G Data12 err=3.1 Train cor=0.94, p=1.2e−68
H Data 13 Train err=2.2 cor=0.99, p=3.4e−108
I Data 54 Test err=5.9 cor=0.92, p=9.5e−09
J Data 55 Test err=1.5 cor=0.98, p=3.3e−11
K Data 8,9,10,13 p = 0.93
L Data 54, 55 p = 0.44
Age
m.age(training set CpGs)
127 TCTX
125 PONS
133 FCTX
114 CRBLM
16 Occipital Cortex
20 CRBLM
m.age
