# Supplementary material for: DNA methylation age of human tissues and cell types
Source: Genome Biol. 2013 Oct 21;14(10):R115. doi: 10.1186/gb-2013-14-10-r115 (PMC4015143; doi:10.1186/gb-2013-14-10-r115)
Supplement: Additional file 6 — Age predictions in breast data sets. (A) DNAm age is highly correlated with age across all breast data sets, but the high error of 12 years reflects accelerated aging in normal adjacent breast cancer tissue (data sets 56, 57). (B-D) Relationship between DNAm age and chronological age in individual data sets. As expected, the lowest error (8.9 years) is observed in normal breast tissue (training data set 14, panel (B)). [file gb-2013-14-10-r115-S6.pdf]

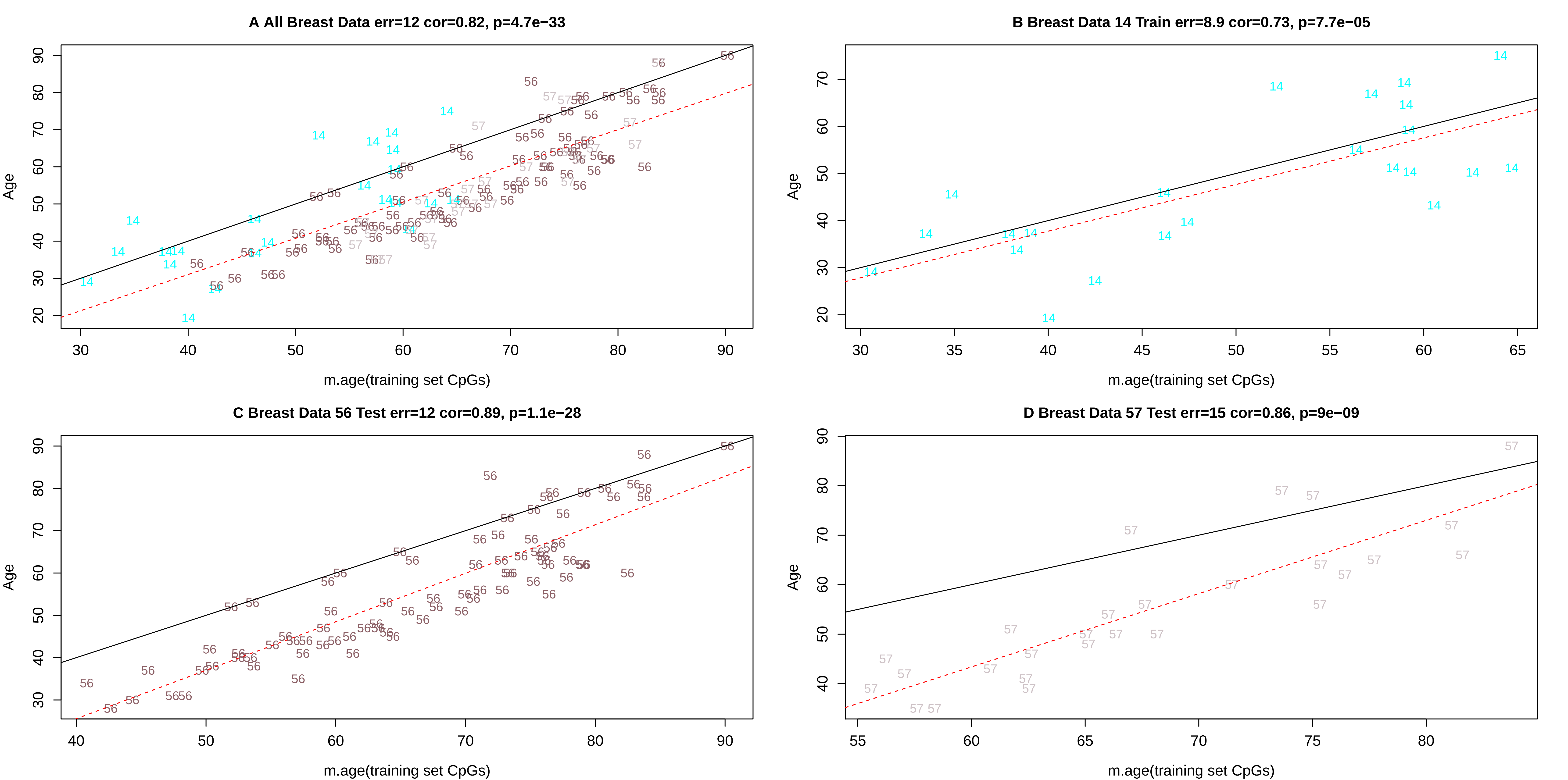
A All Breast Data err=12 cor=0.82, p=4.7e−33
Age
m.age(training set CpGs)
B Breast Data 14 Train err=8.9 cor=0.73, p=7.7e−05
Age
m.age(training set CpGs)
C Breast Data 56 Test err=12 cor=0.89, p=1.1e−28
Age
m.age(training set CpGs)
D Breast Data 57 Test err=15 cor=0.86, p=9e−09
Age
m.age(training set CpGs)
