# Supplementary material for: DNA methylation age of human tissues and cell types
Source: Genome Biol. 2013 Oct 21;14(10):R115. doi: 10.1186/gb-2013-14-10-r115 (PMC4015143; doi:10.1186/gb-2013-14-10-r115)

Additional File 7:
Ingenuity Pathway Analysis for the CpGs that are used for calculating DNA methylation age.

Horvath S. DNA Methylation age of human tissues and cell types.

Top Bio Functions

Diseases and Disorders

Name	p-value	# Molecules
Cancer	9.20E-05 - 3.17E-02	109
Developmental Disorder	2.55E-04 - 3.17E-02	28
Endocrine System Disorders	2.55E-04 - 3.17E-02	22
Hereditary Disorder	2.55E-04 - 3.17E-02	50
Reproductive System Disease	2.55E-04 - 3.12E-02	37

Molecular and Cellular Functions

Name	p-value	# Molecules
Cell Death and Survival	1.07E-07 - 3.17E-02	74
Cellular Growth and Proliferation	3.65E-05 - 3.17E-02	71
Lipid Metabolism	2.13E-04 - 3.17E-02	35
Molecular Transport	2.13E-04 - 3.17E-02	35
Small Molecule Biochemistry	2.13E-04 - 3.17E-02	44

Physiological System Development and Function

Name	p-value	# Molecules
Immune Cell Trafficking	2.58E-04 - 2.64E-02	28
Hematological System Development and Function	2.90E-04 - 3.17E-02	57
Organismal Development	4.61E-04 - 3.17E-02	56
Embryonic Development	6.06E-04 - 3.17E-02	36
Tissue Development	6.06E-04 - 3.17E-02	27

Top Canonical Pathways

Name	p-value	Ratio
Sertoli Cell-Sertoli Cell Junction Signaling	7.71E-03	8/187 (0.043)
Death Receptor Signaling	1.38E-02	4/63 (0.063)
Dopamine-DARPP32 Feedback in cAMP Signaling	1.6E-02	7/167 (0.042)
D-mannose Degradation	1.6E-02	1/1 (1)
Circadian Rhythm Signaling	1.68E-02	3/36 (0.083)

Top Upstream Regulators

Upstream Regulator	p-value of overlap	Predicted Activation State
RHOA	2.83E-04	
PHC1	7.00E-04	
TNF	9.73E-04	
ZFYVE9	1.39E-03	
PI3K (family)	2.43E-03	

Top Networks

ID	Associated Network Functions	Score
1	Hematological System Development and Function, Tissue Morphology, Cell Death and Survival	37
2	Cellular Growth and Proliferation, Cell Signaling, Developmental Disorder	37
3	Hepatocellular Peroxisome Proliferation, Organismal Functions, Cellular Assembly and Organization	30
4	Cellular Development, Hematological System Development and Function, Hematopoiesis	19
5	Cardiovascular Disease, Hematological Disease, Cell-To-Cell Signaling and Interaction	19

Network 1 p=E-37

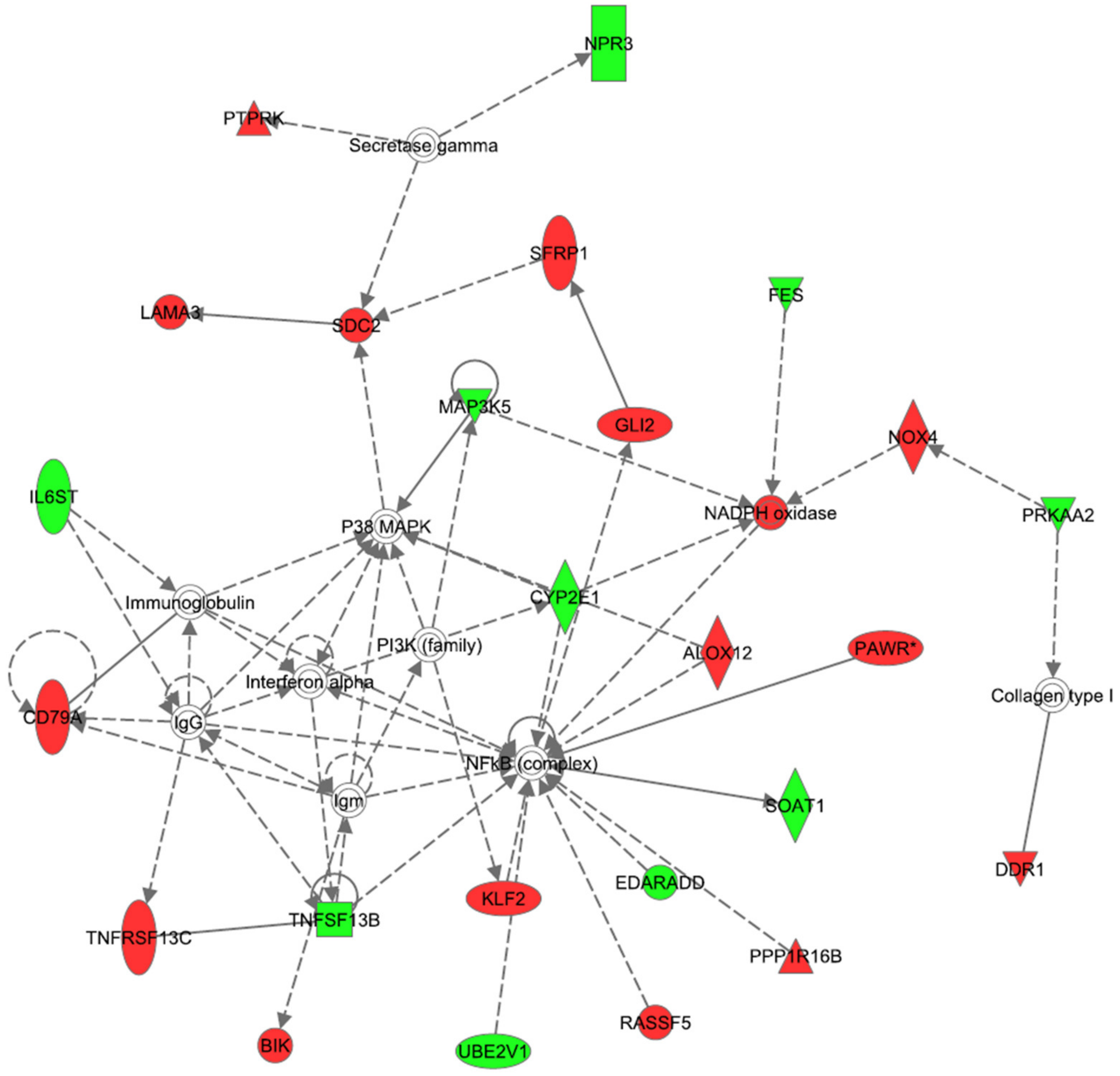

Genes colored in red (green) co-located with a CpG whose DNA methylation level positively (negatively) correlated with chronological age.

Network 2 p=E-37

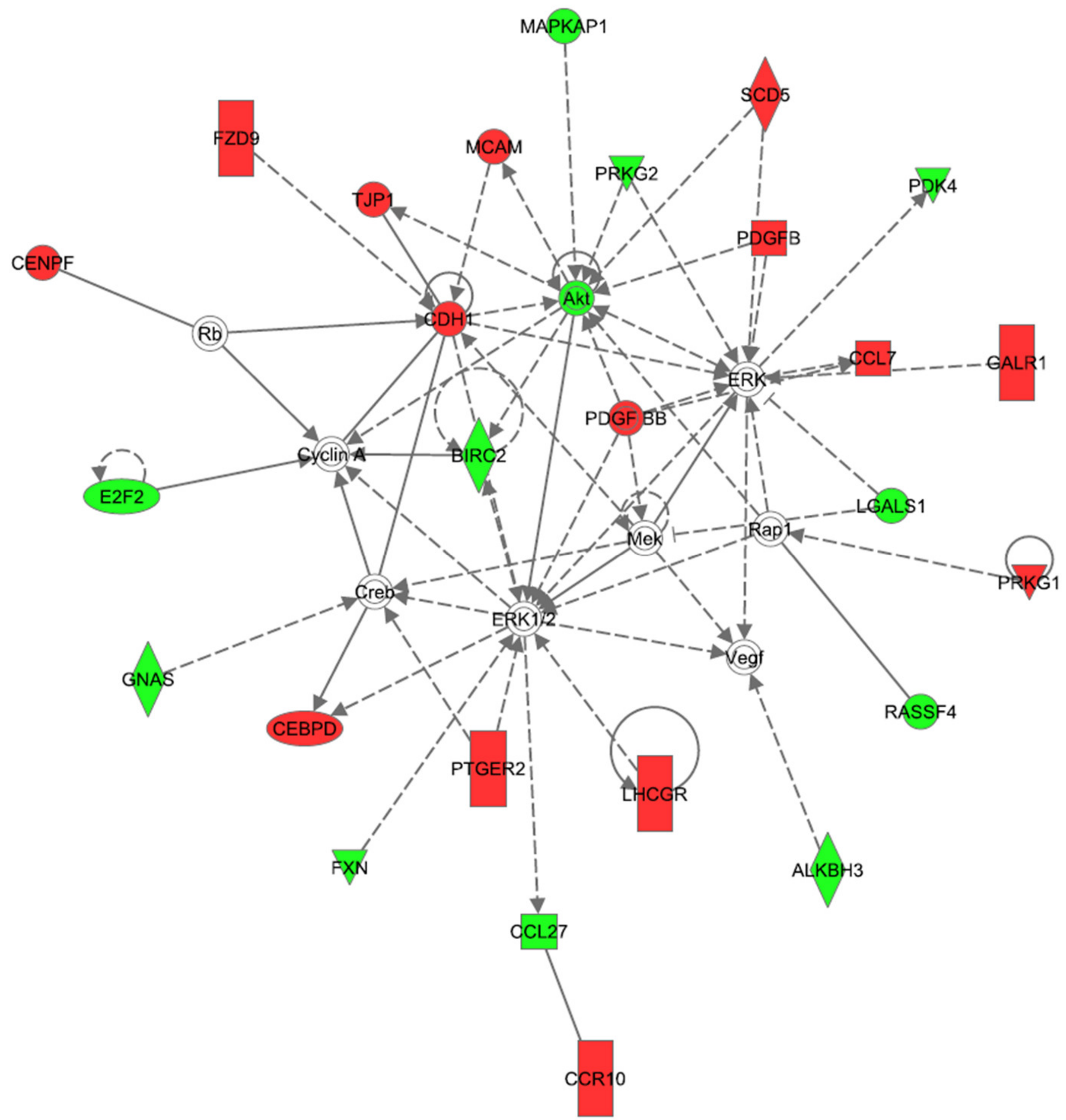

Genes colored in red (green) co-located with a CpG whose DNA methylation level positively (negatively) correlated with chronological age.

Network 3 p=E-30

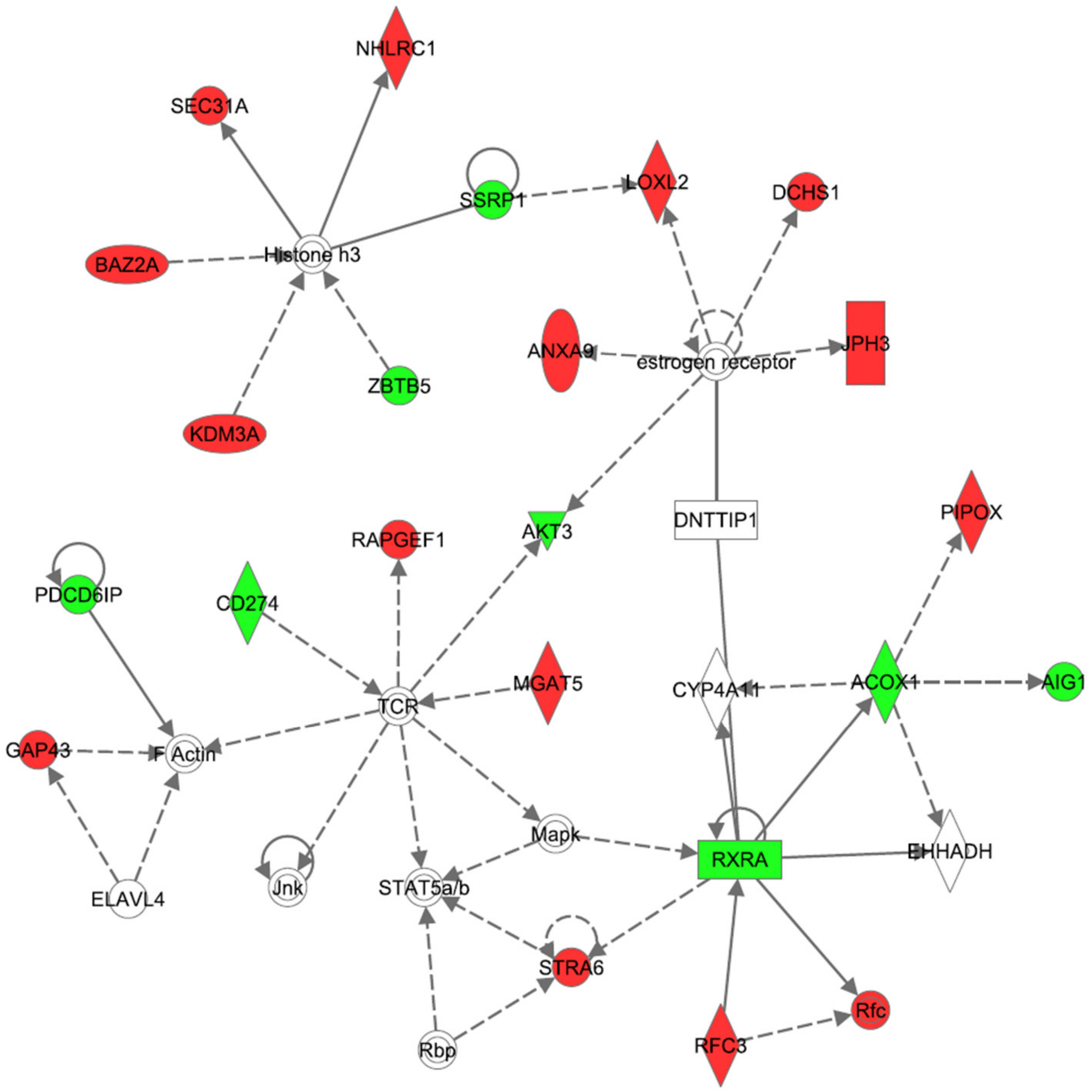

Genes colored in red (green) co-located with a CpG whose DNA methylation level positively (negatively) correlated with chronological age.

Network 4 p=E-19

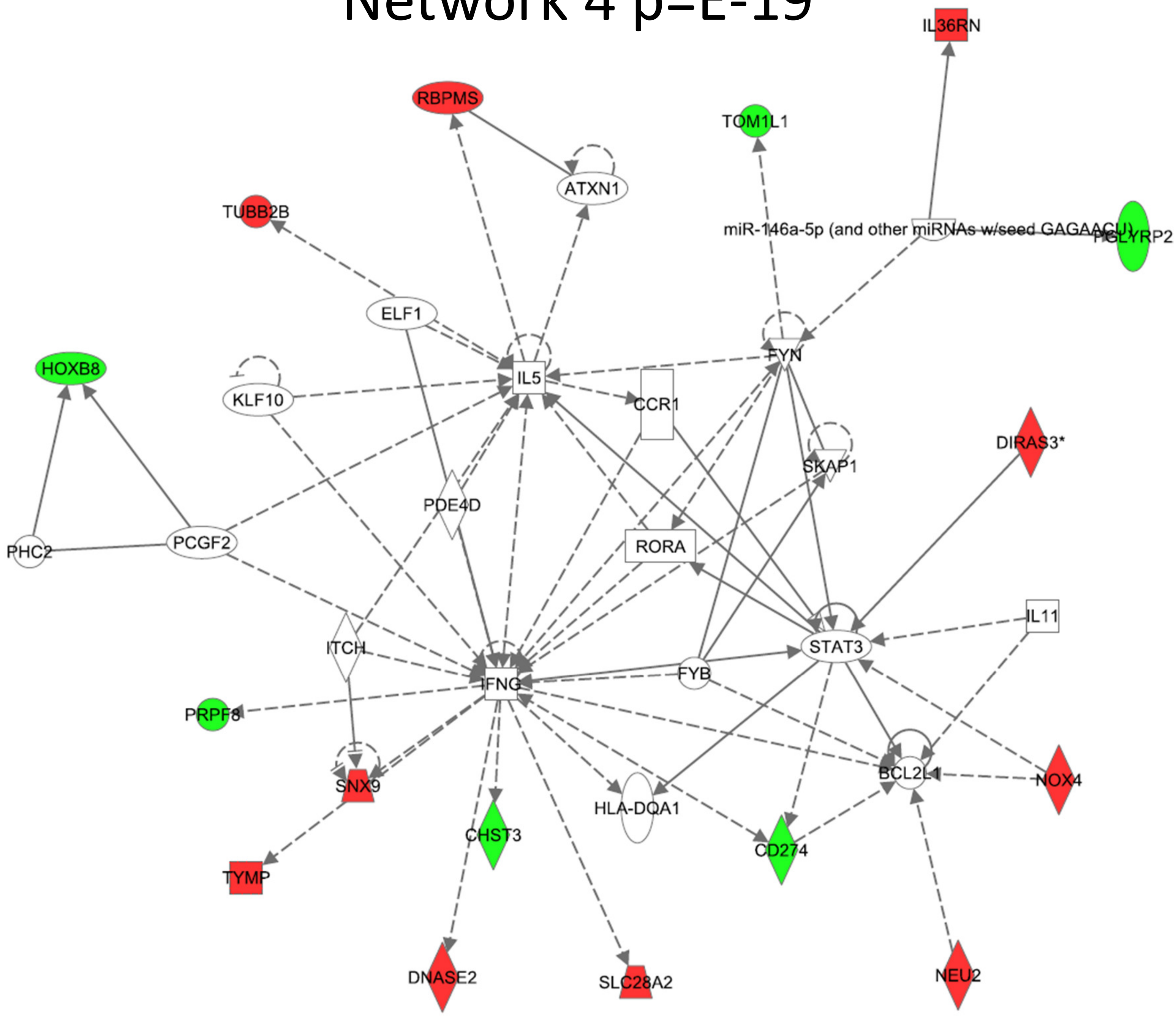

Network 5 p=E-19

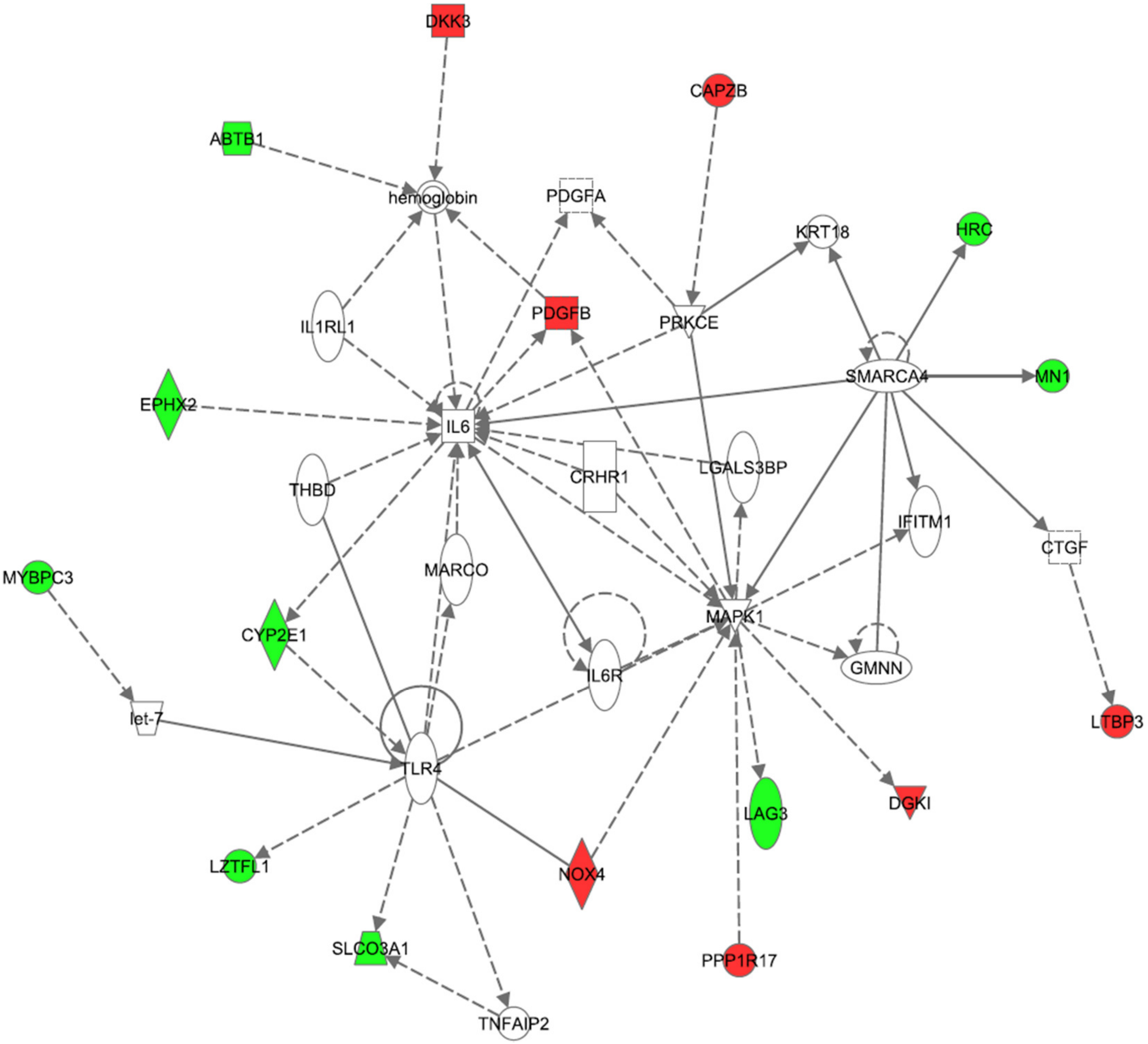

Supplement: Additional file 7 — Ingenuity Pathway Analysis. The document describes the results from applying Ingenuity Pathway Analysis to the 353 genes that are located near the 353 clock CpGs. Top biological function analysis implicated cell death/survival (74 genes, P = 1.1E-7) and cellular growth/development (71 genes, P = 3.7E-5). Significant overlap can be observed for the following disease-related gene sets: cancer (109 genes, P = 9.2E-5), endocrine system disorder (28, P = 2.6E-4), hereditary disorders (50 genes, 2.6E-4), and reproductive system disease (37 genes, P = 2.6E-4). Significant Ingenuity networks include a) hematological system development, tissue morphology, cell death and survival (P = E-37), b) cellular growth and proliferation, cell signaling, developmental disorder (P = E-37). [file gb-2013-14-10-r115-S7.pdf]
