# Supplementary material for: DNA methylation age of human tissues and cell types
Source: Genome Biol. 2013 Oct 21;14(10):R115. doi: 10.1186/gb-2013-14-10-r115 (PMC4015143; doi:10.1186/gb-2013-14-10-r115)
Supplement: Additional file 8 — Marginal analysis of CpGs. The figure shows how individual CpGs (corresponding to points) relate to age and tissue variation. Red and blue points correspond the 193 positively and the 160 negatively related clock CpGs, respectively. (A) The variance across adult somatic tissues is highly correlated with variance across fetal somatic tissues, which illustrates that it is robustly defined. Note that data set 77 [78] was not used for defining DNAm age. (B,C) Average variance of DNAm levels across adult and fetal somatic tissues, respectively. The blue and red bars correspond to groups of positively and negatively related clock CpGs, respectively. (D) Tissue variance across the training data (F statistic from ANOVA) is highly correlated (cor = 0.73) with tissue variance across adult somatic tissues (data set 77), which illustrates that tissue variance is robustly defined. (E) Pure (unconfounded) age effects in the training data (x-axis) relate to those in all data sets (y-axis). To estimate pure age effects, I used a meta-analysis method that implicitly conditions on data set (Materials and methods; Additional file 2). The logarithm (base 10) of the meta-analysis P-value was multiplied by -1 or 1 so that high positive (negative) values indicate that the CpG is positively (negatively) correlated with age. The high correlation illustrates that little information is lost by focusing on the training data. Further, note that the most significantly positively (red dots) and negatively related CpGs (blue dots) are used in the epigenetic clock. (F) Tissue variance in the training data (y-axis) versus the signed logarithm of the meta-analysis P-value in the training data (x-axis). [file gb-2013-14-10-r115-S8.pdf]

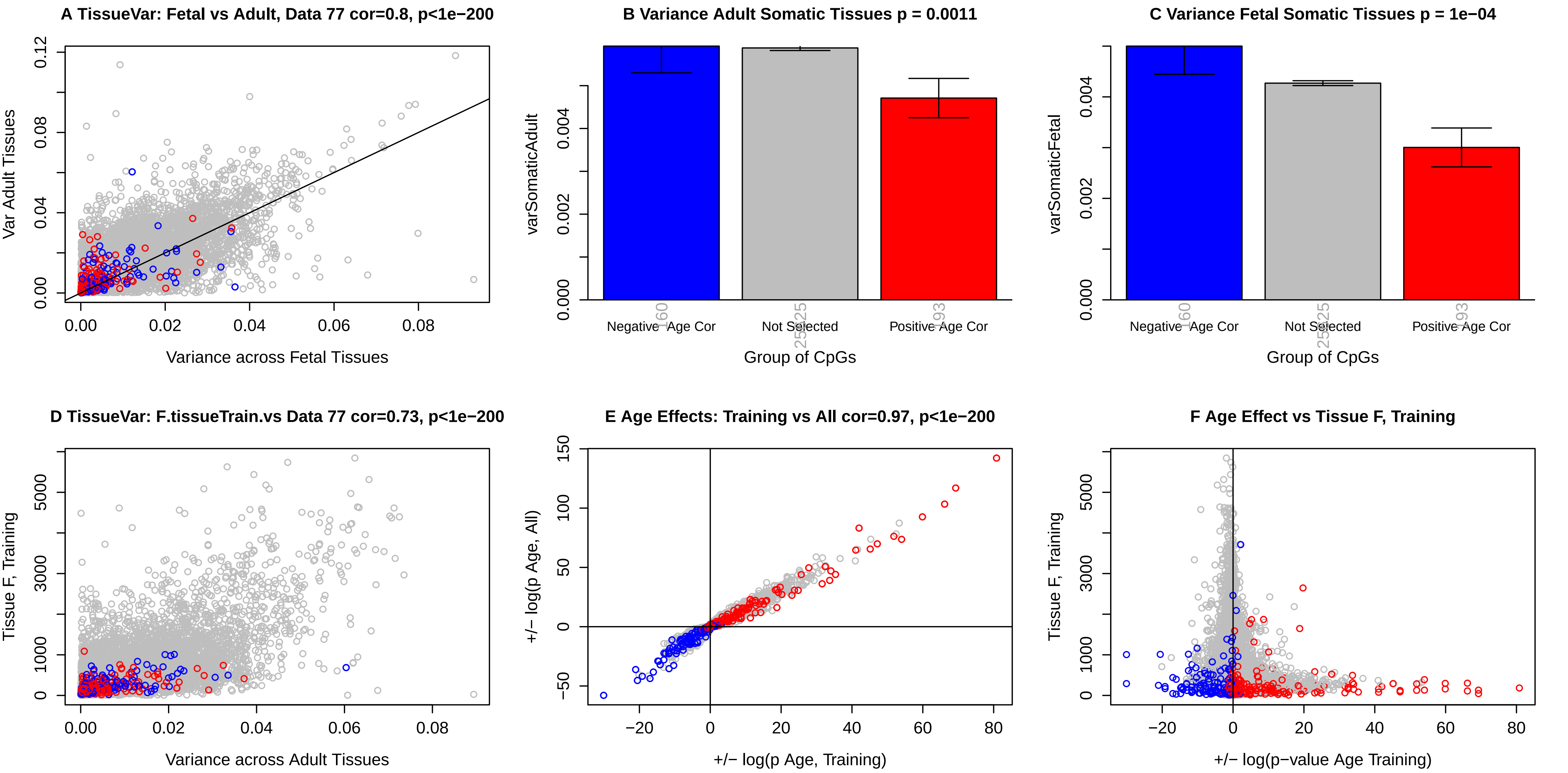
A TissueVar: Fetal vs Adult, Data 77 cor=0.8, p<1e−200
Var Adult Tissues
Variance across Fetal Tissues
0.00
0.04
0.08
0.12
0.00
0.02
0.04
0.06
0.08
B Variance Adult Somatic Tissues p = 0.0011
varSomaticAdult
0.000
0.002
0.004
160
25625
193
Negative Age Cor
Not Selected
Positive Age Cor
Group of CpGs
C Variance Fetal Somatic Tissues p = 1e−04
varSomaticFetal
0.000
0.002
0.004
160
25625
193
Negative Age Cor
Not Selected
Positive Age Cor
Group of CpGs
D TissueVar: F.tissueTrain.vs Data 77 cor=0.73, p<1e−200
Tissue F, Training
Variance across Adult Tissues
0
1000
3000
5000
0.00
0.02
0.04
0.06
0.08
E Age Effects: Training vs All cor=0.97, p<1e−200
+/− log(p Age, All)
+/− log(p Age, Training)
−50
0
50
100
150
−20
0
20
40
60
80
F Age Effect vs Tissue F, Training
Tissue F, Training
+/− log(p−value Age Training)
0
1000
3000
5000
−20
0
20
40
60
80
