# Supplementary material for: DNA methylation age of human tissues and cell types
Source: Genome Biol. 2013 Oct 21;14(10):R115. doi: 10.1186/gb-2013-14-10-r115 (PMC4015143; doi:10.1186/gb-2013-14-10-r115)
Supplement: Additional file 10 — Estimating the heritability of age acceleration. Two twin data sets (data sets 41 and 50) are used to estimate the broad sense heritability of accelerated age (defined as difference between DNAm age and chronological age). (A,E) Age histograms for data set 41 (median age 63 years, all females) and data set 50 (composed of newborns), respectively. (B,F) All twins irrespective of zygosity. Each point corresponds to a twin pair and is colored red if the twins are monozygotic. Age acceleration of the first twin (randomly chosen) versus that in the second twin, respectively. (C,G) Monozygotic twins only. (D,H) Dizygotic twins only. The high correlations in monozygotic twins (cor = 0.4 for data set 41 and cor = 0.77 for data set 50) contrast sharply with those observed for dizygotic twins (cor = 0.20 and cor = -0.21). [file gb-2013-14-10-r115-S10.pdf]

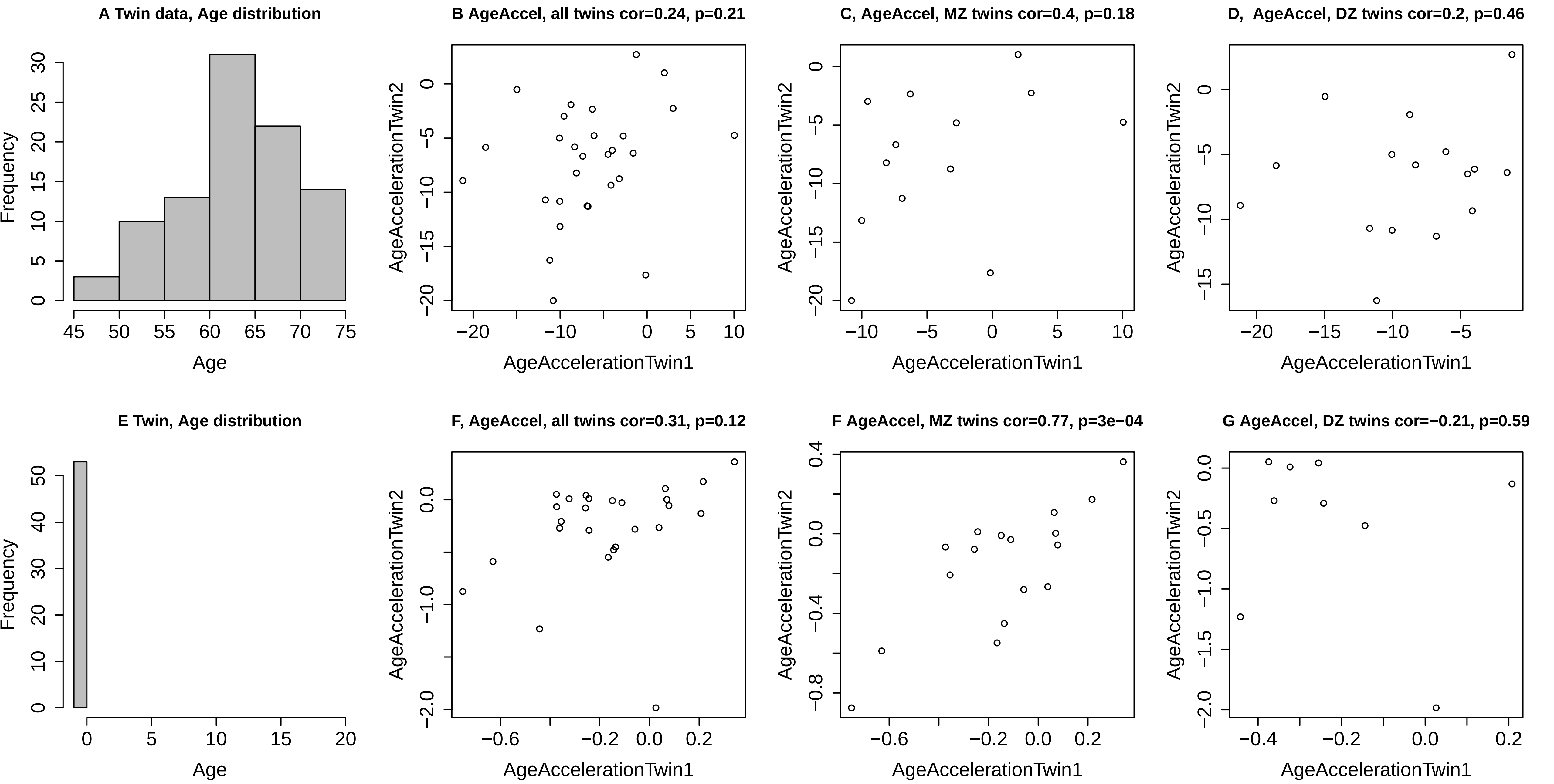
A Twin data, Age distribution
Frequency
Age
B AgeAccel, all twins cor=0.24, p=0.21
AgeAccelerationTwin2
AgeAccelerationTwin1
C, AgeAccel, MZ twins cor=0.4, p=0.18
AgeAccelerationTwin2
AgeAccelerationTwin1
D, AgeAccel, DZ twins cor=0.2, p=0.46
AgeAccelerationTwin2
AgeAccelerationTwin1
E Twin, Age distribution
Frequency
Age
F, AgeAccel, all twins cor=0.31, p=0.12
AgeAccelerationTwin2
AgeAccelerationTwin1
F AgeAccel, MZ twins cor=0.77, p=3e−04
AgeAccelerationTwin2
AgeAccelerationTwin1
G AgeAccel, DZ twins cor=−0.21, p=0.59
AgeAccelerationTwin2
AgeAccelerationTwin1
