# Supplementary material for: DNA methylation age of human tissues and cell types
Source: Genome Biol. 2013 Oct 21;14(10):R115. doi: 10.1186/gb-2013-14-10-r115 (PMC4015143; doi:10.1186/gb-2013-14-10-r115)
Supplement: Additional file 20 — R software tutorial. This file contains an R software tutorial that describes how to estimate DNAm age for data set 55. Further, it shows how to relate two measures of age acceleration to autism disease status. The R tutorial requires Additional files 21, 22, 23, 24, 25, 26 and 27 as input. [file gb-2013-14-10-r115-S20.docx]

**TUTORIAL 1**

**Estimate DNA methylation age with R**

**Steve Horvath (shorvath@mednet.ucla.edu)**

Abstract

This R software tutorial shows how to calculate DNA methylation age.

Input: A file with beta values, e.g. measured on the Illumina 27k or 450k platform.

Output: DMAm age.

To learn more details and to cite this method:

Horvath S (2013) DNA methylation age of human tissues and cell types. Genome Biology

Contents

[Instructions 1](#_Toc368640389)

[Example data set: 2](#_Toc368640390)

[Load files 2](#_Toc368640391)

[#Normalization step 2](#_Toc368640392)

[#Age transformation and probe annotation functions 3](#_Toc368640393)

[#Read in the DNA methylation data (beta values) 3](#_Toc368640394)

[Explanation of the output 4](#_Toc368640395)

[Where is the estimate of DNAmAge? 5](#_Toc368640396)

[Where are the normalized DNA methylation data? 5](#_Toc368640397)

[Relate DNAm age to chronological age 6](#_Toc368640398)

[Predict gender 6](#_Toc368640399)

[Does DNAm age acceleration relate to autism disease status, cause of death, postmortem interval? 6](#_Toc368640400)

# Instructions

Download the following files from the Supporting files of the article or

from the webpage: http://labs.genetics.ucla.edu/horvath/dnamage/

1. AdditionalFile21datMiniAnnotation27k.csv
2. AdditionalFile22probeAnnotation21kdatMethUsed.csv
3. AdditionalFile23predictor.csv
4. AdditionalFile24NORMALIZATION.R.txt
5. AdditionalFile25StepwiseAnalysis.txt

Save these files into the directory where your DNA meth data are located.

To run this tutorial, also download the following example data set from the webpage

1. AdditionalFile26MethylationDataExample55.csv
2. AdditionalFile27SampleAnnotationExample55.csv

Next download the freely available R software.

Next install R packages: RPMM, sqldf, impute(from Bioconductor) and the WGCNA.

Finally, copy and paste the R code listed below.

# Example data set:

In this tutorial, I will analyze data set 55:

- 16 men: autistic subjects and controls
- These are only the occipital cortex samples from the data set
- Illumina 27K platform
- GEO accession GSE38608
- Citation for the data set:

Ginsberg MR, Rubin RA, Falcone T, Ting AH et al. Brain transcriptional and epigenetic associations with autism. *PLoS One* 2012;7(9):e44736. PMID: [22984548](http://www.ncbi.nlm.nih.gov/pubmed/22984548)

# Load files

# Copy and pase the following R software code

# **Use forward slashes /”, as R will misread filepaths with backslashes**

setwd("C:/Users/SHorvath/dnamage/Example55")

library(WGCNA)

library(sqldf)

#install the Bioconductor installer

install.packages("BiocInstaller",repos="http://www.bioconductor.org/packages/2.13/bioc")

#install “impute” from Bioconductor

source("http://bioconductor.org/biocLite.R")

biocLite("impute")

# #Normalization step

# Comment regarding the following normalization method based on BMIQ.R

# The original BMIQ function from **Teschendorff 2013 (Bioinformatics. 2013 Jan 15;29(2):189-96)**

**#** adjusts for the type-2 bias in Illumina Infinium 450k data.

# Later functions and edits were provided by yours truly, Steve Horvath.

# I changed the code so that one can calibrate methylation data to a gold standard.

# Specifically, I took version v_1.2 by Teschendorff and fixed minor issues.

# Also I made the code more robust e.g. by changing the optimization algorithm.

# Toward this end, I used the method="Nelder-Mead" in optim()

source("AdditionalFile24NORMALIZATION.R.txt")

#Comment: The file AdditionalFile24NORMALIZATION.R.txt contains R function which will only be invoked in Step 3 below.

# #Age transformation and probe annotation functions

trafo= function(x,adult.age=20) { x=(x+1)/(1+adult.age); y=ifelse(x<=1, log( x),x-1);y }

anti.trafo= function(x,adult.age=20) { ifelse(x<0, (1+adult.age)*exp(x)-1, (1+adult.age)*x+adult.age) }

probeAnnotation21kdatMethUsed=read.csv("AdditionalFile22probeAnnotation21kdatMethUsed.csv")

probeAnnotation27k=read.csv("AdditionalFile21datMiniAnnotation27k.csv")

datClock=read.csv("AdditionalFile23predictor.csv")

# #Read in the DNA methylation data (beta values)

# For a small file, e.g. measured on the 27k platform you could just use read.csv.

# But for large files, e.g. those measured on the 450K platform, I recommend you use read.csv.sql.

dat0=read.csv.sql("AdditionalFile26MethylationDataExample55.csv") ;

nSamples=dim(dat0)[[2]]-1

nProbes= dim(dat0)[[1]]

# the following command may not be needed. But it is sometimes useful when you use read.csv.sql

dat0[,1]= gsub(x=dat0 [,1],pattern="\"",replacement="")

#Create a log file which will be output into your directory

# The code looks a bit complicated because it serves to create a log file (for error checks etc).

# It will automatically create a log file.

file.remove("LogFile.txt")

file.create("LogFile.txt")

DoNotProceed=FALSE

cat(paste( "The methylation data set contains", nSamples, "samples (e.g. arrays) and ", nProbes, " probes."),file="LogFile.txt")

if (nSamples==0) {DoNotProceed=TRUE; cat(paste( "\n ERROR: There must be a data input error since there seem to be no samples.\n Make sure that you input a comma delimited file (.csv file)\n that can be read using the R command read.csv.sql . Samples correspond to columns in that file ."), file="LogFile.txt",append=TRUE) }

if (nProbes==0) {DoNotProceed=TRUE; cat(paste( "\n ERROR: There must be a data input error since there seem to be zero probes.\n Make sure that you input a comma delimited file (.csv file)\n that can be read using the R command read.csv.sql CpGs correspond to rows.") , file="LogFile.txt",append=TRUE) }

if ( nSamples > nProbes ) { cat(paste( "\n MAJOR WARNING: It worries me a lot that there are more samples than CpG probes.\n Make sure that probes correspond to rows and samples to columns.\n I wonder whether you want to first transpose the data and then resubmit them? In any event, I will proceed with the analysis."),file="LogFile.txt",append=TRUE) }

if ( is.numeric(dat0[,1]) ) { DoNotProceed=TRUE; cat(paste( "\n Error: The first column does not seem to contain probe identifiers (cg numbers from Illumina) since these entries are numeric values. Make sure that the first column of the file contains probe identifiers such as cg00000292. Instead it contains ", dat0[1:3,1] ),file="LogFile.txt",append=TRUE) }

if ( !is.character(dat0[,1]) ) { cat(paste( "\n Major Warning: The first column does not seem to contain probe identifiers (cg numbers from Illumina) since these entries are numeric values. Make sure that the first column of the file contains CpG probe identifiers such as cg00000292. Instead it contains ", dat0[1:3,1] ),file="LogFile.txt",append=TRUE) }

datout=data.frame(Error=c("Input error. Please check the log file for details","Please read the instructions carefully."), Comment=c("", "email Steve Horvath."))

if ( ! DoNotProceed ) {

nonNumericColumn=rep(FALSE, dim(dat0)[[2]]-1)

for (i in 2:dim(dat0)[[2]] ){ nonNumericColumn[i-1]=! is.numeric(dat0[,i]) }

if ( sum(nonNumericColumn) >0 ) { cat(paste( "\n MAJOR WARNING: Possible input error. The following samples contain non-numeric beta values: ", colnames(dat0)[-1][ nonNumericColumn], "\n Hint: Maybe you use the wrong symbols for missing data. Make sure to code missing values as NA in the Excel file. To proceed, I will force the entries into numeric values but make sure this makes sense.\n" ),file="LogFile.txt",append=TRUE) }

XchromosomalCpGs=as.character(probeAnnotation27k$Name[probeAnnotation27k$Chr=="X"])

selectXchromosome=is.element(dat0[,1], XchromosomalCpGs )

selectXchromosome[is.na(selectXchromosome)]=FALSE

meanXchromosome=rep(NA, dim(dat0)[[2]]-1)

if ( sum(selectXchromosome) >=500 ) {

meanXchromosome= as.numeric(apply( as.matrix(dat0[selectXchromosome,-1]),2,mean,na.rm=TRUE)) }

if ( sum(is.na(meanXchromosome)) >0 ) { cat(paste( "\n \n Comment: There are lots of missing values for X chromosomal probes for some of the samples. This is not a problem when it comes to estimating age but I cannot predict the gender of these samples.\n " ),file="LogFile.txt",append=TRUE) }

match1=match(probeAnnotation21kdatMethUsed$Name , dat0[,1])

if ( sum( is.na(match1))>0 ) {
missingProbes= probeAnnotation21kdatMethUsed$Name[!is.element( probeAnnotation21kdatMethUsed$Name , dat0[,1])]

DoNotProceed=TRUE; cat(paste( "\n \n Input error: You forgot to include the following ", length(missingProbes), " CpG probes (or probe names):\n ", paste( missingProbes, sep="",collapse=", ")),file="LogFile.txt",append=TRUE) }

**#STEP 2: Restrict the data to 21k probes and ensure they are numeric**

match1=match(probeAnnotation21kdatMethUsed$Name , dat0[,1])

if ( sum( is.na(match1))>0 ) stop(paste(sum( is.na(match1)), "CpG probes cannot be matched"))

dat1= dat0[match1,]

asnumeric1=function(x) {as.numeric(as.character(x))}

dat1[,-1]=apply(as.matrix(dat1[,-1]),2,asnumeric1)

**#STEP 3: Create the output file called datout**

set.seed(1)

# Do you want to normalize the data (recommended)?

normalizeData=TRUE

source("AdditionalFile25StepwiseAnalysis.txt")

**# STEP 4: Output the results**

if ( sum( datout$Comment != "" ) ==0 ) { cat(paste( "\n The individual samples appear to be fine. "),file="LogFile.txt",append=TRUE) }

if ( sum( datout$Comment != "" ) >0 ) { cat(paste( "\n Warnings were generated for the following samples.\n", datout[,1][datout$Comment != ""], "\n Hint: Check the output file for more details."),file="LogFile.txt",append=TRUE) }

**}**

# output the results into the directory

write.table(datout,"Output.csv", row.names=F, sep="," )

# Explanation of the output

You can find it in the output file Output.csv in the directory. Note that this csv file contains a host of useful information e.g.

- SampleID=sample identifier
- DNAmAge=DNA methylation age=predicted age.
- Comment=I only add a comment if a sample looks suspicious.
- noMissingPerSample=number of missing beta values per sample,
- meanMethBySample, minMethBySample=the mean and min beta value before normalization
- predictedGender=predicted gender based on the mean across the X chromosomal markers.
- meanXchromosome= mean beta value across the X chromosomal markers.

## Where is the estimate of DNAmAge?

Answer: You can find it in the output file in the directory. Further, it is also given by the following

signif(datout$DNAmAge,2)

[1] 60.00 43.00 28.00 38.00 8.20 20.00 4.80 38.00 6.80 3.60 31.00 0.98 62.00 24.00 8.00 43.00

## Where are the normalized DNA methylation data?

Note that your R session contains a data frame called datMethUsedNormalized

whose rows correspond to the samples while the columns correspond to the 21368 CpGs that are used in the age prediction algorithm.

dim(datMethUsedNormalized)

16 21368

#Before outputting these normalized data, you may first want to transpose them and insert a probe identifier.

dat0UsedNormalized=data.frame(CpGName=colnames(datMethUsedNormalized), data.frame(t(datMethUsedNormalized) ))

#Here are the first few rows and columns

dat0UsedNormalized[1:5,1:5]

CpGName GSM946048 GSM946049 GSM946052 GSM946054

cg00000292 cg00000292 0.67564060 0.70119361 0.67919958 0.71834583

cg00002426 cg00002426 0.31489068 0.31818404 0.35098754 0.32295065

cg00003994 cg00003994 0.06801064 0.03221467 0.03634316 0.05610162

cg00005847 cg00005847 0.19903216 0.18919720 0.19286406 0.17821503

cg00007981 cg00007981 0.12023727 0.12412725 0.11628779 0.12483555

#Output the data to your directory

write.table(dat0UsedNormalized,file="dat0UsedNormalized.csv",sep=",",row.names=F)

## Relate DNAm age to chronological age

#To address this task, we read in the sample annotation data that contain the chronological ages.

datSample=read.csv("AdditionalFile27SampleAnnotationExample55.csv")

DNAmAge=datout$DNAmAge

medianAbsDev=function(x,y) median(abs(x-y),na.rm=TRUE)

medianAbsDev1=signif(medianAbsDev(DNAmAge, datSample$Age),2)

par(mfrow=c(1,1))

verboseScatterplot(DNAmAge, datSample$Age,xlab="DNAm Age", ylab="Chronological Age",main=paste("All, err=", medianAbsDev1) );abline(0,1)

**Caption:** **Chronological age (y-axis) is highly related with DNAm Age**. The low error (defined as median absolute deviation) shows that DNAm is well calibrated. This is a distinguishing feature of the multi-tissue age predictor.

## Predict gender

datout$predictedGender

[1] male male male male male male male male male male male male male male male male

This happens to agree with their known gender as can be seen from the following

table(predictedGender, datSample$Female)

predictedGender 0

male 16

## Does DNAm age acceleration relate to autism disease status, cause of death, postmortem interval?

attach(datSample)

# Here I consider two measures of age acceleration.

# The first acceleration meausure is based on the difference.

AgeAccelerationDiff=DNAmAge- datSample$Age

# The second acceleration meausure equals the residual resulting

# from regressing DNAmAge on chronological age

restNonMissing= !is.na(DNAmAge) & !is.na(Age)

AgeAccelerationResidual=rep(NA, length(Age) )

if (sum(restNonMissing,na.rm=TRUE) >3 ){

AgeAccelerationResidual[restNonMissing]=residuals(lm(as.numeric(datout$DNAmAge)~as.numeric(datSample$Age), subset= restNonMissing))

}

DiseaseLabel=ifelse(datSample$diseaseStatus==1,"A","C")

DiseaseColor=ifelse(datSample$diseaseStatus==1,"red","black")

par(mfrow=c(3,3))

verboseScatterplot(DNAmAge, Age,xlab="DNAm Age", ylab="Chronological Age",main=paste("A err=", medianAbsDev1),type="n" );abline(0,1)

text(DNAmAge, Age, lab= DiseaseLabel, col= DiseaseColor );abline(0,1)

verboseBarplot(Age, DiseaseLabel,xlab="Disease Status",main="B Age",ylab="Chronological Age" ,col=c("red","grey") )

verboseBarplot(DNAmAge, DiseaseLabel,xlab="Disease Status",main="C DNAm Age",ylab="DNAm Age",col=c("red","grey") )

verboseBarplot(AgeAccelerationDiff, DiseaseLabel,main="D Disease Status", xlab="Disease Status",col=c("red","grey") )

verboseBarplot(AgeAccelerationDiff, CauseofDeath, main="E Cause of Death", xlab="Cause of Death")

verboseScatterplot(PostMortemInterval, AgeAccelerationDiff, xlab="Post Mortem Interval",main=paste("F PMI, err=", medianAbsDev1),type="n")

text(PostMortemInterval, AgeAccelerationDiff, lab= DiseaseLabel, col= DiseaseColor )

verboseBarplot(AgeAccelerationResidual, DiseaseLabel,main="G Disease Status", xlab="Disease Status", col=c("red","grey") )

verboseBarplot(AgeAccelerationResidual, CauseofDeath, main="H Cause of Death", xlab="Cause of Death")

verboseScatterplot(PostMortemInterval, AgeAccelerationResidual, xlab="Post Mortem Interval",main=paste("I PMI, err=", medianAbsDev1),type="n")

text(PostMortemInterval, AgeAccelerationResidual, lab= DiseaseLabel, col= DiseaseColor )

**Caption: Age acceleration versus autism status and other variables**

A) Chronological age versus DNA methylation age. Points are colored and labeled according to autism status. Note that the DNAm age lines is highly related to chronological age for autism samples (labeled A) but control samples show signs of accelerated aging (around age 40). B) Chronological age (y-axis) is not significantly related to autism status. C) The same applies to DNAmAge. D,G) A marginally significant association can be observed between autism status and either measure of age acceleration. E,H) No significant relationship can be observed between cause of death and either measure of age acceleration and cause of death. F,I) The same applies to post mortem interval

Interpretation: There is suggestive evidence that autism samples exhibit smaller age acceleration than control samples. These results lead to the hypothesis that occipital cortex samples from middle aged autism patients are slightly younger than expected based on age matched controls.

CAVEAT: This study is under-powered and hidden confounders may affect the results. There is a high probability that this finding represents a false positive.

Exercise 1 : Secure an independent and larger data set involving (occipital) cortex samples from roughly age matched autism cases and controls. Make sure to include many samples from middle subjects.

a) Run the samples on the Illumina Inf 450K or 27K platform or any other platform that measures the 21k probes.

b) Refute/validate the hypotheses mentioned above. findings.

c) If the results validate, quickly write up the results.

# The end
