# Supplementary figures and images for: Erratum to: DNA methylation age of human tissues and cell types
Source: Genome Biol. 2015 May 13;16(1):96. doi: 10.1186/s13059-015-0649-6 (PMC4427927; doi:10.1186/s13059-015-0649-6)

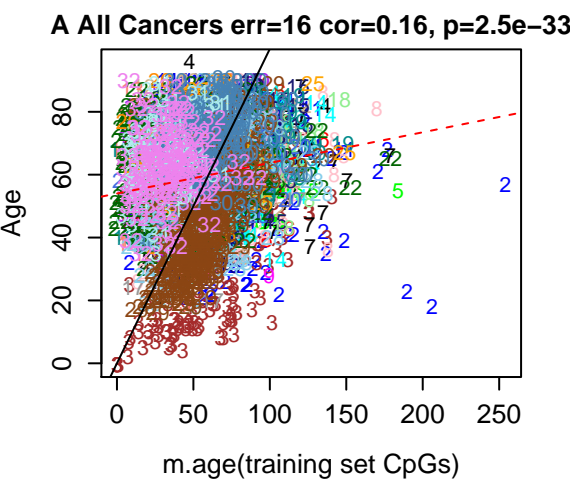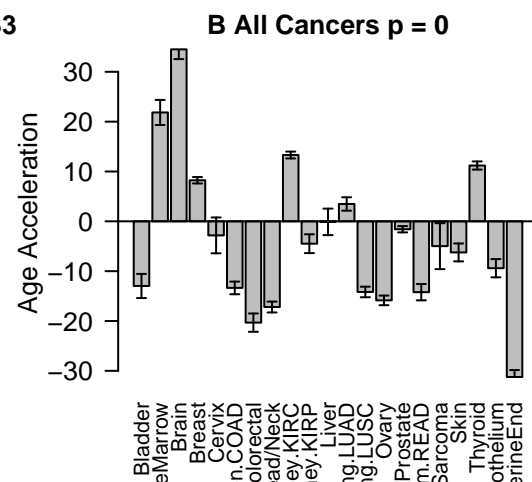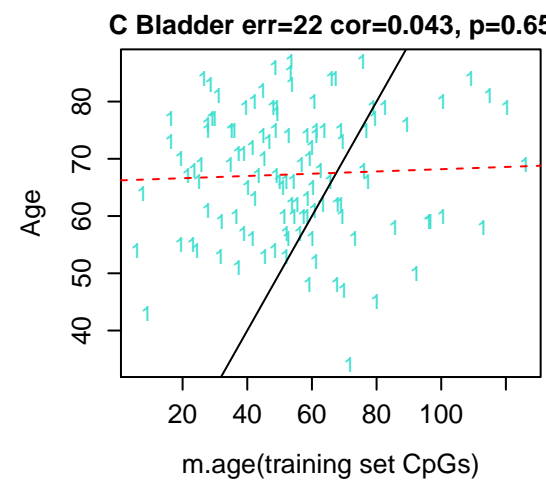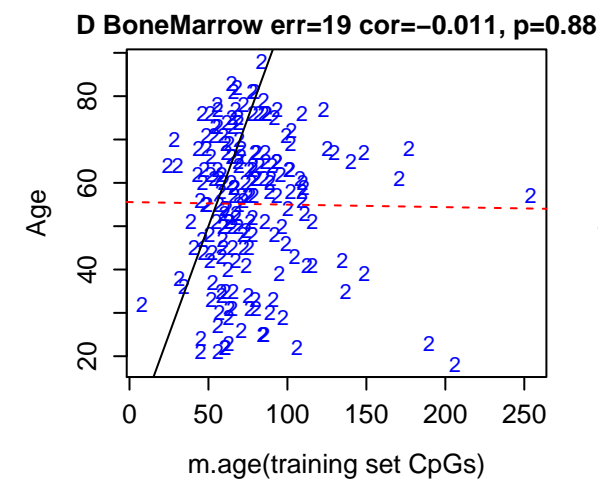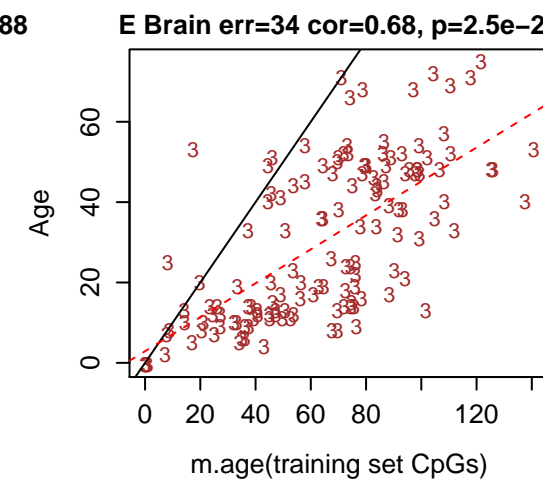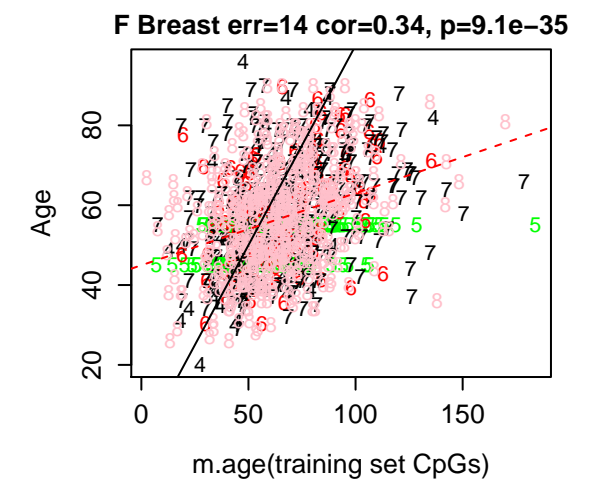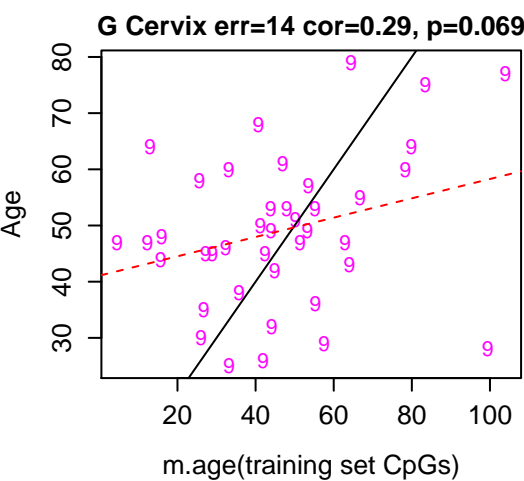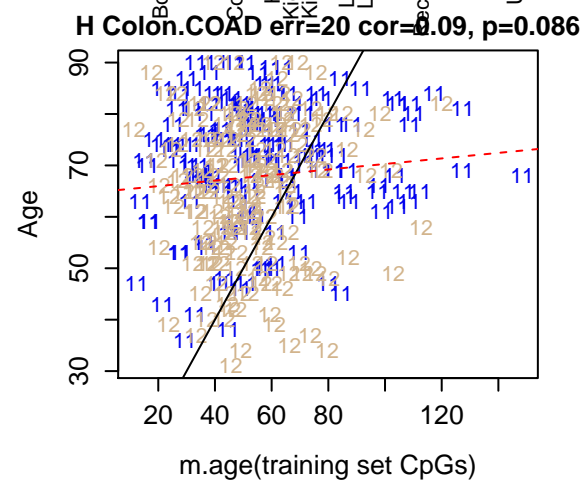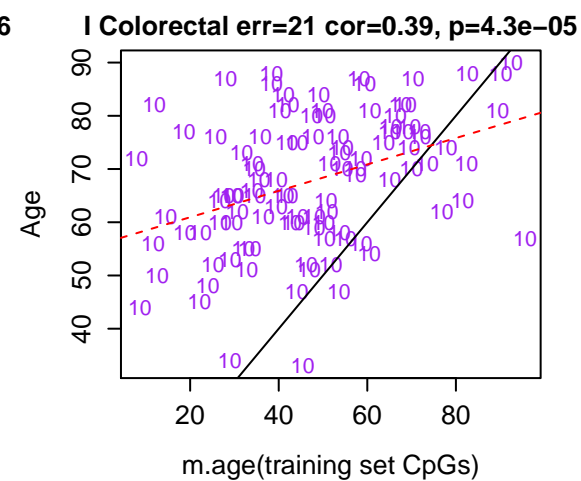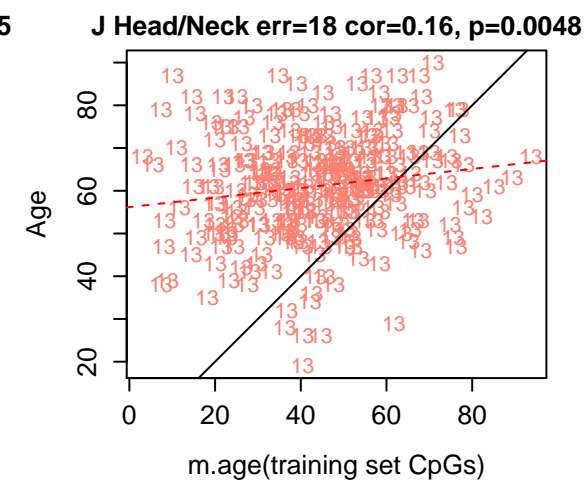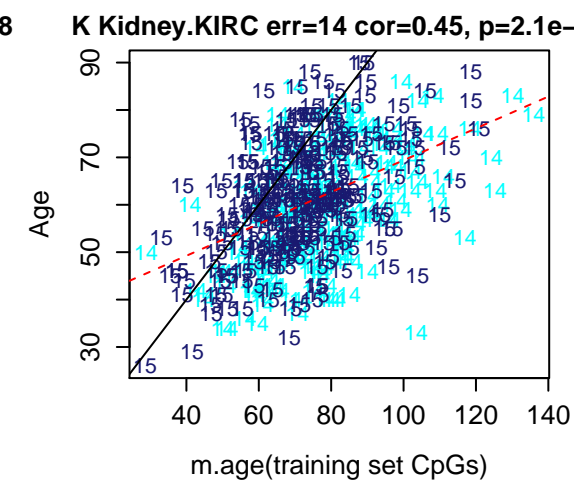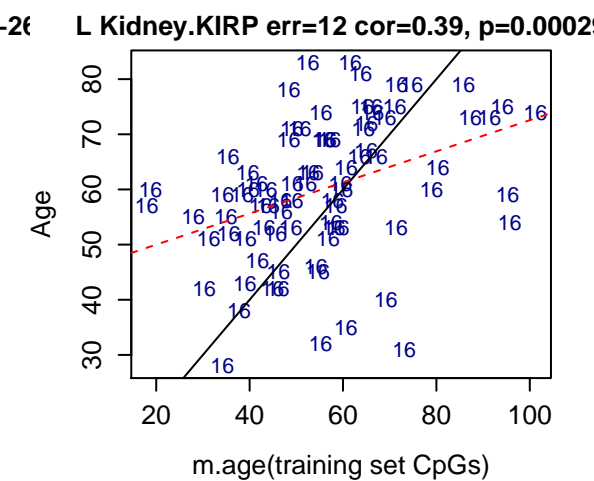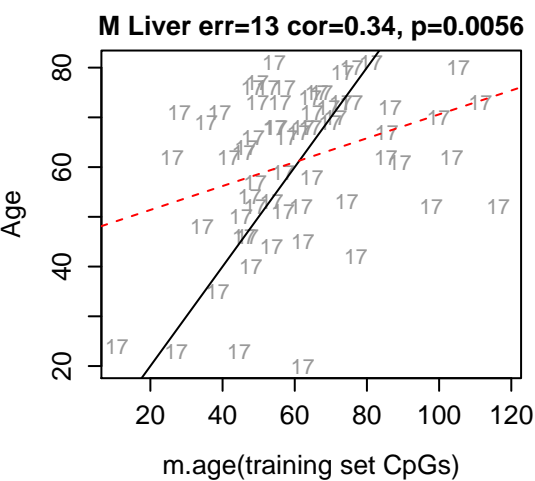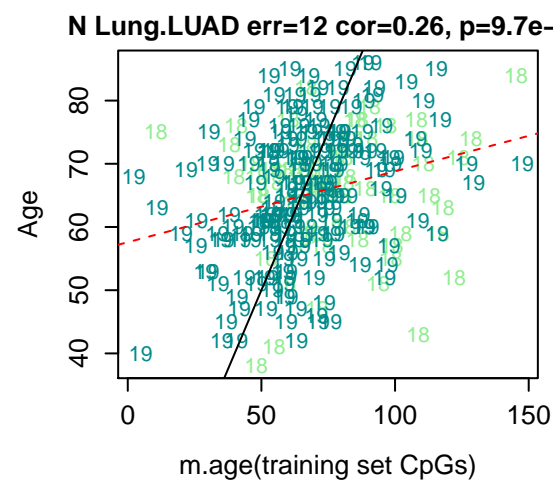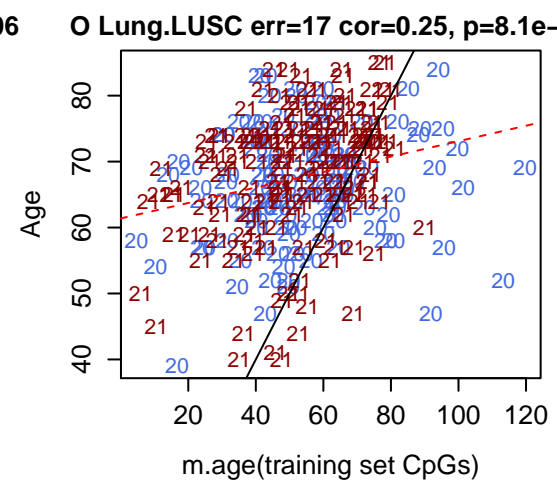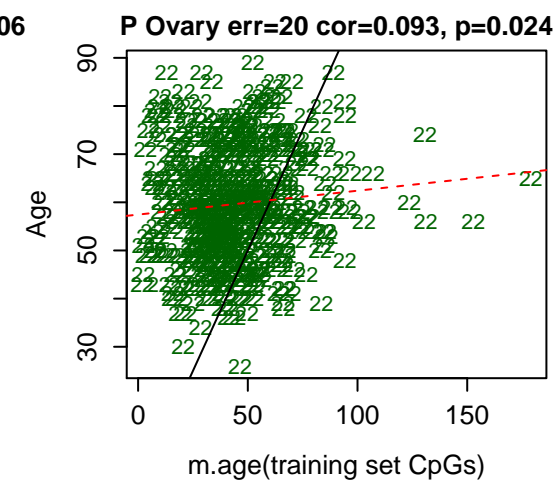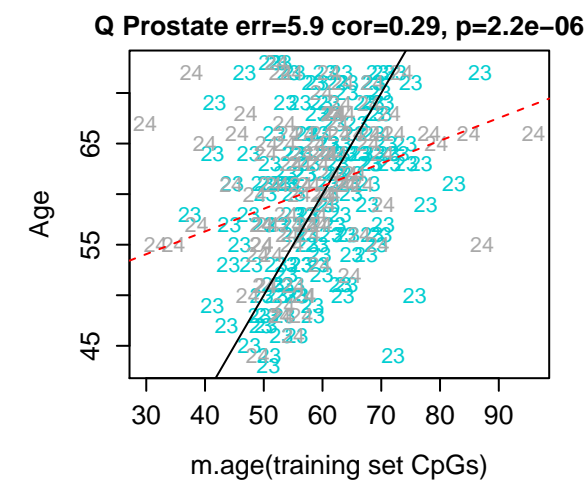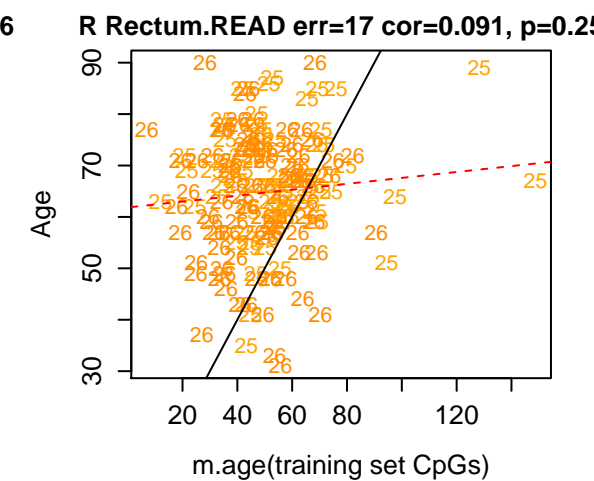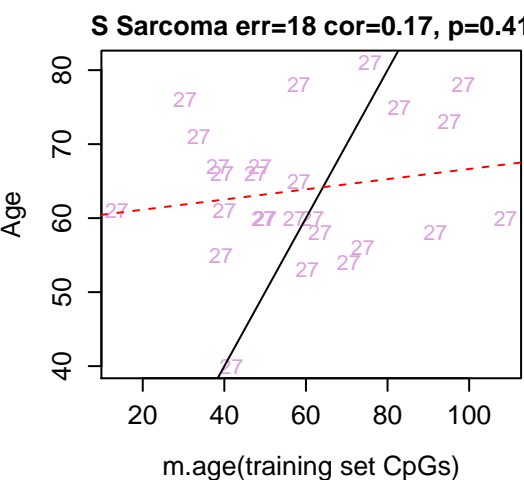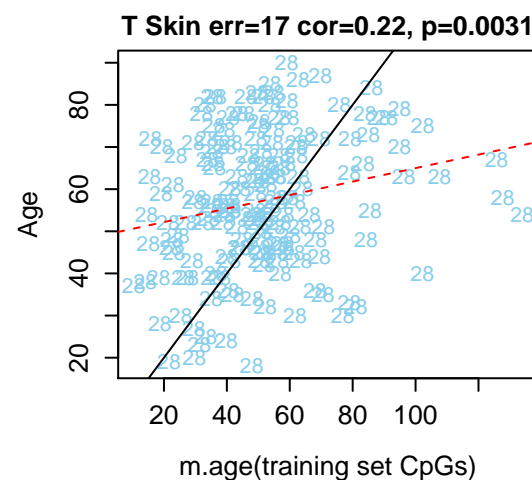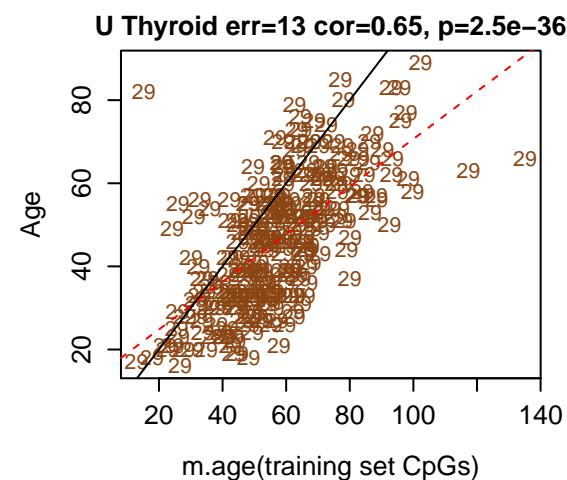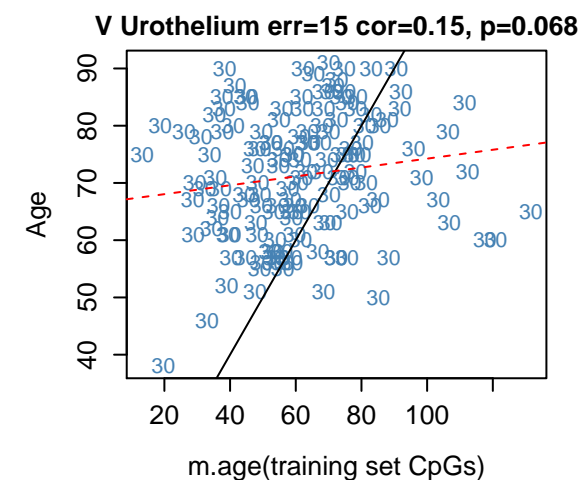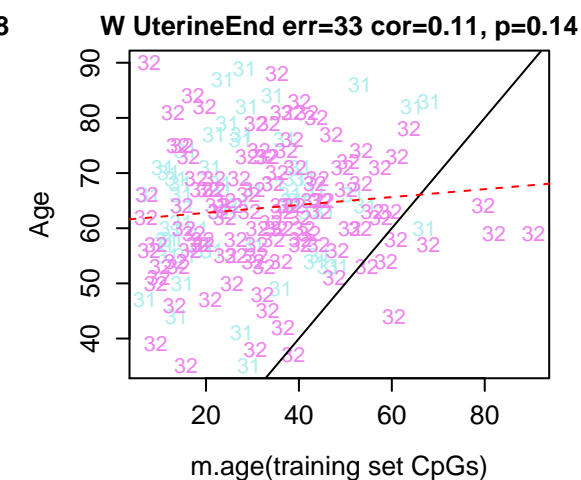

Supplement: Additional file 2: — DNAm age versus chronological age in cancer. Each point corresponds to a DNA methylation sample (cancer sample from a human subject). Points are colored and labelled according to the underlying cancer data sets as described in Additional file 1. A) Across all cancer data sets, there is only a weak correlation (cor=0.16, p=2.5E-33) between DNAm age (x-axis) and chronological patient age (y-axis). B) Mean age acceleration (y-axis) versus cancer type. C-W) Results for individual cancers/affected tissues. Several cancer tissues maintain moderately large age correlations including E) brain, U) thyroid, K,L) kidney, M) liver, I) colorectal, and F) breast cancer. [file 13059_2015_649_MOESM2_ESM.pdf]

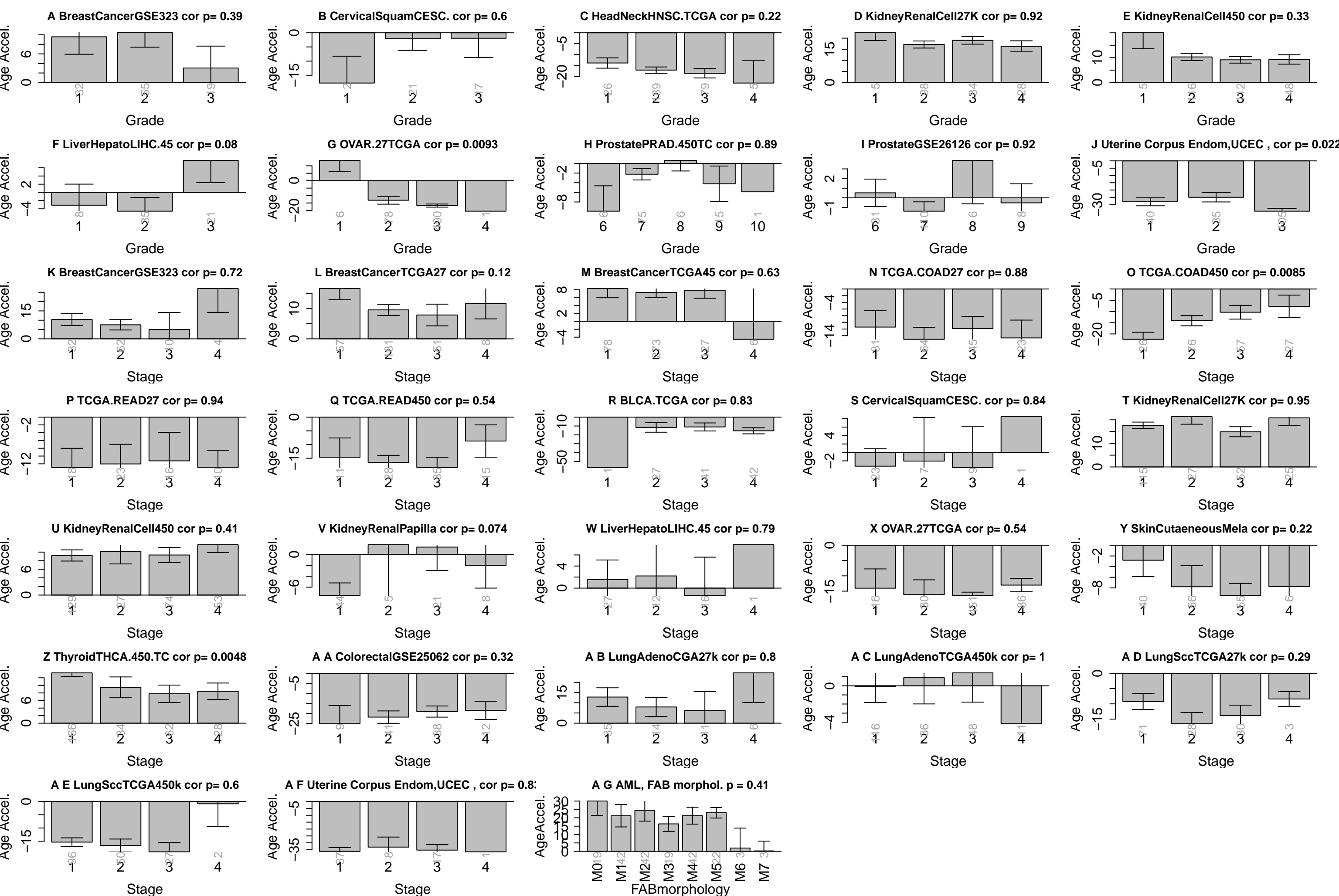

Supplement: Additional file 3: — Age acceleration versus tumor grade and stage. Panels correspond to the cancer data sets described in Additional file 1. Nominally significant negative correlations between grade and age acceleration can be observed in ovarian serous cystadenocarcinoma (panel G) and uterine corpus endometroids (panel J). A nominally significant positive correlation between stage and age acceleration can be observed for colon adenocarcinoma (panel O). (Z) A significant negative correlation between stage and age acceleration can be observed in thyroid cancer. Since grade and stage are often considered as ordinal variables, correlation test p-values are reported in all panels except the last. H) For prostate cancer, the x-axis reports the Gleason sum score. The last panel shows that mean age acceleration in acute myeloid leukemia is not significantly related to French American British (FAB) morphology but some groups (notably M6 and M7) are very small (rotated grey numbers). [file 13059_2015_649_MOESM3_ESM.pdf]

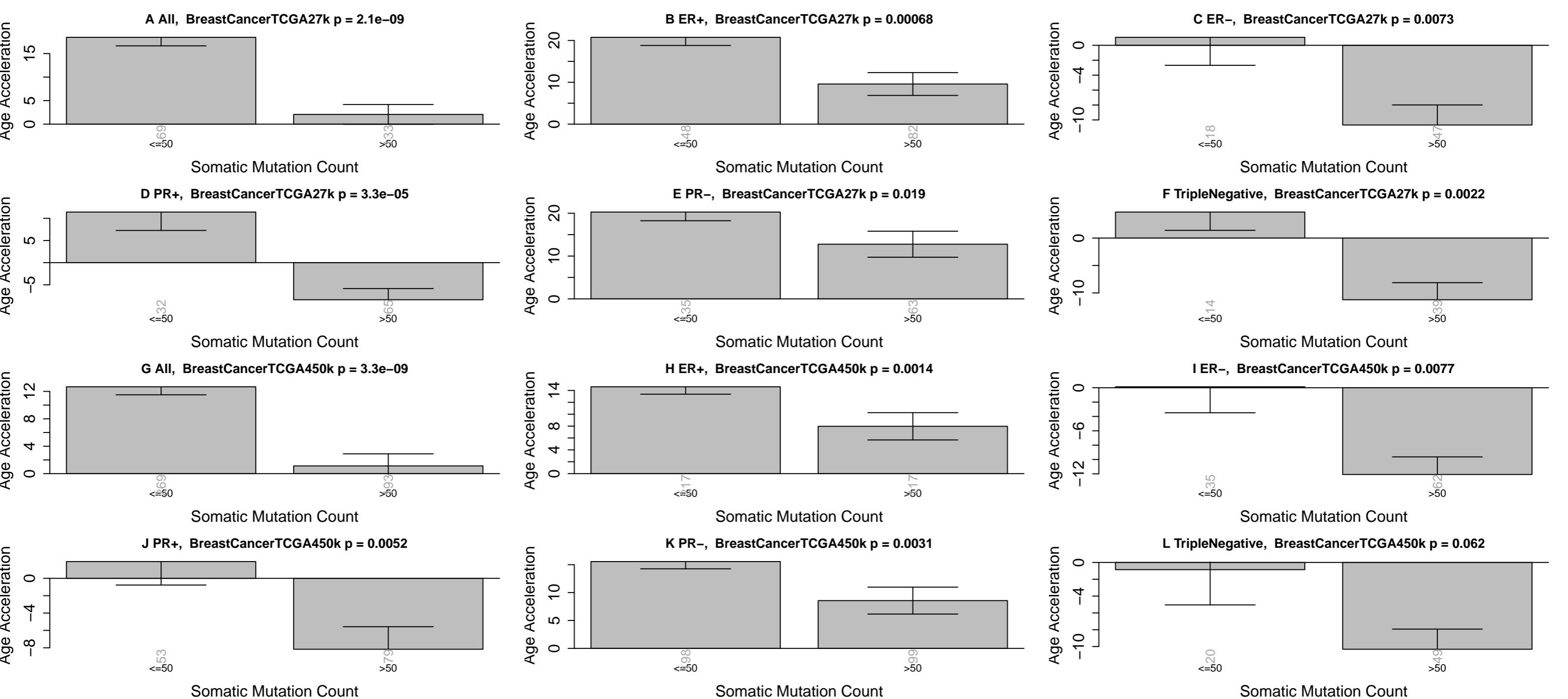

Supplement: Additional file 4: — Age acceleration versus mutation count status in breast cancer. Mutation count status (x-axis) was defined by assigning tumor samples to the high mutation count group if their number of somatic mutations was larger than 50. Other thresholds lead to similar results. A-F) and G-L) report findings for Illumina 27K and 450K data, respectively. A, G) The barplots show that mean age acceleration (y-axis) is lower in breast cancer samples with high mutation count (compared to those samples whose somatic mutation count is less than 50). This result can also be found in ER+ (panels B,H), ER- (C,I), PR+ (D,J), PR- (E,K), and triple negative (F,L) breast cancer samples. [file 13059_2015_649_MOESM4_ESM.pdf]

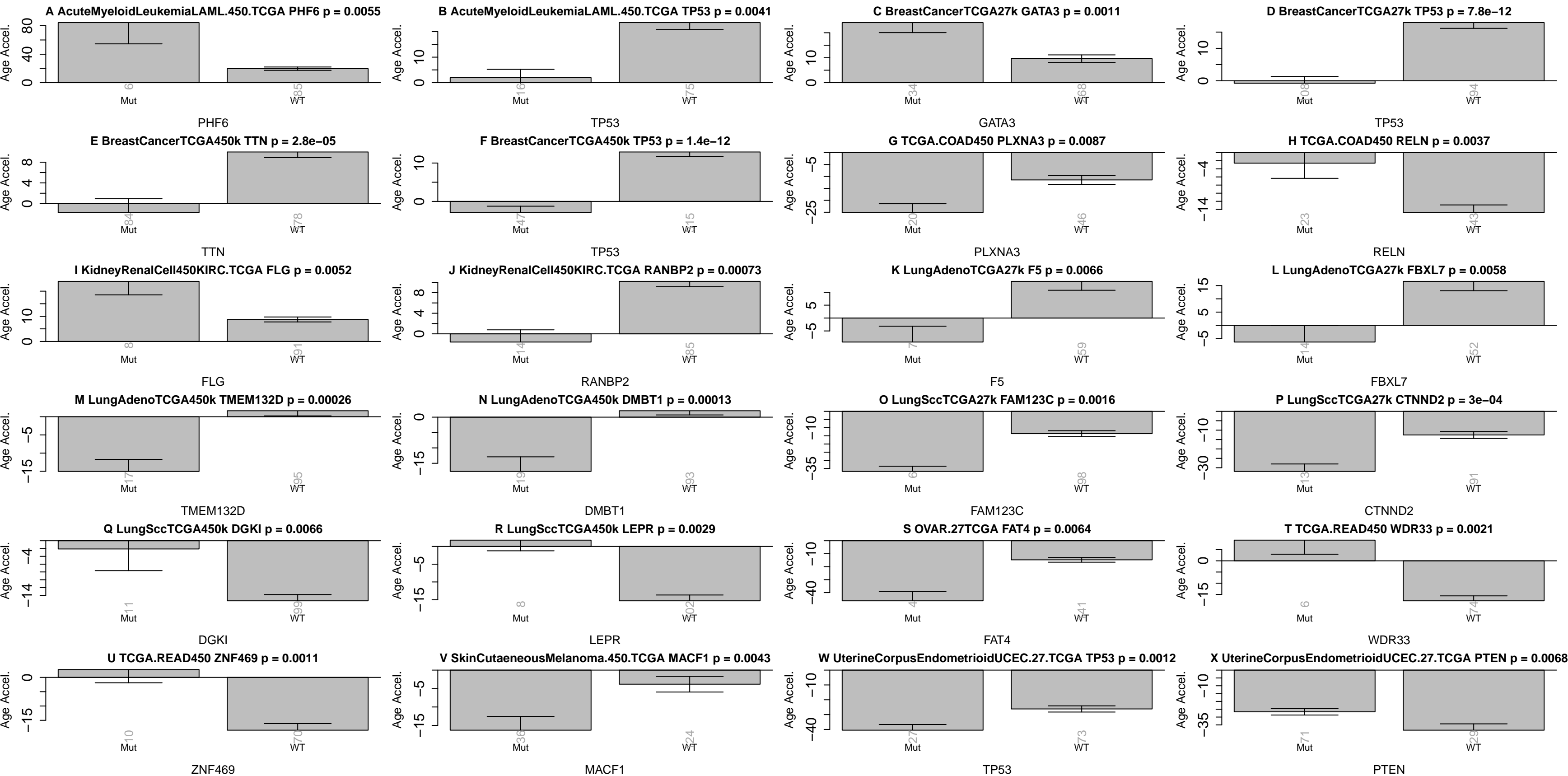

Supplement: Additional file 5: — Selected significant gene mutations versus age acceleration. The TCGA data sets were stratified by cancer type and Illumina platform. Mean age acceleration (y-axis) versus mutation status (x-axis) for up to two of the most significant genes per data set. Note that age acceleration in bone marrow (AML) was most highly related to mutation in the following 2 genes: PHF6 and TP53. Age acceleration in the two breast cancer data was most highly related to mutations in GATA3, TP53, and TTN. Strikingly, TP53 was among the top 2 most significant mutated genes in four out of 13 cancer data sets. [file 13059_2015_649_MOESM5_ESM.pdf]

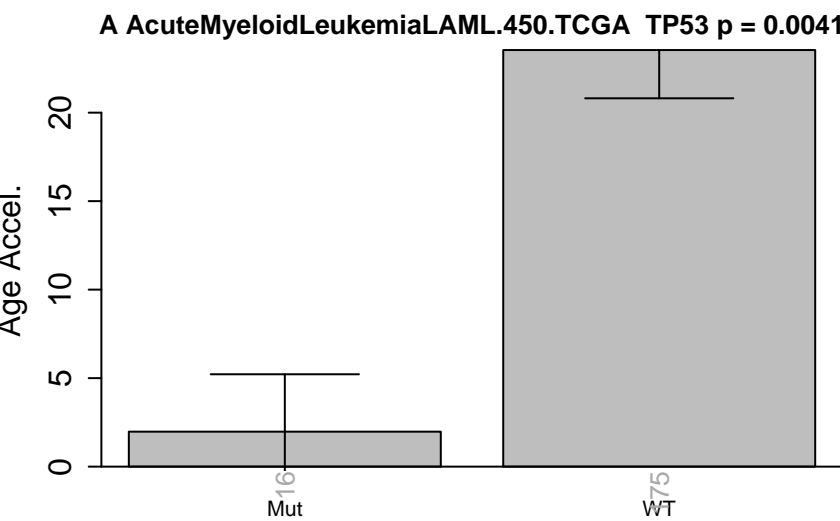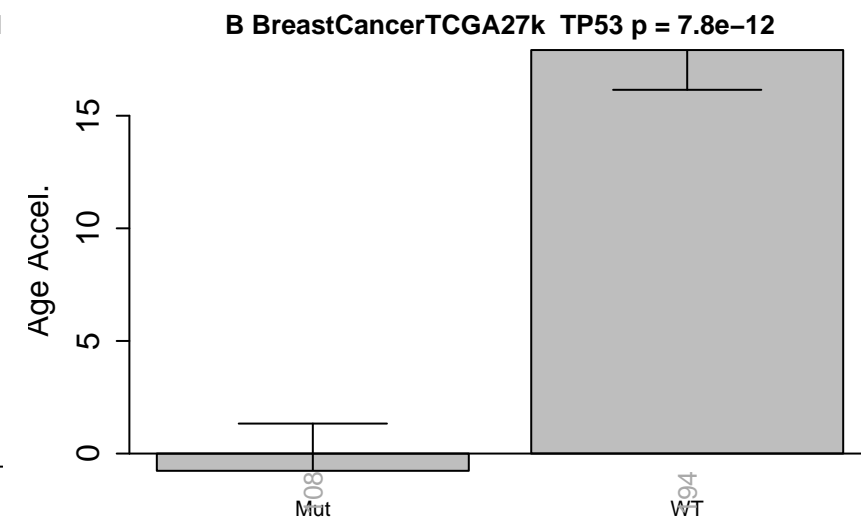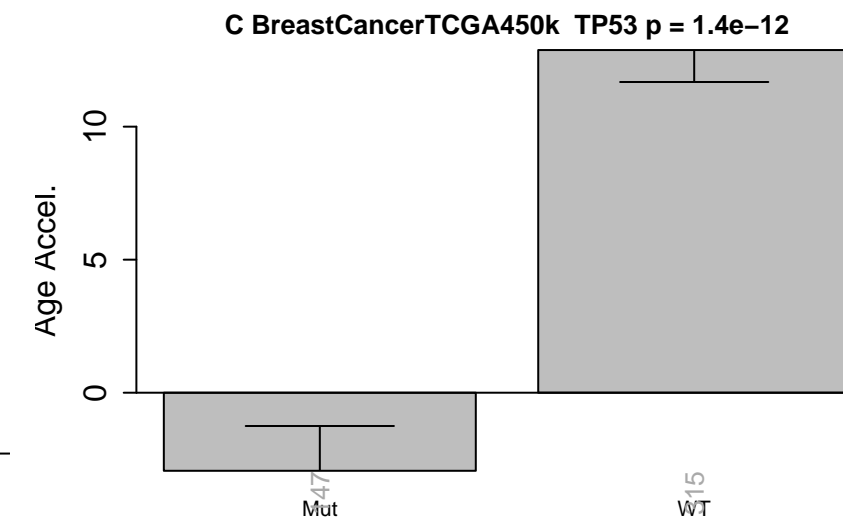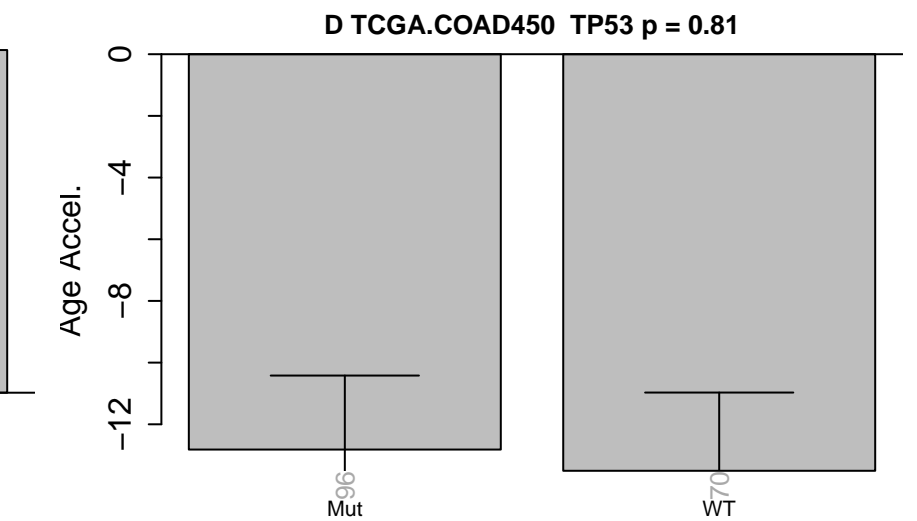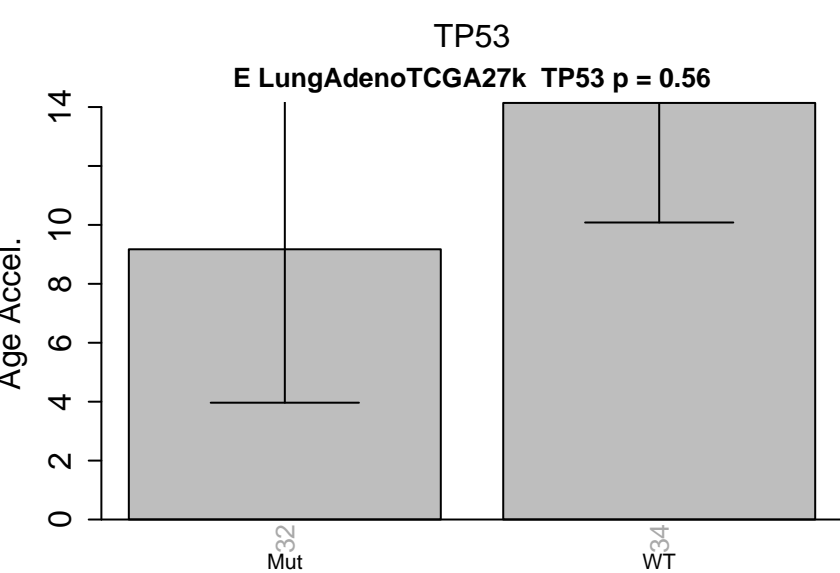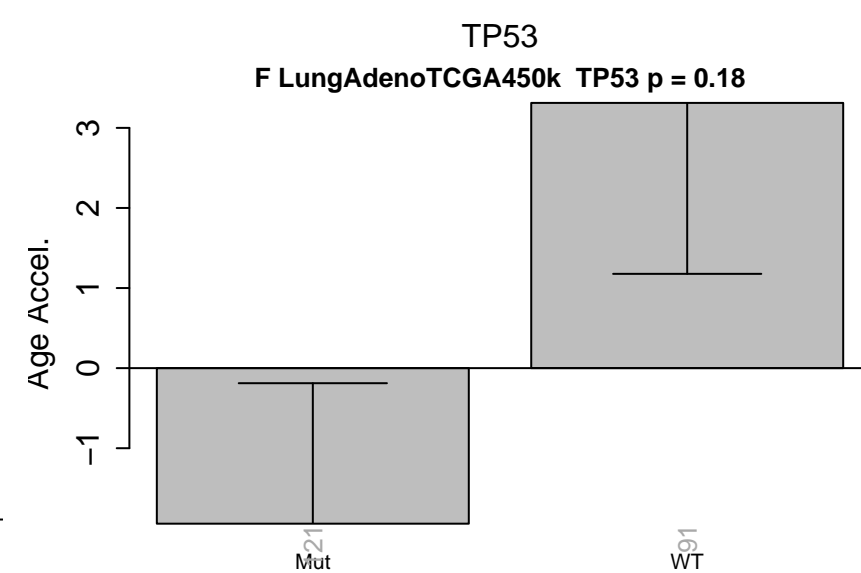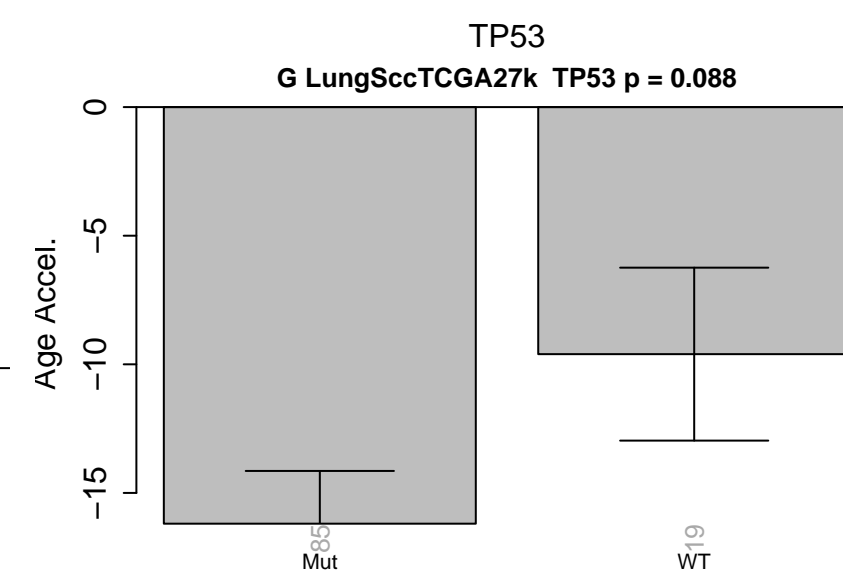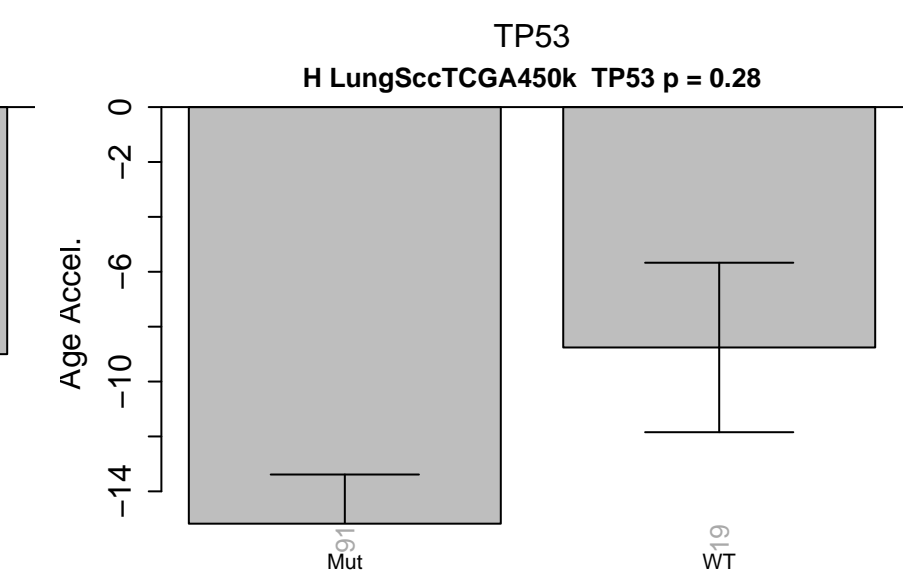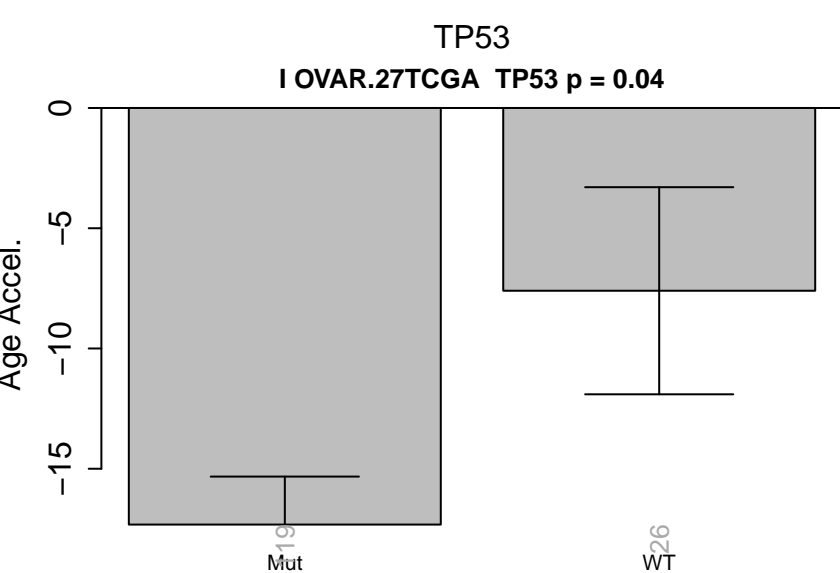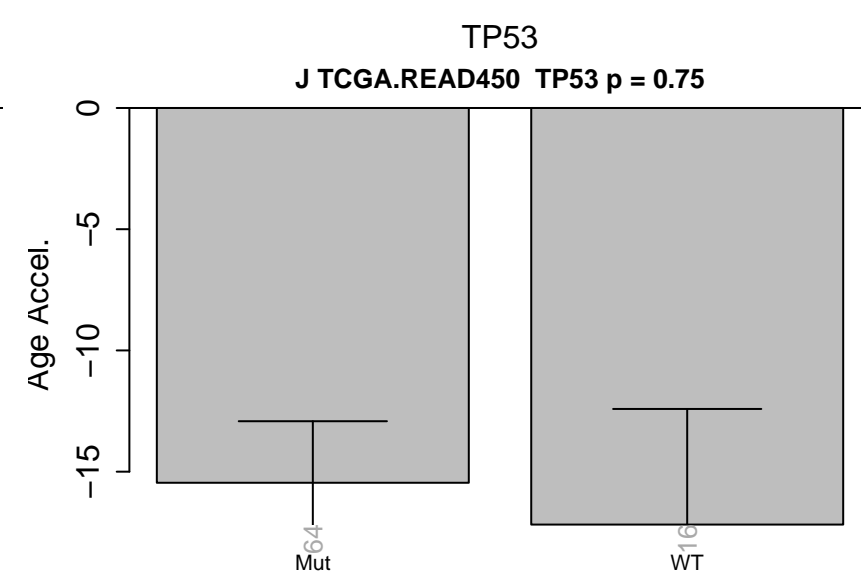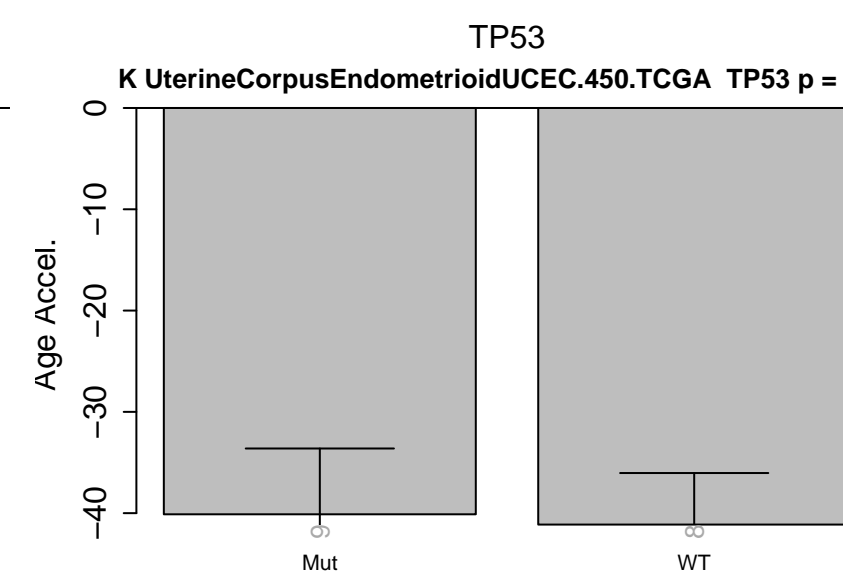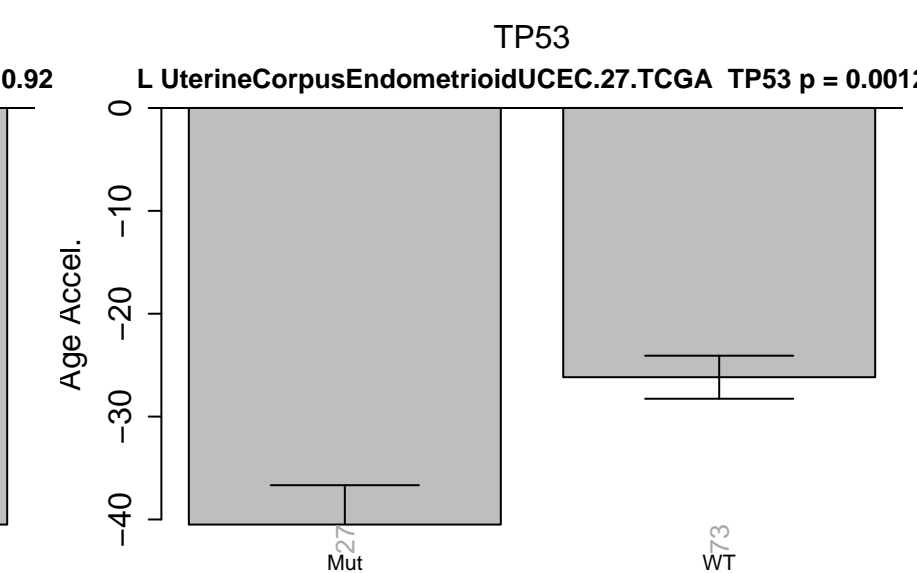

Supplement: Additional file 6: — Effect of TP53 mutation on age acceleration. Mutations in TP53 are associated with significantly lower age acceleration in 5 cancers: including AML, breast cancer, ovarian serous cystadenocarcinoma, and uterine corpus endometrioid. Marginally significant results could be observed in lung squamous cell carcinoma (p=0.088 for the 27K data but not for the 450K data). [file 13059_2015_649_MOESM6_ESM.pdf]
